# Ferroptotic stress promotes macrophages against intracellular bacteria

**DOI:** 10.7150/thno.66663

**Published:** 2022-02-21

**Authors:** Ruonan Ma, Ling Fang, Lei Chen, Xiaonan Wang, Jing Jiang, Lizeng Gao

**Affiliations:** 1 Institute of Translational Medicine, Department of Pharmacology, School of Medicine, Yangzhou University, China.; 2 CAS Engineering Laboratory for Nanozyme, Institute of Biophysics, Chinese Academy of Sciences, China.; 3 Joint Laboratory of Nanozymes in Zhengzhou University, Academy of Medical Sciences, Zhengzhou University, China.

**Keywords:** Macrophages, intracellular bacteria, ferroptotic stress, ferroportin, ferrous iron, ferroptosis-like bacterial death

## Abstract

**Rational**: Intracellular bacterial survival is a major factor causing chronic or recurrent infection, leading to the failure of both host defense and/or antibiotic treatment. However, the elimination of intracellular bacteria is challenging as they are protected from antibiotics and host immune attack. Recent studies have indicated that iron helps macrophages against intracellular bacteria, contradictory to traditional “nutritional immunity”, in which iron is considered a key nutrient for bacterial survival in host cells. However, how iron facilitates intracellular bacterial death has not been fully clarified. In this study, we found that ferroptotic stress can help macrophages suppress intracellular bacteria by reversing the importation of ferrous iron into bacterial vacuoles via ferroportin and thereby inducing *in situ* ferroptosis-like bacterial death.

**Methods**: A macrophage model of bacterial invasion was established to monitor dynamic changes in ferroptotic hallmarks, including ferrous iron and lipid peroxidation. Ferroptosis inducers and inhibitors were added to the model to evaluate the relationship between ferroptotic stress and intracellular bacterial survival. We then determined the spatiotemporal distributions of ferroportin, ferrous iron, and lipid peroxidation in macrophages and intracellular bacteria. A bacterial infection mouse model was established to evaluate the therapeutic effects of drugs that regulate ferroptotic stress.

**Results**: Ferrous iron and lipid peroxidation increased sharply in the early stage of bacterial infection in the macrophages, then decreased to normal levels in the late stage of infection. The addition of ferroptosis inducers (ras-selective lethal small molecule 3, sulfasalazine, and acetaminophen) in macrophages promoted intracellular bacterial suppression. Further studies revealed that ferrous iron could be delivered to the intracellular bacterial compartment via inward ferroportin transportation, where ferrous iron induced ferroptosis-like death of bacteria. In addition, ferroptotic stress declined to normal levels in the late stage of infection by regulating iron-related pathways in the macrophages. Importantly, we found that enhancing ferroptotic stress with a ferroptosis inducer (sulfasalazine) successfully suppressed bacteria in the mouse infection models.

**Conclusions**: Our study suggests that the spatiotemporal response to ferroptosis stress is an efficient pathway for macrophage defense against bacterial invasion, and targeting ferroptosis may achieve therapeutic targets for infectious diseases challenged by intracellular pathogens.

## Introduction

Persistent infections due to intracellular bacteria are a serious problem in recovery. As the first line of defense against bacterial infection, macrophages have evolved versatile strategies to combat intracellular bacteria 1, thus providing a potential reservoir for the development of new antimicrobials. A critical strategy in macrophages is to limit iron access to bacteria, i.e., “nutritional immunity” 2, 3. Upon infection, macrophages limit the supply of iron to the invading bacteria by sequestering intracellular iron and maintaining low extracellular iron levels 4. This is achieved by importing extracellular iron into their cytoplasm with iron-binding proteins (such as transferrin and lactoferrin 5) and by binding hepcidin to ferroportin (FPN) to block iron export 6-12. However, these processes can lead to high intracellular iron, which may be utilized by intracellular bacteria. To avoid this, macrophages produce inhibitors, such as lipocalin-2 (Lcn2), to block iron delivery to bacterial siderophores 13. In contrast, recent studies have demonstrated that iron is required for macrophages to suppress intracellular *Salmonella typhimurium*, as FPN imports iron into *Salmonella*-containing vacuoles (SCV) and locally induces reactive oxygen species (ROS) via the sequential reactions of NADH oxidase 2 (Nox2) and Fenton catalysis 14. Therefore, iron appears to play paradoxical roles as a nutrient and/or ROS generator in macrophages upon bacterial infection.

We previously demonstrated that iron-containing nanomaterials (e.g., Fe_3_O_4_ and Fe_3_S_4_) can directly kill bacteria by releasing ferrous iron (not ferric iron) to induce lipid peroxidation in bacteria 15-17. These hallmarks of ferrous iron dependence and lipid peroxidation in bacteria are the same as those of ferroptosis, which is a novel form of programmed cell death in mammalian cells. Importantly, we found that intracellular *Salmonella enteritidis* and *Staphylococcus aureus* can be suppressed by the addition of iron-containing nanomaterials or ferrous iron to macrophages. Notably, only ferrous iron, not ferric iron, exhibits antibacterial activity, consistent with the phenomenon that most bacterial siderophores chelate and deliver ferric iron as an iron nutrient resource 18. Because iron accumulates via FPN regulation during bacterial invasion in macrophages, we hypothesized that ferroptosis-related actions may be activated as a general approach to suppress intracellular pathogens in host defense.

To prove this, we first monitored dynamic changes in ferroptosis hallmarks, including iron (both ferrous and ferric iron) and lipid peroxidation, in macrophages upon bacterial invasion over time. We then assessed the correlation between ferroptotic stress with ferroptosis inducers and inhibitors and survival rate of intracellular bacteria in RAW264.7 cells and primary bone marrow protomacrophages (BMMs). We also determined the spatiotemporal distributions of iron, FPN, and lipid peroxide with intracellular bacteria. Additionally, the therapeutic efficacy of targeting ferroptotic stress regulation was assessed in established mouse models bearing bacterial infections.

## Methods

### Ethics statement

All animal studies were performed following the protocols approved by the Institutional Animal Care and Use Committee of Yangzhou University and Institutional Animal Care and Use Committee of the Institute of Biophysics, Chinese Academy of Sciences. All mice were discarded according to standard approved protocols after the experiments were completed.

### Internalization of bacteria by macrophage and intracellular assays

RAW264.7 mouse macrophages were obtained from the Cell Bank of the Type Culture Collection of the Chinese Academy of Sciences (Shanghai, China). The cells were cultured in Roswell Park Memorial Institute (RPMI) 1640 medium (BI), supplemented with 20% fetal bovine serum (FBS) and 1% penicillin/streptomycin (Corning) for 12 h at 37 °C under 5% CO_2_. The cells were collected at the logarithmic growth stage, resuspended in the same complete culture medium, and inoculated (7 × 10^5^ cells) into a 6-well plate containing 1 mL of complete culture medium (RPMI 1640 + 20% FBS + 1% penicillin/streptomycin). Cells were then cultured at 37 °C under 5% CO_2_ to 80%-90% confluence, thus achieving a cell count of 1 × 10^6^ cells/well. Single colonies of *Staphylococcus aureus* (ATCC 29213) (*S. aureus*) and* Escherichia coli* (CMCC (B)44102) (*E. coli*) in lysogeny broth (LB) on solid agar plates were randomly selected and inoculated in 5 mL of LB liquid culture medium and cultured at 37 °C for 12 h under 180 rpm rotation. The next day, 50 µL of the bacterial inoculation was diluted with 4 950 µL of fresh LB medium and cultured at 37℃ for 3 h under 180 rpm until OD600 reached 0.8. The bacterial precipitate was obtained by centrifugation at 3 600 rpm for 5 min at 4 °C and diluted to 2 × 10^8^ colony forming units (CFUs)/mL with 2.5 mL of RPMI 1640 culture medium. The RAW264.7 cells were then infected with 100 µL of *S. aureus* and *E. coli* for 1 h, respectively (MOI = 20:1), and extracellular bacteria were killed with gentamicin (200 μg/mL) for 3 h. The cells were then washed three times with phosphate-buffered saline (PBS) and cultured at 37 °C under 5% CO_2_ in 1 mL of RPMI 1640 containing 1% FBS for 1, 3, 6, 12 and 24 h. Iron levels (i.e., total iron, ferrous iron (Fe^2+^), and ferric iron (Fe^3+^)) in the cells were measured using a Micro Serum Iron Concentration Assay Kit (Solarbio). Intracellular ferrous iron was also detected with a FeRhoNox^TM^-1 fluorescent probe (GORYO Chemical). Then, 200 µL of 5 µM FeRhoNox^TM^-1 working solution was added to the cells, followed by 45 min of incubation at 37 °C. Intracellular ferrous fluorescence intensity was measured using a Zeiss confocal microscope (Zeiss LSM980) or flow cytometry (BD FACSCalibur, USA). Total glutathione (GSH), reduced glutathione GSH, and oxidized glutathione disulfide (GSSG) were detected using GSH and GSSG Assay kits (Beyotime Biotechnology). Intracellular lipid peroxidation levels were detected with a BODIPY 581/591 C11 probe, with 200 µL of 2 µM BODIPY 581/591 C11 probe working solution added to the cells with 30 min of incubation at 37 °C. Fluorescence intensity of lipid ROS was detected by flow cytometry (BD FACSCalibur). Changes in mitochondrial membrane potential (MMP) were detected with a JC-1 fluorescent probe (Beyotime Biotechnology). Fluorescence of J-aggregates was measured using a multi-scan spectrum with excitation at 525 nm and emission at 590 nm. Cell activity following bacterial infection was assayed with a Cell Counting Kit-8 (CCK-8) (DOJINDO).

### Extracellular bacterial killing with gentamicin

Single colonies of *S. aureus* (ATCC 29213), *E. coli* (CMCC (B)44102), and *Salmonella pullorum* (S06004, a kind gift from Prof. Xinan Jiao's laboratory in Yangzhou University 19) (*S. pullorum*) on LB solid agar plates were randomly selected and inoculated in 5 mL of LB liquid culture medium and cultured at 37 °C for 12 h under 180 rpm rotation. The following day, 50 µL of the bacterial inoculation was diluted with 4 950 µL of fresh LB medium and cultured at 37 °C for 3 h at 180 rpm until OD600 reached 0.8. The bacterial precipitate was obtained by centrifugation at 3 600 rpm for 5 min at 4 °C and diluted to 2 × 10^8^ CFUs/mL with 2.5 mL of RPMI 1640 culture medium. Then, 100 μL of bacterial inoculation was mixed with 900 μL of RPMI 1640 containing 1% FBS as the control. Another 100 μL of bacterial inoculation was mixed with 900 μL of RPMI 1640 containing gentamicin (200 μg/mL) and 1% FBS as the experimental group. After incubation at 37℃ for 1 h and 3 h, bacterial viability was checked by plating bacteria at a proper dilution and calculating the bacterial number in CFUs/mL.

The effect of gentamicin on intracellular bacterial activity was then examined. RAW264.7 cells were inoculated in 6-well plates containing 1 mL of complete culture solution (RPMI 1640 + 20% FBS + 1% penicillin/streptomycin). At the logarithmic growth phase, the RAW264.7 cells were infected with *S. aureus*, *E. coli*, and *S. pullorum* at MOI = 20:1 for 1 h, respectively, with the extracellular bacteria killed with gentamicin (200 μg/mL) for 3 h. After washing three times with PBS, the cells were cultured at 37 °C under 5% CO_2_ in 1 mL of RPMI 1640 containing 1% FBS for 0.5, 1, and 2 h. A sample of the cells was lysed using 1 mL of buffer containing 0.4% Triton X-100. The number of viable bacteria was counted by plating serial dilutions of bacteria on LB solid medium.

### *In vitro* antibacterial assays

Single colonies of *S. aureus* (ATCC 29213), *E. coli* (CMCC (B)44102), and *Salmonella pullorum* (S06004, kind gift from Prof. Xinan Jiao's laboratory in Yangzhou University 19) on LB solid agar plates were randomly selected and inoculated in 5 mL of LB liquid medium and cultured at 37 °C for 12 h under 180 rpm rotation. The next day, 50 µL of the bacterial inoculation was diluted with 4 950 µL of fresh LB medium and cultured at 37 °C for 3 h at 180 rpm until OD600 reached 0.8. Then, 100 μL of bacterial inoculation was mixed with H_2_O (900 μL) as the control. Another 100 μL of bacterial inoculation was mixed with Fe^2+^ (100 μL) (12.5, 25, 50, 100, and 200 µM) and H_2_O (800 μL) as the experimental group. After incubation at 37 °C for 30 min, bacterial viability was checked by plating bacteria at a proper dilution and calculating the bacterial number in CFUs/mL. Inhibition assays with 0.4% Triton X-100 (Beyotime Biotechnology), Ras-selective lethal small molecule 3 (RSL3) (TargetMol), sulfasalazine (SSZ) (TargetMol), acetaminophen (APAP) (TargetMol), ethylenediaminetetraacetic acid (EDTA), deferoxamine mesylate (DFO) (TargetMol), ferrostatin-1 (Fer-1) (TargetMol), Fe^3+^, and hepcidin (Abcam) were conducted using the same system and procedure. Inhibition assays with Fe^2+^ in combination with EDTA or Fer-1 also used under the same system and procedure, although in addition to 100 μL of Fe^2+^ (12.5 µM), an additional 100 μL of EDTA or Fer-1 and 700 μL of H_2_O were added to maintain the system at 1 mL.

### Bacterial characterization with scanning (SEM) and transmission electron microscopy (TEM)

Single colonies of *S. aureus* and *E. coli* on LB solid agar plates were randomly selected and inoculated in 5 mL of LB liquid medium and cultured at 37 °C for 12 h under 180 rpm rotation. The next day, 50 µL of the bacterial inoculation was diluted with 4 950 µL of fresh LB medium and cultured at 37 °C for 3 h at 180 rpm until OD600 reached 0.8. The bacterial precipitate (1 mL) was obtained by centrifugation at 3 600 rpm for 5 min at 4 °C and diluted with H_2_O (900 μL). Then, 100 μL of Fe^2+^ (50 µM) was mixed with *S. aureus* (900 μL) and *E. coli* (900 μL) at 37 °C for 30 min, respectively. Morphology was examined by SEM. First, the bacterial suspension was resuspended in glutaraldehyde (2.5%, Sigma-Aldrich) for 24 h at 4 °C under dark conditions. Bacterial cells were then washed and treated with ethanol gradient dehydration (30%, 50%, 70%, 90%, and 100% twice), followed by drying with a critical point dryer. Finally, the bacterial cells were sputter-coated with platinum and analyzed via SEM (Hitachi-S4800) at a working voltage of 15.0 kV and working current of 10 µA under 40 K magnification 17.

The internal structures of *S. aureus*, *E. coli*, and *S. pullorum* incubated with Fe^2+^ (50 µM) were observed by TEM. Cells were fixed with 2.5% (vol/vol) glutaraldehyde with phosphate buffer (PB) (0.1 M, pH 7.4) and washed four times in PB. The cells were then postfixed with 1% (wt/vol) osmium tetroxide in PB for 2 h at 4 °C and dehydrated through a graded ethanol series (30%, 50%, 70%, 80%, 90%, 100%, and 100%, 7 min each) in pure acetone (2 × 10 min). Samples were infiltrated in graded mixtures (3:1, 1:1, and 1:3) of acetone and SPI-PON812 resin (16.2 g of SPI-PON812, 10 g of dodecyl succinic anhydride (DDSA), and 8.9 g of N-methylolacrylamide (NMA). Finally, the cells were embedded in pure resin with 1.5% benzyldimethylamine (BDMA) and polymerized for 12 h at 45 °C and 48 h at 60 °C. Ultrathin sections (70 nm thick) were produced using a microtome (Leica EM UC6), double-stained using uranyl acetate and lead citrate, and examined using TEM (FEI Tecnai Spirit 120 kV).

### Lipid ROS measurement

Lipid ROS levels in *S. aureus*, *E. coli*, and* S. pullorum* stimulated by ferrous iron were detected using a BODIPY 581/591 C11 fluorescent probe (Invitrogen). Single colonies of *S. aureus*, *E. coli*, and* S. pullorum* on LB solid agar plates were randomly selected and inoculated in 5 mL of LB liquid medium and cultured at 37 °C for 12 h under 180 rpm rotation. The next day, 50 µL of the bacterial inoculation was diluted with 4 950 µL of fresh LB medium and cultured at 37 °C for 3 h at 180 rpm until OD600 reached 0.8. The bacterial precipitate (1 mL) was obtained by centrifugation at 3 600 rpm for 5 min at 4 °C and diluted with H_2_O (900 μL). Then, 100 μL of Fe^2+^ (25, 50, 100, and 200 µM) was mixed with *S. aureus* (900 μL), *E. coli* (900 μL), and *S. pullorum* (900 μL) at 37 °C for 30 min, respectively. The bacterial precipitate was obtained by centrifugation at 3 600 rpm for 5 min at 4 °C, after which 200 µL of BODIPY 581/591 C11 probe (2 µM) working solution was added to the bacteria for 30 min of incubation at 37 °C. The lipid ROS level was measured by a multi-scan spectrum with excitation at 488 nm and emission at 525 nm.

### Bacterial membrane potential measurement

Changes in bacterial membrane potential following ferrous iron treatment were detected using a DIOC_2_(3) probe. The *S. aureus*, *E. coli*, and* S. pullorum* precipitates (1 mL) at the logarithmic growth stage were obtained by centrifugation at 3 600 rpm for 5 min at 4 °C and diluted with H_2_O (900 μL). Then, 100 μL of Fe^2+^ (25, 50, 100, and 200 µM) was mixed with *S. aureus* (900 μL), *E. coli* (900 μL), and *S. pullorum* (900 μL) at 37 °C for 30 min, respectively. The bacterial precipitate was obtained by centrifugation at 3 600 rpm for 5 min at 4 °C, after which 100 µL of 30 µM DIOC_2_(3) (US EVERBRIGHT) probe working solution was added to the bacteria for 30 min of incubation at 37 °C. Bacterial membrane potential was measured using a multi-scan spectrum with Ex/Em = 480/525 nm (green fluorescence) and Ex/Em = 530/590 nm (red fluorescence). Red/green fluorescence ratios were calculated using population mean fluorescence intensities for bacteria.

### Lactate dehydrogenase (LDH) measurement

Changes in bacterial membrane integrity following ferrous iron treatment were detected using an LDH assay kit (Solarbio). The *S. aureus* and *E. coli* precipitates (1 mL) at the logarithmic growth stage were obtained by centrifugation at 3 600 rpm for 5 min and diluted with H_2_O (450 μL). Then, 50 μL of Fe^2+^ (25, 50, and 200 µM) was mixed with *S. aureus* (450 μL) and *E. coli* (450 μL) at 37 °C for 30 min, respectively. The supernatant was collected by centrifugation at 3 600 rpm for 5 min at 4 °C. The LDH activity in the supernatant was then measured using an LDH assay kit (Solarbio).

### Bacterial live/dead staining assays

The *S. aureus* and *E. coli* precipitates (1 mL) at the logarithmic growth stage were obtained by centrifugation at 3 600 rpm for 5 min at 4 °C and diluted with H_2_O (900 μL). Then, 100 μL of Fe^2+^ (25, 50, 100, and 200 µM) was mixed with *S. aureus* (900 μL) and *E. coli* (900 μL) at 37 °C for 30 min, respectively. The bacterial precipitate was obtained by centrifugation at 3 600 rpm for 5 min at 4 °C, after which 1 mL of 5 µM SYTO 9 (Invitrogen) working solution was added to the bacteria for 30 min of incubation at 37 °C. After washing twice with PBS, 1 mL of 10 µM propidium iodide (PI) (Beyotime Biotechnology) working solution was added to the bacteria for 30 min of incubation at 37 °C. After washing twice with PBS, DAPI (Beyotime Biotechnology) was used to stain the nuclei. After washing, live and dead bacteria after ferrous treatment were observed using a Zeiss confocal microscope (Zeiss LSM980).

### Isolation and culture of bone marrow-derived macrophages (BMMs)

Six-week-old male Balb/C mice (obtained from the Laboratory of Immunodeficient Animals, Institute of Biophysics, Chinese Academy of Sciences) were sacrificed by cervical dislocation, immersed in 75% alcohol for 3 min, and then soaked in PBS containing 2% penicillin/streptomycin (Sangon Biotech) for 3 min. The femur and tibia were taken aseptically and immersed in 10 mL of RPMI 1640 medium containing 2% penicillin/streptomycin. Bone marrow was flushed into a sterile 15-mL centrifuge tube with a 1-mL syringe drawn from the 1640 medium and then centrifuged at 1 500 rpm for 10 min at 4 °C. The supernatant was discarded and 5 mL of fresh 1640 medium was added to resuspend the cells, followed by centrifugation at 1 500 rpm for 10 min at 4 °C. The supernatant was discarded, and the cells were resuspended in 10 mL of complete culture medium (RPMI 1640 + 20% FBS + 1% penicillin/streptomycin + 20 ng/mL recombinant mouse granulocyte macrophage colony stimulating factor (rmGM-CSF)), inoculated into cell culture dishes (100 mm), and incubated in an incubator at 37 °C with 5% CO_2_ for 3 d, with the culture medium changed. The cells were continuously cultured until day 5, with all semi-adherent cells, i.e., enriched mouse BMMs, then collected. The BMMs were labelled with a FITC-CD 11b fluorescent probe (Invitrogen) for 30 min at 4 °C and the purity of the isolated cells was assayed by flow cytometry (BD FACSCalibur).

### Detection of intracellular bactericidal effects of ferroptosis inducers and inhibitors

RAW264.7 cells were inoculated in 6-well plates containing 1 mL of complete culture solution (RPMI 1640 + 20% FBS + 1% penicillin/streptomycin). At the logarithmic growth phase, the RAW264.7 cells were infected with *S. aureus*, *E. coli*, *S. pullorum*, and *Salmonella enteritidis* (SC070) (*S. enteritidis*) at MOI = 20:1 for 1 h, respectively, and extracellular bacteria were killed with gentamicin (200 μg/mL) for 3 h. After washing three times with PBS, the cells were cultured at 37 °C under 5% CO_2_ in 900 µL of RPMI 1640 containing 1% FBS, with 100 µL of ferroptosis inducers (RSL3 (2, 5, and 10 µM), SSZ (2, 5, and 10 µM), and APAP (2, 5, and 10 µM)) or inhibitors (Fer-1 (2, 5, and 10 µM), EDTA (10, 20, and 50 µM), and DFO (12.5, 25, and 50 µM)) added to the cell culture medium for 6 h (RSL3 incubated for 3 h). A sample of the cells was lysed using 1 mL of buffer containing 0.4% Triton X-100 for 30 min. The number of viable bacteria was counted by plating serial dilutions of bacteria on LB solid medium. Cell activity after bacterial infection and treatment with ferroptosis inducers and inhibitors was assayed with a Cell Counting Kit-8 (CCK-8) (DOJINDO).

RAW264.7 cells were infected with *S. aureus* and* E. coli* at MOI = 20:1 for 1 h, respectively, and extracellular bacteria were killed with gentamicin (200 μg/mL) for 3 h. After washing three times with PBS, the cells were cultured at 37 °C under 5% CO_2_ in 900 µL of RPMI 1640 containing 1% FBS, with 100 µL of ferroptosis inducers (Fe^2+^ (50µM), RSL3 (5 µM), SSZ (10 µM), and APAP (10 µM)) or inhibitors (Fer-1 (10 µM), EDTA (50 µM), and DFO (50 µM)) added to the cell culture medium for 6 h. TEM was used to observe the bacteria in the cells. Host cell morphological images with different treatments were captured using a Zeiss LSM980confocal microscope.

The BMMs were inoculated in 6-well plates containing 1 mL of complete culture solution (RPMI 1640 + 20% FBS + 1% penicillin/streptomycin). At the logarithmic growth phase, RAW264.7 cells were infected with *S. aureus*, *E. coli*, and *S. pullorum* at MOI = 20:1 for 1 h, respectively, and extracellular bacteria were killed with gentamicin (200 μg/mL) for 3 h. After washing three times with PBS, the cells were cultured at 37 °C under 5% CO_2_ in 900 µL of RPMI 1640 containing 1% FBS, with 100 µL of ferroptosis inducers (RSL3 (2, 5, and 10 µM), SSZ (2, 5, and 10 µM), and APAP (2, 5, and 10 µM)) or inhibitors (Fer-1 (0.25, 0.5, and 1 µM) and DFO (12.5, 25, and 50 µM)) added to the cell culture medium at 6 h. A sample of the cells was lysed using 1 mL of buffer containing 0.4% Triton X-100. The number of viable bacteria was counted by plating serial dilutions of bacteria on LB solid medium. Cell activity after bacterial infection and treatment with ferroptosis inducers and inhibitors was assayed with a Cell Counting Kit-8 (CCK-8) (DOJINDO) and Annexin V-FITC Apoptosis Detection Kit (Beyotime Biotechnology).

### Detection of intracellular bactericidal effects of ferrous and ferric iron

RAW264.7 cells were inoculated in 6-well plates containing 1 mL of complete culture solution (RPMI 1640 + 20% FBS + 1% penicillin/streptomycin). At the logarithmic growth phase, RAW264.7 cells were infected with *S. aureus*, *E. coli*, and *S. pullorum* at MOI = 20:1 for 1 h, respectively, and extracellular bacteria were killed with gentamicin (200 μg/mL) for 3 h. After washing three times with PBS, the cells were cultured at 37 °C under 5% CO_2_ in 900 µL of RPMI 1640 containing 1% FBS, with 100 µL of Fe^2+^ (12.5, 25, and 50 µM) or Fe^3+^ (12.5, 25, and 50 µM) then added to the cell culture medium for 6 h. A sample of the cells was lysed using 1 mL of buffer containing 0.4% Triton-X for 30 min. The number of viable bacteria was counted by plating serial dilutions of bacteria on LB solid medium. Intracellular lipid ROS levels were detected using the BODIPY581/591 C11 fluorescent probe after incubation of ferric iron with bacteria-infected macrophages.

### Co-localization of FPN with intracellular bacteria

RAW264.7 cells at the logarithmic growth phase were collected and inoculated into 24-well cell culture plates with round coverslip (Biosharp) placed in advance, followed by the addition of 500 µL of complete culture medium. Cells were then cultured to 80% confluence at 37 °C and 5% CO_2_. Individual colonies of BL21 (*E. coli* strain with green fluorescence) on LB solid plates were randomly selected and inoculated in 5 mL of LB liquid medium containing kanamycin (50 µg/mL), then incubated for 12 h at 37 °C with 180 rpm rotation. The following day, 50 µL of the bacterial inoculation was diluted with 4 950 µL of fresh LB medium containing 50 µg/mL kanamycin and cultured at 37 °C for 3 h under 180 rpm until OD600 reached 0.8. After this, 0.5 mM isopropyl thiogalactoside (IPTG) (GenStar) was added to the bacterial solution to induce 3 h. RAW264.7 cells were infected with BL21 at MOI = 20:1 after IPTG induction for 1 h and extracellular bacteria were killed with gentamicin (200 μg/mL) for 3 h. After washing three times with PBS, the cells were cultured at 37 °C under 5% CO_2_ in 0.5 mL of RPMI 1640 containing 1% FBS for 1, 3, 6, 12, and 24 h. The cells were then rinsed three times with PBS, fixed with 4% paraformaldehyde for 30 min, saturated with 0.4% Triton X-100 for 5 min, and blocked with 5% albumin bovine V (BSA) for 1 h. Cells were then incubated at 4 °C for 12 h with 200 μL of FPN primary antibodies (1:200; Thermo Fisher), followed by incubation with 200 μL of fluorescein Alexa Fluor 647-conjugated goat anti-rabbit IgG (1:200; BIORIGIN) at 37 °C for 1.5 h, and staining with DAPI (Beyotime Biotechnology) for 5 min. After washing, the co-localization of FPN with BL21 was observed using a Zeiss confocal microscope (Zeiss LSM980).

### Co-localization of FPN with bacteria by immunoelectron microscopy and colloidal gold labeling

RAW264.7 cells were infected with *S. aureus* and *E. coli* at MOI = 20:1 for 1 h, respectively, and extracellular bacteria were killed with gentamicin (200 μg/mL) for 3 h. After washing three times with PBS, the cells were cultured at 37 °C under 5% CO_2_ in 1 mL of RPMI 1640 containing 1% FBS for 3 h. Co-localization of FPN with bacteria was observed by immunoelectron microscopic colloidal gold labeling. Cells were fixed with 0.1% (vol/vol) glutaraldehyde + 4% paraformaldehyde in PB (0.1 M, pH 7.4) and washed four times in PB. The cell pellet was suspended in 12% wt/vol gelatin in PBS at 37 °C for 30 min, then placed on ice for 10-20 min to solidify the gelatin. The cell pellet was cut into small blocks and placed in 2.3 M sucrose overnight at 4 °C. The specimen blocks were then removed from the sucrose to aluminum specimen holders, which were frozen by immersion in liquid nitrogen. Ultrathin sections were cut at -120 °C using an ultramicrotome (Leica EM FC7, Germany), and collected on grids. The grids were washed with PBS, incubated with 1% BSA for 30 min, then with anti-FPN (Thermo Fisher) primary antibodies (1:20) at 37 °C for 2 h. After washing with PBS, the grids were incubated the IgG-conjugated-gold (GAR IgG 10 nm, Aurion) secondary antibodies (1:20) for 1 h at room temperature. The labeled samples were placed on uranyl acetate/methyl cellulose (UA/MC, 1:9) drops for 5 min, then picked up by the loop and allowed to dry. The samples were then observed by TEM (FEI Tecnai Spirit, USA) at 100 kV.

### Co-localization of ferrous iron with intracellular bacteria

RAW264.7 cells at the logarithmic growth phase were collected and inoculated into 24-well cell culture plates with round coverslip (Biosharp) placed in advance, followed by the addition of 500 µL of complete culture medium. Cells were then cultured to 80% confluence at 37 °C and 5% CO_2_. Individual colonies of BL21 (with green fluorescence) on LB solid plates were randomly selected and inoculated in 5 mL of LB liquid medium containing kanamycin (50 µg/mL) and incubated for 12 h at 37 °C with 180 rpm rotation. The following day, 50 µL of the bacterial inoculation was diluted with 4 950 µL of fresh LB medium containing 50 µg/mL kanamycin and cultured at 37 °C for 3 h under 180 rpm until OD600 reached 0.8. Then, 0.5 mM IPTG (GenStar) was added to the bacterial solution for induction for 3 h. The bacterial precipitate was obtained by centrifugation at 3 600 rpm for 5 min at 4 °C and diluted to 1 × 10^8^ CFUs/mL with 5 mL of RPMI 1640 culture medium. The RAW264.7 cells were infected with 50 µL of BL21 bacterial solution at MOI = 20:1 for 1 h, and extracellular bacteria were killed with gentamicin (200 μg/mL) for 3 h. After washing three times with PBS, the cells were cultured at 37 °C under 5% CO_2_ in 0.5 mL of RPMI 1640 containing 1% FBS for 6, 12, and 24 h. The cells were then washed three times with PBS. Intracellular ferrous iron was labelled with a FeRhoNox^TM^-1 fluorescent probe (GORYO Chemical) at 37 °C for 45 min. DAPI was used to stain the nuclei. After washing, the co-localization of ferrous iron with BL21 was observed using a Zeiss confocal microscope (Zeiss LSM980).

### Co-localization of lipid ROS with intracellular bacteria

RAW264.7 cells at the logarithmic growth phase were collected and inoculated into 24-well cell culture plates with round coverslip (Biosharp) placed in advance, followed by the addition of 500 µL of complete culture medium. Cells were then cultured to 80% confluence at 37 °C and 5% CO_2_. Individual colonies of BL21 (with mCherry fluorescence) on LB solid plates were randomly selected and inoculated in 5 mL of LB liquid medium containing ampicillin (50 µg/mL) and incubated for 12 h at 37 °C with 180 rpm rotation. The following day, 50 µL of the bacterial inoculation was diluted with 4 950 µL of fresh LB medium containing 50 µg/mL ampicillin and cultured at 37 °C for 3 h under 180 rpm until OD600 reached 0.8. The bacterial precipitate was obtained by centrifugation at 3 600 rpm for 5 min at 4 °C and diluted to 1 × 10^8^ CFUs/mL with 5 mL of RPMI 1640 culture medium. The RAW264.7 cells were infected with 50 µL of BL21 bacterial solution at MOI = 20:1 for 1 h, and extracellular bacteria were killed with gentamicin (200 μg/mL) for 3 h. After washing three times with PBS, the cells were cultured at 37 °C under 5% CO_2_ in 0.5 mL of RPMI 1640 containing 1% FBS for 6, 12, and 24 h. Cells were washed three times with PBS. Lipid ROS were stained with 100 µL of Liperfluo (10 µM) at 37 °C for 30 min. DAPI was used to stain the nuclei. After washing, the co-localization of lipid ROS with BL21 was observed using a Zeiss confocal microscope (Zeiss LSM980).

### Ferritin and transferrin receptor fluorescence intensity measurement

RAW264.7 cells were inoculated in 24-well cell culture plates with round coverslip placed in advance. At the logarithmic growth phase, the RAW264.7 cells were infected with *E. coli* at MOI = 20:1 for 1 h, and extracellular bacteria were killed with gentamicin (200 μg/mL) for 3 h. After washing three times with PBS, the cells were cultured at 37 °C under 5% CO2 in 0.5 mL of RPMI 1640 containing 1% FBS for 1, 3, 6, 12, and 24 h. Cells were washed three times with PBS, fixed with 4% paraformaldehyde for 30 min, saturated with 0.4% Triton X-100 for 5 min, and blocked with 5% albumin bovine V (BSA) for 1 h. Cells were then incubated at 4 °C for 12 h with 200 μL of ferritin or transferrin receptor primary antibodies (1:200; Abcam), followed by incubation with 200 μL of fluorescein Alexa Fluor 488-conjugated goat anti-rabbit IgG (1:200; BIORIGIN) at 37 °C for 1.5 h, and staining with DAPI (Beyotime Biotechnology) for 5 min. After washing, the fluorescence intensity of ferritin and transferrin receptor was observed using a Zeiss confocal microscope (Zeiss LSM980).

### Intracellular bactericidal assay of RAW264.7 macrophages after FPN knockdown

Ferroportin shRNA (MSH031547-LVRU6P-b/c, Matrix) and null shRNA (CSHC7R001-1-LVRU6P, Matrix) were transfected into 293T cells. The viral supernatant was collected 48 h after transfection and filtered through a 0.22-μM filter. The RAW264.7 macrophages were inoculated in 6-well plates containing 1 mL of complete culture solution (RPMI 1640 + 20% FBS + 1% penicillin/streptomycin) and infected with 1 mL of the filtered virus for 2 h, then replenished with 2 mL of fresh RPMI 1640 culture medium (RPMI 1640 + 1% FBS + 1% penicillin/streptomycin) and incubated for 24 h at 37 °C. The medium was replaced with fresh RPMI 1640 culture medium (RPMI 1640 + 1% FBS + 1% penicillin/streptomycin), followed by an additional 48 h of culture at 37 °C. After 48 h of virus infection, 2 mL of 2.5 µg/mL puromycin (GenStar) was added to the culture medium for screening. Fresh culture medium was replaced every 2 days and the cells were incubated continuously for more than a week at 37 °C under 5% CO_2_. After washing with ice-cold PBS, the cells were collected by centrifugation at 2 000 rpm for 5 min at 4 °C and lysed in 100 µL of RIPA buffer (Beyotime Biotechnology) containing protease inhibitor. The lysate was centrifuged at 12 000 g for 10 min at 4 °C and the protein concentrations were determined and calibrated using a BCA Protein Assay Kit (Thermo Fisher). Protein extracts were resolved on sodium dodecyl sulfate (SDS)-polyacrylamide gels, transferred to polyvinylidene fluoride (PVDF) membranes, and blocked for 1 h with Tris-buffered saline (TBS) containing 0.05% Tween 20 (TBST) and 5% skimmed milk powder. The PVDF membranes were washed three times with TBST (10 min each time). Ferroportin antibodies (Thermo Fisher) were diluted 1:1 000 in TBST (containing 5% bovine serum albumin) and incubated overnight at 4 °C. After washing with TBST, membranes were incubated with horseradish peroxidase (HRP)-conjugated goat anti-rabbit antibodies (Sigma) in a solution of TBST. Protein bands were visualized using a fully automated chemiluminescence image analysis system (Tanon-4600SF).

The RAW264.7 cells were inoculated into a 6-well plate containing 1 mL of complete culture medium (RPMI 1640 + 20% FBS + 1% penicillin/streptomycin). The RAW264.7 cells were infected with *S. aureus*, *E. coli*, and* S. pullorum* at MOI = 20:1 after FPN knockdown for 1 h, respectively, and extracellular bacteria were killed with gentamicin (200 μg/mL) for 3 h. After washing three times with PBS, the cells were cultured at 37 °C under 5% CO_2_ in 1 mL of RPMI 1640 containing 1% FBS for 3 h. A sample of the cells was lysed using buffer containing 0.4% Triton X-100 for 30 min. The number of viable bacteria was counted by plating serial dilutions of bacteria on LB solid medium.

### Hepcidin bactericidal assay for intracellular bacteria

Hepcidin (100 µL; 2, 4, and 6 µg/mL) was added to RAW264.7 cells cultured in 6-well plates for 3 h of incubation at 37 °C. The pre-treated RAW264.7 cells were then infected with *S. aureus*, *E. coli*, and *S. pullorum* at MOI = 20:1 for 1 h, respectively, and extracellular bacteria were killed with gentamicin (200 μg/mL) for 3 h. After washing three times with PBS, the cells were cultured at 37 °C under 5% CO_2_ in 1 mL of RPMI 1640 containing 1% FBS for 3 h. RAW264.7 cells cultured in 6-well plates were infected with *S. aureus*, *E. coli*, and *S. pullorum* at MOI = 20:1 for 1 h, respectively, and extracellular bacteria were killed with gentamicin (200 μg/mL) for 3 h. After washing three times with PBS, the cells were cultured at 37 °C under 5% CO_2_ in 1 mL of RPMI 1640 containing 1% FBS and hepcidin (2, 4, and 6 μg/mL) for 3 h. A sample of the cells was lysed using buffer containing 0.4% Triton X-100 for 30 min. The number of viable bacteria was counted by plating serial dilutions of bacteria on LB solid medium. Immunoblot assay was used to detect the effect of hepcidin on FPN. Intracellular ferrous iron was detected with a FeRhoNox^TM^-1 fluorescent probe (GORYO Chemical), with 200 µL of 5 µM FeRhoNox^TM^-1 working solution then added to the cells for 30 min of incubation at 37 °C. The cells were detected by flow cytometry (BD FACSCalibur).

### Western blot

RAW264.7 cells cultured in 6-well plates were infected with *S. aureus*, *E. coli*, and *S. pullorum* infected at MOI = 20:1 for 1 h, respectively, and extracellular bacteria were killed with gentamicin (200 μg/mL) for 1, 3, 6, 12, and 24 h. After washing with ice-cold PBS, the cells were lysed in RIPA buffer (Beyotime Biotechnology) containing protease inhibitor. The lysate was centrifuged at 12 000 g for 10 min at 4 °C and the protein concentrations were determined and calibrated using a BCA Protein Assay Kit. Protein extracts were resolved on SDS-polyacrylamide gels, transferred to PVDF membranes, and blocked for 1 h with 0.05% TBST and 5% skimmed milk powder. The PVDF membranes were washed three times with TBST (10 min each time). Primary antibodies were diluted 1:1 000 in TBST (containing 5% BSA) and incubated overnight at 4 °C. The following primary antibodies were used: anti-HO-1 (Abcam), anti-FPN (Thermo Fisher), anti-Ferritin (Abcam), anti-NCOA-4 (Abcam), anti-Nrf2 (Abcam), anti-GPX4 (Abcam), and anti-GAPDH (Abcam). After washing with TBST, the membranes were incubated with HRP-conjugated goat anti-rabbit (Abcam) or anti-mouse (Abcam) secondary antibodies (1:1000) in TBST for 1 h at room temperature. Protein bands were visualized using a fully automated chemiluminescence image analysis system (Tanon-4600SF).

RAW264.7 cells were inoculated into a 6-well plate containing 1 mL of complete culture medium (RPMI 1640 + 20% FBS + 1% penicillin/streptomycin). Cells were then cultured at 37 °C under 5% CO_2_ to 80%-90% confluence, thus achieving a cell count of 1×10^6^. The culture was then replaced with 1 mL of RPMI 1640 containing 1%, 2.5%, and 20% FBS and incubated for 24 h at 37 °C. After washing with ice-cold PBS, the cells were lysed in RIPA buffer (Beyotime Biotechnology) containing protease inhibitor. The lysate was centrifuged at 12 000 g for 10 min at 4 °C and the protein concentrations were determined and calibrated using a BCA Protein Assay Kit. Protein extracts were resolved on SDS-polyacrylamide gels, transferred to PVDF membranes, and blocked for 1 h with 0.05% TBST and 5% skimmed milk powder. The PVDF membranes were washed three times with TBST (10 min each time). The LC3 primary antibodies (Abcam) were diluted 1:1 000 in TBST (containing 5% BSA) and incubated overnight at 4 °C. After washing with TBST, the membranes were incubated with HRP-conjugated goat anti-rabbit (Abcam) secondary antibodies (1:1000) in TBST for 1 h at room temperature. Protein bands were visualized using a fully automated chemiluminescence image analysis system (Tanon-4600SF).

### *In vivo* planktonic tail vein infection

Six-week-old male Balb/C mice (obtained from the Laboratory of Immunodeficient Animals, Institute of Biophysics, Chinese Academy of Sciences) were used to establish *in vivo* infection models. The mice (five in each group) were infected in the tail vein with *S. aureus* (1 × 10^6^ CFUs) or *S. pullorum* (1 × 10^6^ CFUs) at the logarithmic growth stage. After 12 h of infection, blood was collected from the orbital vein and erythrocytes were lysed in erythrocyte lysate (1 mL) (TIANGEN) at room temperature. The mixture was centrifuged at 3 500 rpm for 10 min at 4 °C to remove the erythrocyte lysate. The cells were then divided equally into two portions. One portion of cells was labelled with 200 µL of BODIPY 581/591 C11 (2 µM), followed by incubation for 30 min at 37 °C. After washing with PBS, 100 µL of APC CD 11b antibody (Invitrogen) working solution (1:1 000) was added to the cells, followed by 30 min of incubation at 4 °C. The cells were then washed three times with PBS, and changes in lipid ROS in peripheral blood macrophages were detected by flow cytometry.

The other cells were labelled with 200 µL of FeRhoNox^TM^-1 probes (5 µM), followed by incubation for 45 min at 37 °C. After washing with PBS, 100 µL of APC CD 11b antibody (Invitrogen) working solution (1:1 000) was added to the cells, followed by 30 min of incubation at 4 °C. The cells were then washed three times with PBS, and changes in ferrous iron in peripheral blood macrophages were detected by flow cytometry.

After 12 h of infection, 100 μL of SSZ (10, 50, and 100 µM) or DFO (10, 50, and 100 µM) was administered intravenously twice a day, with a total of five treatments per mouse. Control mice with bacterial infection received 100 μL of PBS only via intravenous injection. Body weight and behavior, including feeding, drinking, and activity, were continuously observed and recorded for 3 days. All mice were sacrificed on day 3 after infection, and their hearts, livers, lungs, spleens, and kidneys were collected in 5 mL of PBS. Tissue samples were ground under aseptic conditions. Viability of bacteria was assessed by calculating bacterial number (CFUs/mouse) in organs. Antibacterial efficiency was determined by calculating the ratio of bacterial number between the SSZ or DFO-treated groups and PBS-treated group.

After 12 h of infection, 100 μL of SSZ (100 µM) or DFO (100 µM) was administered intravenously twice a day, with a total of five treatments per mouse. Control mice with bacterial infection received 100 μL of PBS only via intravenous injection. All mice were sacrificed on day 3 after infection. Their main organs (i.e., liver, kidneys, spleen, lungs, and heart) were collected in 10 mL of 4% paraformaldehyde, then fixed in paraffin and stained with hematoxylin-eosin (H&E) to examine changes in organ histomorphology.

After 12 h of infection, 100 μL of SSZ (100 µM) or DFO (100 µM) was administered intravenously twice a day, with a total of five treatments per mouse. Control mice with bacterial infection received 100 μL of PBS only via intravenous injection. All mice were sacrificed on day 3 after infection, and blood was collected from the orbital vein and erythrocytes were lysed in erythrocyte lysate (1 mL) (TIANGEN) at room temperature. The mixture was centrifuged at 3 500 rpm for 10 min at 4 °C to remove the erythrocyte lysate. The cells were then divided equally into two portions. One portion of cells was labelled with 200 µL of BODIPY 581/591 C11 (2 µM), followed by incubation for 30 min at 37 °C. After washing with PBS, 100 µL of APC CD 11b antibody (Invitrogen) working solution (1:1 000) was added to the cells, followed by 30 min of incubation at 4 °C. The cells were then washed three times with PBS, and changes in lipid ROS in peripheral blood macrophages were detected by flow cytometry. The other cells were labelled with 200 µL of FeRhoNox^TM^-1 probes (5 µM), followed by incubation for 45 min at 37 °C. After washing with PBS, 100 µL of APC CD 11b antibody (Invitrogen) working solution (1:1 000) was added to the cells, followed by 30 min of incubation at 4 °C. The cells were then washed three times with PBS, and changes in ferrous iron in peripheral blood macrophages were detected by flow cytometry.

### Evaluation of antibacterial efficacy of SSZ and DFO with intravenous intracellular bacterial infections

Six-week-old male Balb/C mice (obtained from the Laboratory of Immunodeficient Animals, Institute of Biophysics, Chinese Academy of Sciences) were used to establish *in vivo* infection models. Mouse BMMs were infected with* S. aureus* or *S. pullorum* following the *in vitro* intracellular bacterial model. Following infection, cells were collected from a single well of a 6-well plate, before being resuspended in 1 mL of PBS, after which 100 μL (10^5^ cells) of the mixture was intravenously injected into each mouse. After 12 h of infection, 100 μL of SSZ (100 µM) or DFO (100 µM) was administered intravenously twice a day, with a total of five treatments per mouse. Control mice with bacterial infection received 100 μL of PBS only via intravenous injection. Body weight and behavior, including feeding, drinking, and activity, were continuously observed and recorded for 3 days. All mice were sacrificed on day 3 after infection, and kidneys were collected in 5 mL of PBS. Tissue samples were ground under aseptic conditions. Viability of bacteria was assessed by calculating bacterial number (CFUs/mouse) in the two kidneys. Antibacterial efficiency was determined by calculating the ratio of bacterial number between the SSZ or DFO-treated groups and PBS-treated group.

### Biosafety evaluation of SSZ or DFO

Six-week-old male Balb/C mice were used to establish *in vivo* infection models. Fifteen healthy Balb/C mice were randomly divided into three groups (five in each group). Two groups were intravenously injected with 100 μL of SSZ (100 µM) and DFO (100 µM) twice a day, respectively. One group was intravenously injected with PBS as the control. On day 3, the injections were stopped, and the mice were housed until day 7. Body weight and behavior, including feeding, drinking, and activity, were continuously observed and recorded for 7 days. All mice were sacrificed on day 7 after infection and blood was collected from the orbital vein to assess the levels of hemoglobin (Hb), mean corpuscular hemoglobin (MCH), mean corpuscular volume (MCV), red blood cells (RBC), hematocrit (HCT), white blood cells (WBC), platelet count (PLT), neutrophilic granulocytes (NEUT). The main organs, i.e., liver, kidneys, spleen, lungs, and heart, were collected in 10 mL of 4% paraformaldehyde, then fixed in paraffin and stained with H&E to examine changes in organ histomorphology.

### Statistical analysis and reproducibility

All experiments were performed as biological replicates. Sample size for each experimental group per condition is reported in the corresponding figure legends and Methods section. For bacterial and cell experiments, sample size was not predetermined, and all samples were included in the analysis. In the animal experiments, no statistical methods were used to predetermine sample size (n = number of mice per group), and all animals were used for analysis unless they died. GraphPad Prism v7.0 (GraphPad Software) was used for statistical analyses. For comparing two groups, we used two-tailed Student's* t*-test for column analyses. For comparing multiple groups, we used two-way analysis of variance (ANOVA). NS, not significant (*P* > 0.05); *P < 0.05; **P < 0.01; ***P < 0.001, and ****P < 0.0001.

## Results

### Transient fluctuation in ferroptotic hallmarks in macrophages upon bacterial infection

**Figure 1:**
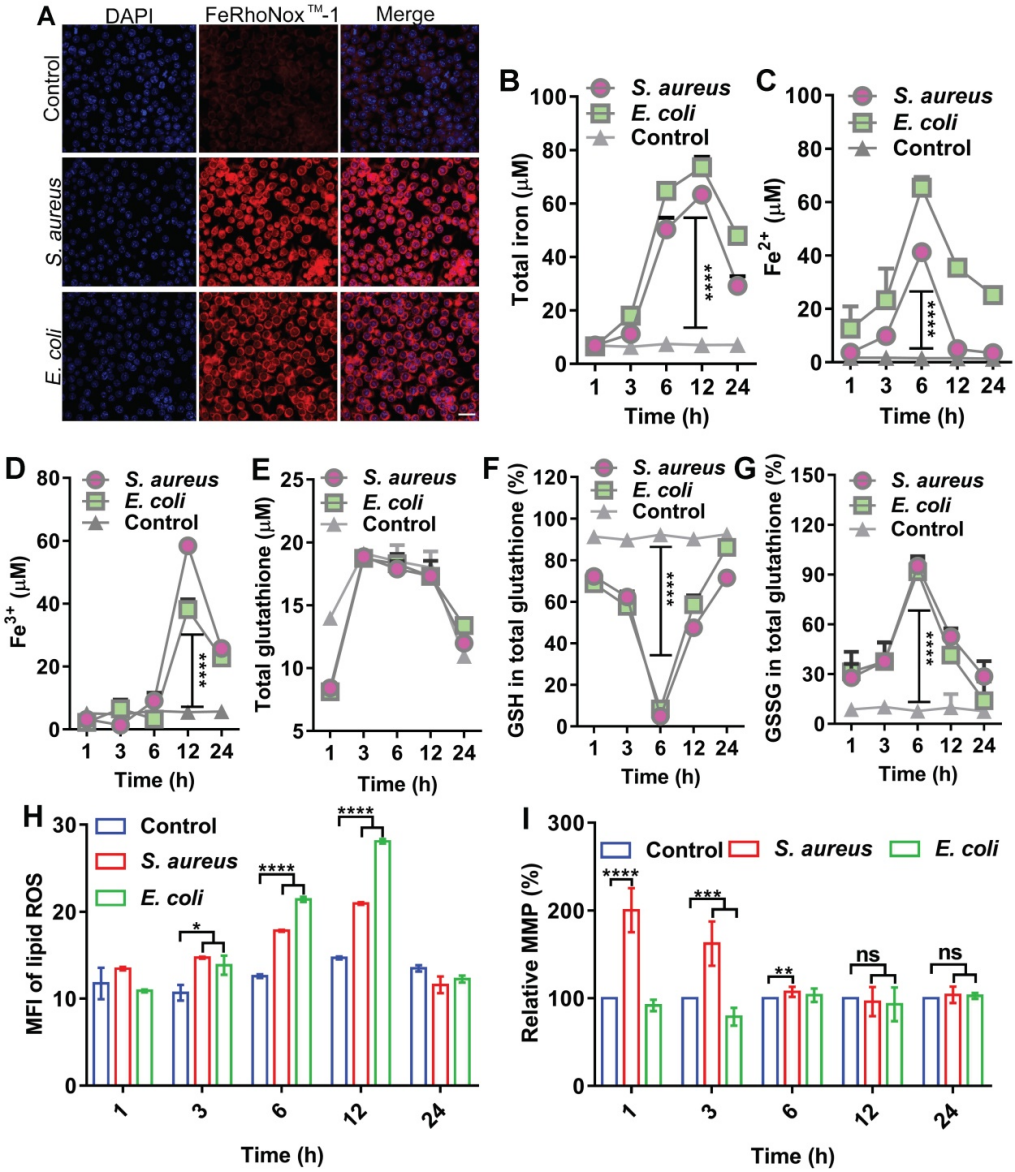
**Ferroptotic stress emerges in macrophages after bacterial infection. (A)** Increase in ferrous iron in macrophages infected by *E. coli* or *S. aureus* at 6 h. FeRhoNox^TM^-1 probe-stained ferrous iron. DAPI-stained cell nucleus. Scale bar = 20 µm. **(B-D)** Changes in intracellular iron level after bacterial infection in macrophages. **(E-G)** GSH balance in cells infected by bacteria in macrophages. **(H)** Lipid peroxide level in macrophages infected by bacteria. BODIPY 581/591-C11 probe was used to detect lipid ROS. **(I)** Changes in MMP in macrophages after bacterial invasion. n = 3, *p < 0.05, **p < 0.01, ***p < 0.001, ****p < 0.0001.

In host defense, oxidative stress is often evoked to kill invading bacteria along with substantial changes in biochemical effectors, such as iron, ROS, and GSH. Here, we analyzed the dynamic levels of ferrous and ferric iron during bacterial invasion in macrophages. To establish an intracellular infection model, RAW264.7 cells were infected with *S. aureus* or *E. coli*, then treated with gentamycin (200 µg/mL) for 3 h to kill the extracellular bacteria (Figure S1A-B), while retaining viable *S. aureus* and *E. coli* inside the cell (Figure S1C). RPMI 1640 culture medium containing 1% FBS, which did not induce cell autophagy (Figure S2), was added and then incubated for 6 h. A FeRhoNox^TM^-1 fluorescent probe was used to label intracellular ferrous iron. The changes in intracellular ferrous iron fluorescence intensity were observed using LSM980 laser confocal microscopy and flow cytometry. Confocal images showed that intracellular ferrous fluorescence intensity and ferrous content increased after bacterial infection in the cells (Figure 1A). Flow cytometry showed a gradual increase in ferrous iron level from 1 to 12 h post-bacterial infection in the macrophages and a gradual recovery of ferrous iron content from 12 to 24 h (Figure S3). Measurement of intracellular iron using a serum iron assay kit showed that total, ferrous, and ferric iron levels increased after bacterial infection in the macrophages and exhibited dynamic changes with duration of infection. Total intracellular iron content gradually increased from 1 to 12 h after bacterial infection in the macrophages and then gradually recovered from 12 to 24 h. Intracellular ferrous iron content gradually increased between 1 and 6 h after bacterial infection, while ferric iron content did not change significantly. In contrast, ferrous iron content gradually decreased and ferric iron content increased between 6 and 24 h after bacterial infection. This indicates that ferrous iron was oxidized to ferric iron (Figure 1B-D). A GSH and GSSG Assay Kit was used to detect GSH/GSSG balance in the cells infected by bacteria. Results showed no significant change in total intracellular GSH levels after bacterial infection in the macrophages. In contrast, the intracellular level of GSH gradually decreased and oxidized GSSG gradually increased from 1 to 6 h after bacterial infection. Subsequently, the intracellular level of GSH gradually increased and oxidized GSSG gradually decreased from 6 to 24 h. Thus, the intracellular redox level was altered after bacterial infection in the macrophages (Figure 1E-G). The BODIPY 581/591 C11 fluorescent probe revealed a gradual increase in intracellular lipid ROS content from 1 to12 h after bacterial infection in macrophages and gradual recovery of lipid ROS content from 12 to 24 h (Figure 1H, Figure S4). Furthermore, the JC-1 fluorescent probe revealed that MMP was altered between 1 and 6 h after bacterial infection in the macrophages but recovered to a normal state between 12 and 24 h (Figure 1I). Results also showed that although intracellular lipid ROS levels increased after bacterial infection in RAW264.7 cells, mitochondria were not damaged. In summary, bacterial infection in the macrophages resulted in the elevation in intracellular ferrous iron content, alteration of redox levels, and accumulation of lipid ROS, indicating the occurrence of ferroptosis in cells. However, these changes did not last long-term or lead to cell death, with all cellular indicators gradually returning to normal in the later stages of infection, thus ensuring cellular activity. In summary, these characteristics suggest the existence of a strong correlation between ferroptosis and macrophage killing of bacteria, which could be attributed to ferrous iron. We hypothesize that the presence of ferrous iron may facilitate host control of invasive bacteria.

### Ferroptosis stress contributes to macrophage defense against intracellular bacteria

**Figure 2:**
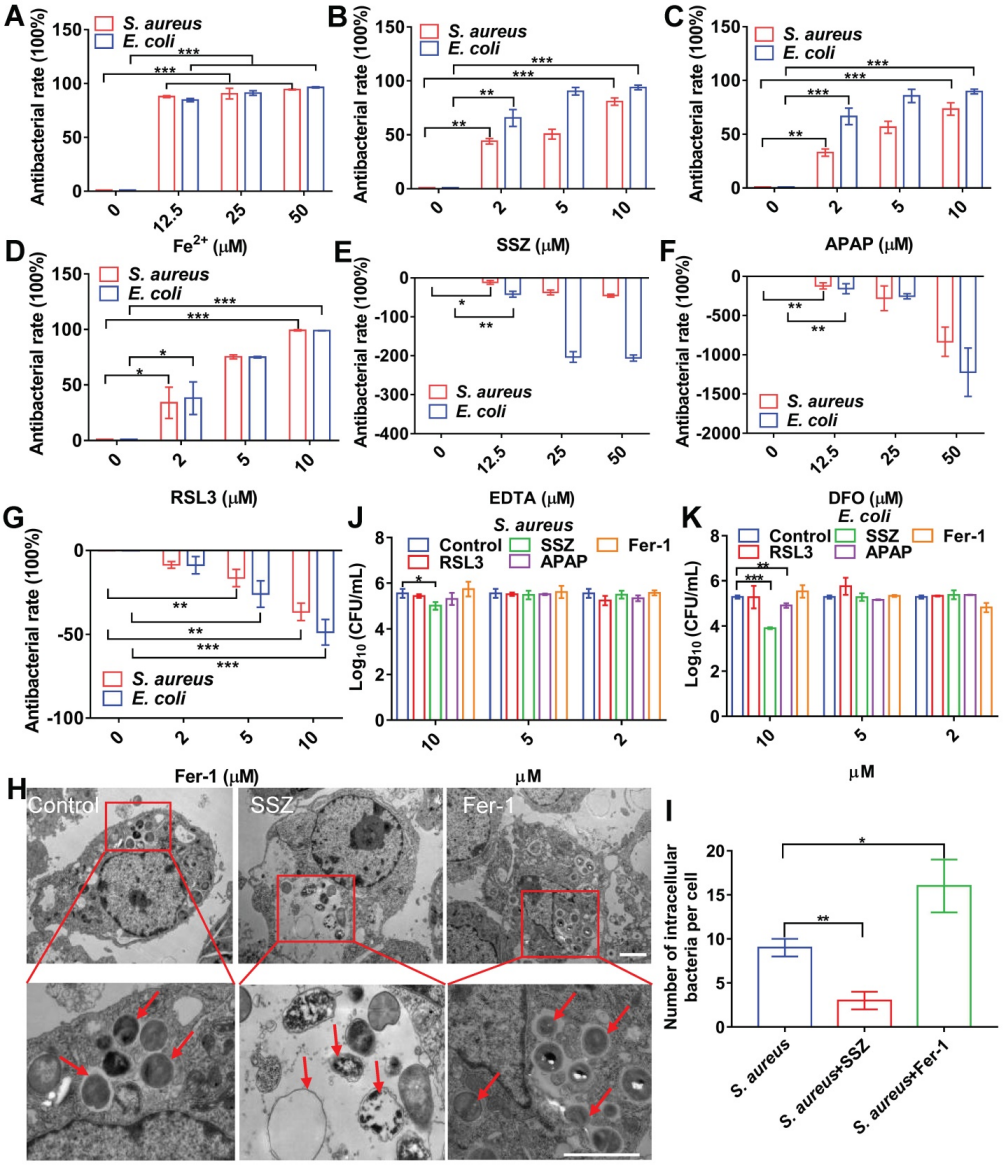
** Cellular ferroptotic stress contributes to macrophage defense against intracellular bacteria. (A)** Inhibitory effects of ferrous iron on intracellular bacteria. **(B-D)** Antibacterial effects of ferroptosis inducers on intracellular bacteria.** (E-G)** Effects of ferroptosis inhibitors on intracellular bacteria. **(H-I)** TEM characterization of intracellular *S. aureus* treated with SSZ or Fer-1. Scale bar = 2 µm.** (J-K)** Effects of ferroptosis inducers and inhibitors on planktonic bacteria. n = 3, *p < 0.05, **p < 0.01, ***p < 0.001.

To confirm the association between cellular ferroptosis and intracellular bacteria, we characterized the viability of intracellular bacteria with ferroptosis inducers and inhibitors. Bacterial infection in RAW264.7 cells resulted in increased intracellular levels of ferrous iron and lipid ROS, which were coincident hallmarks of bacterial death. We first evaluated the effects of adding ferrous iron to infected macrophages. To avoid cytotoxicity to macrophages, concentration and treatment time of the ferrous iron were optimized (Figure S6A). RAW264.7 cells were infected with *S. aureus*, *E. coli*, *S. pullorum*, or *S. enteritidis*, then treated with gentamycin to kill extracellular bacteria while retaining viable bacteria inside the cell. The viability of intracellular *S. aureus*, *E. coli*, *S. pullorum*, and *S. enteritidis* was assessed by counting CFUs when ferrous iron was added for 6 h of incubation at 37 °C. Compared to the control, ferrous iron (25 µM) inhibited more than 90% of *S. aureus*, 90% of *E. coli*, 50% of *S. pullorum*, and 60% of* S. enteritidis* in the RAW264.7 cells (Figure 2A, Figure S6B-C). In contrast, when ferric iron was added to the macrophages infected with bacteria, lipid ROS levels did not increase in the cells (Figure S6D). However, bacterial proliferation was stimulated under 12.5 µM ferric iron (Figure S6E), consistent with previous research indicating that iron helps intracellular bacteria survive in host macrophages 20.

Ferroptosis inducers, including SSZ 21, APAP 22, and RSL3 23, were also evaluated in the same bacterial infection cell model. To avoid cytotoxicity to macrophages, the concentration and treatment time of the ferroptosis inducers were optimized (Figure S6F-H). When SSZ (5 µM) was added to the infected cells, the intracellular viabilities of *S. aureus*, *E. coli*, *S. pullorum*, and *S. enteritidis* in the RAW264.7 cells were reduced by more than 55%, 85%, 40%, and 80%, respectively (Figure 2B, Figure S6I-J). Similarly, APAP and RSL3 showed significant intracellular inhibition against *S. aureus*, *E. coli*, *S. pullorum*, and* S. enteritidis* when added to the infected cells (Figure 2C, 2D, Figure S6K-N). Ferroptosis inhibitors, such as DFO, EDTA, and Fer-1, were also evaluated using the same model. Results showed that the first two inhibited ferroptosis by removing intracellular ferrous iron, while Fer-1 inhibited ferroptosis by scavenging lipid ROS 24. When EDTA was added, *S. aureus*, *E. coli*, and *S. pullorum* were significantly increased in the RAW264.7 cells (Figure 2E-G, Figure S6O). When DFO and Fer-1 were added, *S. aureus*, *E. coli*, *S. pullorum*, and* S. enteritidis* increased significantly in the RAW264.7 cells (Figure S6P-S). These data demonstrate that ferroptosis inducers can help macrophages suppress intracellular bacteria, while inhibitors can reduce their antibacterial ability without damaging cell morphology (Figure S7). To confirm these findings, TEM characterization of intracellular *S. aureus* treated by ferroptosis inducers or inhibitors was conducted. Compared to the control group, SSZ, APAP, and RSL3 treatment reduced the number of intracellular *S. aureus* with disrupted structures (Figure 2H-I, Figure S8A-B). In contrast, Fer-1 and DFO treatment increased the number of intracellular *S. aureus* with intact morphology (Figure 2H-I, Figure S8A-B). Compared to the control group, ferroptosis inducers reduced the number of intracellular *E. coli* (Figure S8A and C). In contrast, Fer-1 and DFO treatment increased the number of intracellular *E. coli* (Figure S8A and C). Of note, the above ferroptosis inducers and inhibitors showed limited or no direct antibacterial activity against planktonic bacteria. As shown in Figure 2J and K, *S. aureus* and *E. coli* were not sensitive to the inducers and inhibitors, except for SSZ and APAP at high concentrations (10 µM). These results indicate that ferroptosis inducers and inhibitors affect bacterial viability mainly through ferroptotic processes rather than direct inhibition of bacteria. Importantly, our results indicate that transient ferroptosis activity can assist macrophages to suppress invading bacteria, with inhibitory efficiency in the order: *E. coli* > *S. aureus* > S. *pullorum.*

### Ferroptotic stress promotes bone marrow protomacrophages against intracellular bacteria

**Figure 3:**
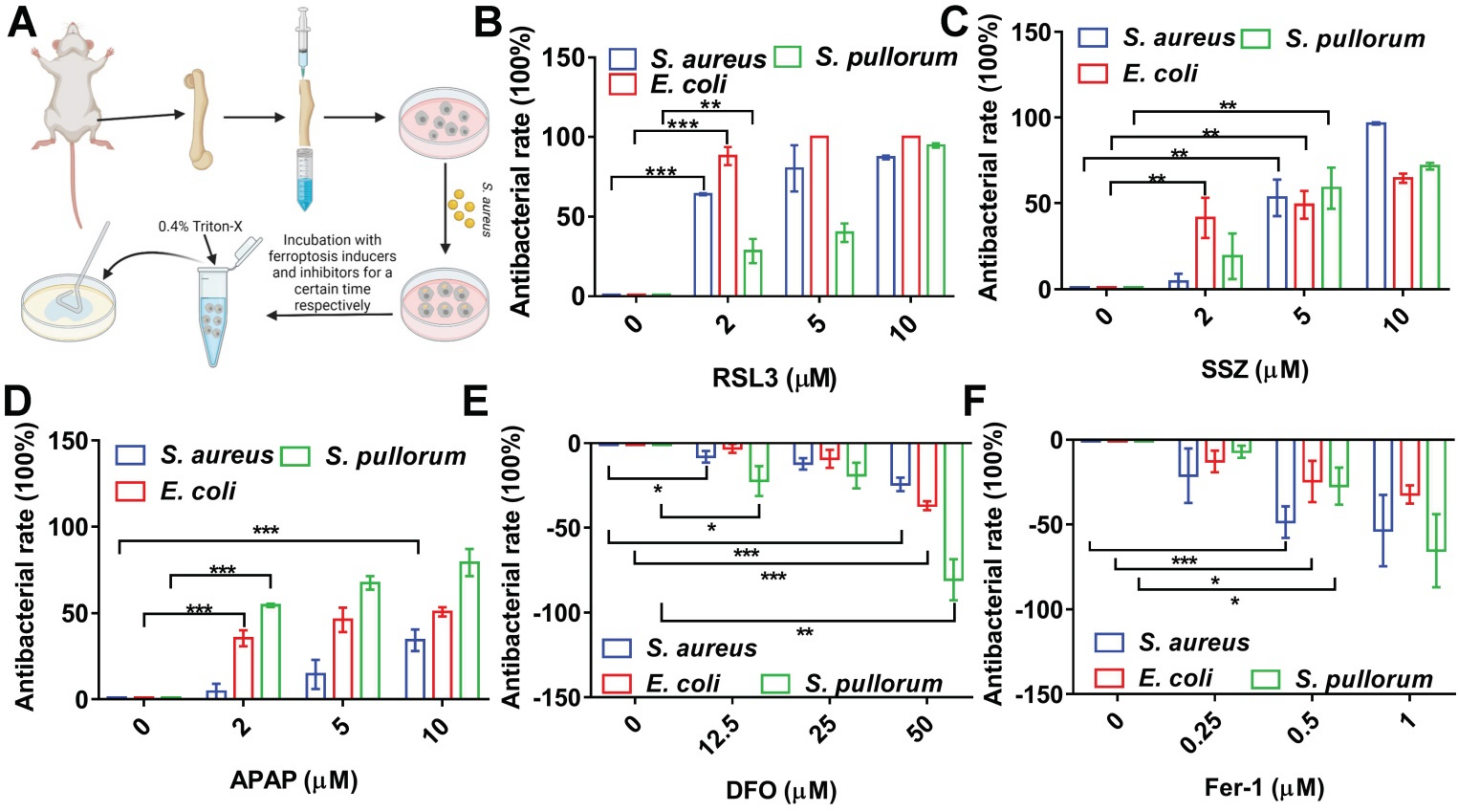
** Intracellular ferroptosis-like death contributes to bacterial suppression in BMMs. (A)** Intracellular bactericidal effects of ferroptosis inducers and inhibitors on BMMs. **(B-D)** Effects of ferroptosis inducers on intracellular bacteria in BMMs. **(E-F)** Effects of ferroptosis inhibitors on intracellular bacteria in BMMs. n = 3, *p < 0.05, **p < 0.01, ***p < 0.001.

To confirm the above conclusion, we investigated the performance of ferroptosis inducers and inhibitors in primary BMMs (Figure 3A). The BMMs were successfully isolated from mouse bone marrow with a purity >95% (Figure S9). To avoid cytotoxicity to BMMs, concentration and treatment time of the ferroptosis inducers were optimized (Figure S10). The BMMs were infected with *S. aureus*, *E. coli*, or *S. pullorum* using the same procedures as for RAW264.7 cells. RSL3 (2 µM) showed significant bacterial inhibition in the BMMs, reducing *S. aureus*, *E. coli*, and *S. pullorum* by more than 60%, 85%, and 30%, respectively (Figure 3B). SSZ and APAP showed similar inhibitory effects (Figure 3C-D). In contrast, when DFO and Fer-1 were added to the infected cells, intracellular *S. aureus*, *E. coli*, and *S. pullorum* were significantly increased (Figure 3E-F). These results provide further evidence that cellular ferroptosis contributes to the restriction of intracellular bacteria in macrophages from cell lines and primary isolation.

### Ferroptotic stress is spatiotemporally transduced to intracellular bacteria via FPN

**Figure 4:**
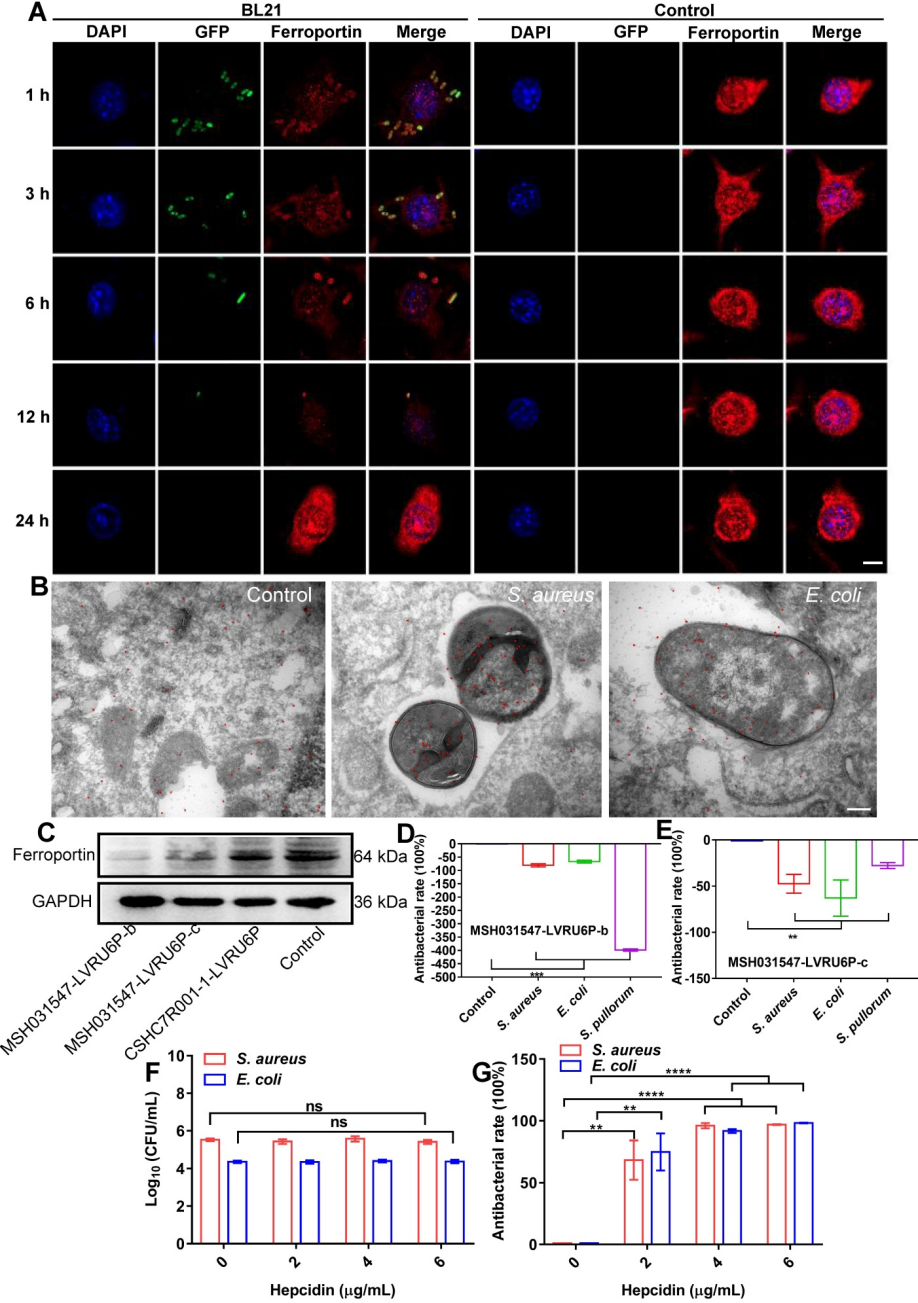
** Dynamic changes in cellular FPN with intracellular bacteria. (A)** Confocal images of FPN co-localized with BL21 over time in macrophages. DAPI-stained cell nucleus. Green fluorescence is BL21. Ferroportin antibody-stained FPN. Scale bar = 5 µm. **(B)** Co-localization of FPN with bacteria by immunoelectron microscopy and colloidal gold labeling. Scale bar = 200 nm** (C)** Knockdown efficiency of FPN by immunoblotting. **(D-E)** Bactericidal efficiency of macrophages after FPN knockdown. **(F)** Intracellular bactericidal effects of hepcidin pre-treatment. **(G)** Intracellular bactericidal effects of hepcidin post-treatment. n = 3, ns = not significant, **p < 0.01, ***p < 0.001, ****p < 0.0001.

The above results indicated that cellular ferroptotic stress leads to suppression of intracellular bacteria. However, bacteria can be found in phagosomes via phagocytosis or in late phagolysosomes via fusion of phagosomes and lysosomes. This raises an interesting question, namely how are ferroptotic signals transmitted to compartmentalized bacteria? We hypothesize that the key factor in ferroptotic stress, i.e., ferrous iron, may be transported to bacterial vesicles. FPN is a major transporter that exports ferrous iron from the cell to extracellular compartments in macrophages 25-27. Thus, we first investigated the co-localization of FPN with internalized bacteria. An *E. coli* strain (BL21) was first engineered with green fluorescence and then used in a RAW264.7 cell infection model, with FPN tested via immunofluorescence imaging. As shown in Figure 4, FPN showed strong signals (red) in the control cells, with distribution mainly on the cell membrane. However, 3-12 h after bacterial infection, the fluorescence intensity of FPN in the whole cell decreased, and FPN and intracellular bacteria showed co-localization. After the infected cells were incubated for 24 h, all intracellular bacteria disappeared (green) and fluorescence intensity and distribution of FPN were restored to that observed in the control (Figure 4A, Figure S11). To further confirm the co-localization of FPN and bacteria, immunoelectron microscopy with colloidal gold-labelling was conducted, which revealed that black colloidal gold particles (red rendering) of FPN were aggregated around the bacteria (Figure 4B). The co-localization of FPN and intracellular bacteria indicates that FPN may transport ferrous iron from the cytoplasm to the bacterial vesicles. Thus, FPN appears to be critical for macrophage defense against intracellular bacteria by transmitting ferroptotic signals to bacteria.

To confirm the role of FPN in macrophage defense against intracellular bacteria, transient knockdown of FPN was performed in RAW264.7 cells by transfecting the MSH031547-LVRU6P-b/c plasmid with transcript siRNA to target FPN mRNA. The knockdown efficiency of the MSH031547-LVRU6P-b/c plasmid was first examined by immunoblotting, which showed that both plasmids effectively reduced FPN expression by 89% and 49%, respectively (Figure 4C, Figure S12). In addition, after FPN knockdown, there was a significant increase in *S. aureus*, *E. coli*, and *S. pullorum* in the infected macrophages. Moreover, the higher the efficiency of FPN knockdown, the more bacteria were counted in the macrophages (Figure 4D-E, Figure S13). Thus, these data suggest that FPN is positively correlated with macrophage suppression of intracellular bacteria.

Hepcidin can regulate FPN protein abundance on the cell surface. Hepcidin promotes the degradation of its own receptor, the only known cellular iron exporter of FPN, resulting in the retention of iron by macrophages. Here, macrophage efficiency against intracellular bacteria was assessed following hepcidin treatment via two models. In the pretreatment model, RAW264.7 cells were incubated with hepcidin for 3 h in advance, followed by *S. aureus*, *E. coli*, or *S. pullorum* infection. Compared to the cells without hepcidin treatment, no reduction in intracellular bacteria was observed in the hepcidin-pretreated cells (Figure 4F, Figure S14A). In the post-treatment model, RAW264.7 cells were infected with *S. aureus*, *E. coli*, or *S. pullorum* and then treated with hepcidin for 3 h. Intracellular bacterial viability was assessed based on CFU number. Results showed that hepcidin (4 µg/mL) treatment led to a significant reduction in intracellular bacteria in the RAW264.7 cells, i.e., 96% for *S. aureus*, 91% for *E. coli*, and 94% for *S. pullorum* (Figure 4G, Figure S14B). The direct antibacterial activity of hepcidin was excluded (Figure S15). Thus, these results suggest that hepcidin helped macrophages defend against intracellular bacteria, but only after bacterial invasion. We speculated that hepcidin may help host cells maintain high levels of ferrous iron by degrading membrane FPN to stop ferrous iron efflux. To confirm this, the degradation efficiency of hepcidin to FPN was measured by immunoblotting. Results showed that FPN was reduced in RAW264.7 cells after bacterial invasion but was further reduced by the addition of hepcidin (post-treatment) (Figure S16A-B). Consistently, based on flow cytometry and FeRhoNox^TM^-1 probes, intracellular ferrous iron levels after hepcidin treatment increased in the RAW264.7 cells infected with *S. aureus*, *E. coli*, or *S. pullorum* (Figure S16C-D). These results indicate that FPN is critical for macrophage defense against intracellular bacteria. Importantly, hepcidin treatment indicated that FPN found around cells may be rearranged on the cell membrane, thus allowing FPN to transport ferrous iron from the cytoplasm into bacterial compartments. Due to changes in FPN, cellular ferroptotic signals can be transmitted to bacterial vesicles to induce bacterial death, characterized by ferrous iron accumulation and lipid ROS production.

### *In situ* ferrous iron induces ferroptosis-like death of intracellular bacteria

**Figure 5:**
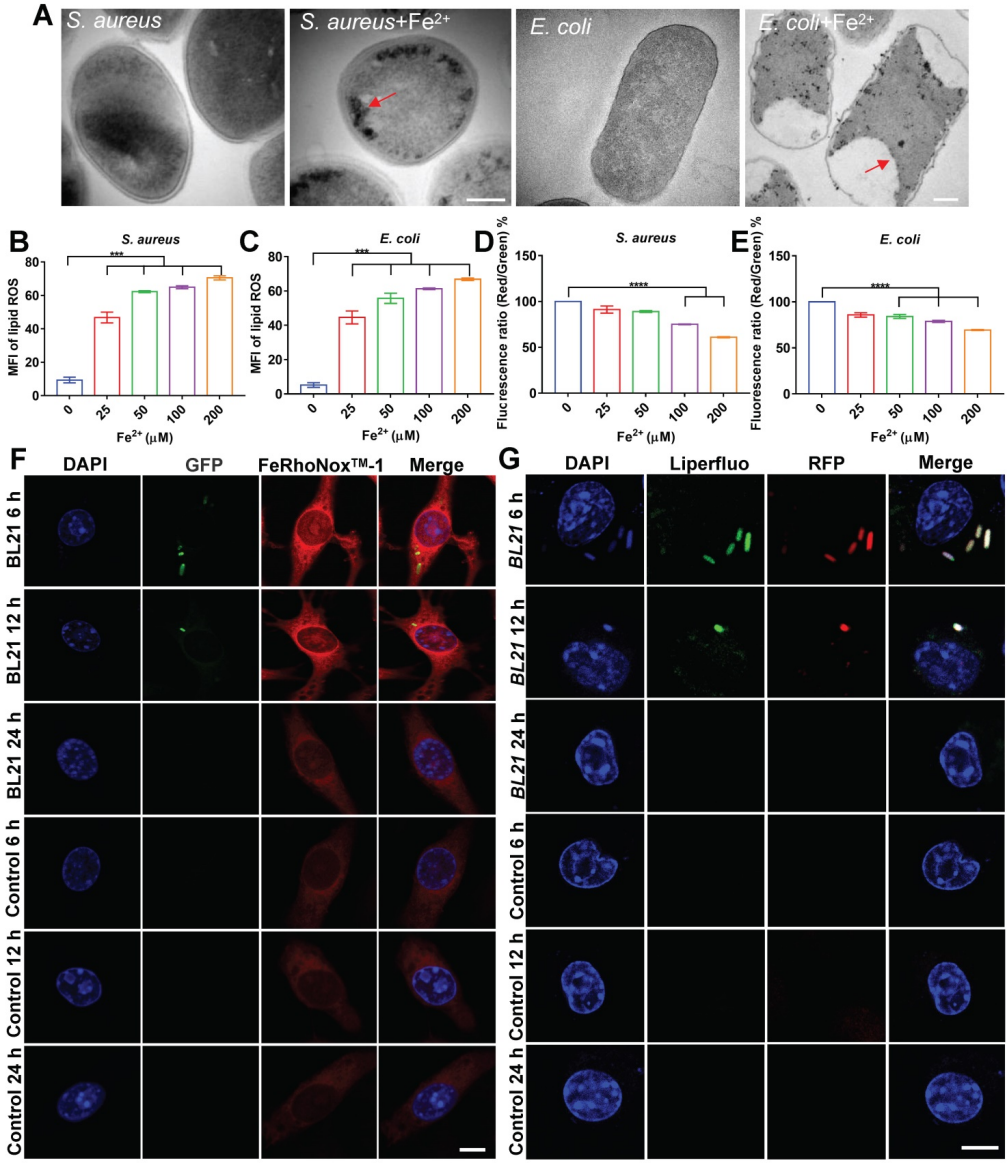
** Ferrous iron induces ferroptosis-like death of intracellular bacteria. (A)** TEM characterization of *S. aureus* and *E. coli* treated by ferrous iron. Scale bar = 200 nm. **(B-C)** Changes in lipid peroxide in bacteria treated with ferrous iron. BODIPY 581/591 C11 probe was used to measure lipid peroxide by a multi-scan spectrum with excitation at 488 nm and emission at 525 nm. **(D-E)** Changes in bacterial membrane potential after treatment with ferrous iron. DIOC_2_(3) probe was used to measure bacterial membrane potential by a multi-scan spectrum with Ex/Em = 480/525 nm (green fluorescence) and Ex/Em = 530/590 nm (red fluorescence). Red/green fluorescence ratios were calculated using population mean fluorescence intensities for bacteria.** (F)** Confocal images of ferrous iron co-located with BL21. DAPI-stained cell nucleus. Green fluorescence is BL21. FeRhoNox^TM^-1 probe-stained ferrous iron. Scale bar = 5 µm. **(G)** Confocal images of lipid peroxides co-located with BL21. DAPI-stained cell nucleus. Liperfluo probe-stained lipid peroxides. Scale bar = 5 µm. n = 3, ns = not significant, ***p < 0.001, ****p < 0.0001.

To confirm our hypothesis, we evaluated the bactericidal effects of ferrous iron on planktonic bacteria under extracellular conditions. After incubation of ferrous iron with* S. aureus*, *E. coli*, or *S pullorum*, TEM was used to characterize bacterial morphology. Results revealed that ferrous iron entered the bacteria and accumulated near the bacterial cell membrane, leading to disruption of its internal structure (Figure 5A, Figure S17A). The SEM results revealed severe bacterial cytoplasmic lysis and leakage of internal components following ferrous iron treatment (Figure S17B). Detection with the BODIPY 581/591 C11 fluorescent probe revealed a more than 4-fold increase in lipid ROS levels after incubation of ferrous iron with bacteria (Figure 5B-C, Figure S17C). Subsequent RNA sequencing showed that ferrous iron had an impact on the expression levels of 3.5% of* S. aureus* genes (1% down-regulated and 2.5% up-regulated) (Figure S18A), which were functionally enriched in more than 20 metabolic pathways, including amino acid biosynthesis, histidine metabolism, aminoglycan and nucleotide sugar metabolism, and vitamin B1 metabolism (Figure S18B). Similarly, ferrous iron affected the expression levels of 10.1% of* E. coli* genes (1.9% down-regulated and 8.2% up-regulated) (Figure S19A), which were functionally enriched in 20 metabolic pathways, including sulfur metabolism, fatty acid degradation, valine, leucine and isoleucine degradation, amino sugar and nucleotide sugar metabolism, and lipid metabolism (Figure S19B). Detection of bacterial membrane potential with the DIOC_2_(3) fluorescent probe revealed that ferrous iron (200 µM) decreased the membrane potential of *S. aureus*, *E. coli*, and *S. pullorum* by 39%, 31%, and 30%, respectively (Figure 5D-E, Figure S17D). Bacterial LDH release was assayed after ferrous treatment. Results revealed an increase in LDH activity in the supernatant, indicating LDH release and disruption of bacterial cell membrane integrity (Figure S17E-F). In addition, bacterial viability was also measured using a live/dead staining assay. Confocal images showed an increase in PI fluorescence intensity with increasing ferrous concentration, suggesting that the number of dead bacteria was positively correlated with ferrous iron (Figure S20). Comparing the bactericidal effects of different concentrations of ferrous and ferric iron, we found that ferric iron had no bactericidal effect, whereas 25 µM ferrous iron killed all *S. aureus* and *E. coli*, and 2-Log toward *S. pullorum*, indicating that only ferrous iron had bactericidal activity (Figure S17G-I). Thus, the above results demonstrate that the interaction between ferrous iron and bacteria can induce ferroptosis-like death in bacteria with the hallmarks of ferrous iron dependence, lipid peroxidation, and membrane shrinkage. To verify ferroptotic death, EDTA was added to the mixture of ferrous iron and bacteria. Bacterial viability assay showed that bacterial killing was dramatically inhibited (Figure S17J-L). Importantly, Fer-1, a typical inhibitor of ferroptosis in mammalian cells, significantly inhibited the antibacterial activity of ferrous iron (Figure S17M-O). These results indicate that certain iron chelators and ferroptosis inhibitors can negatively regulate the antibacterial reactivity of ferrous iron, thus providing counterevidence that ferrous iron directly induces ferroptotic damage in bacteria.

To confirm this, the co-localization of ferrous iron with the BL21 strain in infected cells was investigated. FeRhoNox^TM^-1 was used as a probe of intracellular ferrous iron. Fluorescent imaging showed that the intracellular fluorescence intensity of ferrous iron increased considerably in the whole cell, and the BL21 bacteria were located in the area of high ferrous probe density in the early stage of infection (post 6-12 h). In contrast, at 24 h post infection, all bacteria inside the cells disappeared and the fluorescence intensity of ferrous iron decreased to the level found in the control cells (Figure 5F, Figure S21).

The co-localization of lipid peroxide and bacteria was also investigated. Infected cells were labelled with the Liperfluo fluorescent probe (green), which demonstrated that intracellular lipid ROS increased and co-localized with BL21 when infected cells were cultured for 6-12 h. Similar to the dynamic changes in ferrous iron, all bacteria inside the cells were killed and the fluorescence intensity of lipid ROS was reduced after 24 h (Figure 5G). Surprisingly, the dynamic changes in lipid ROS did not lead to host cell death. Cytotoxicity assays revealed that the RAW264.7 cells infected with *S. aureus* and *E. coli* maintained 95% and 89% viability at 24 h, respectively (Figure S22), indicating that despite intracellular lipid ROS production, the cells were not damaged. These results suggest that bacteria invading macrophages at the early stage can lead to the co-localization of FPN with internalized bacteria, accompanied by ferrous iron and lipid ROS production.

### Ferroptotic stress is temporally regulated in macrophages

**Figure 6:**
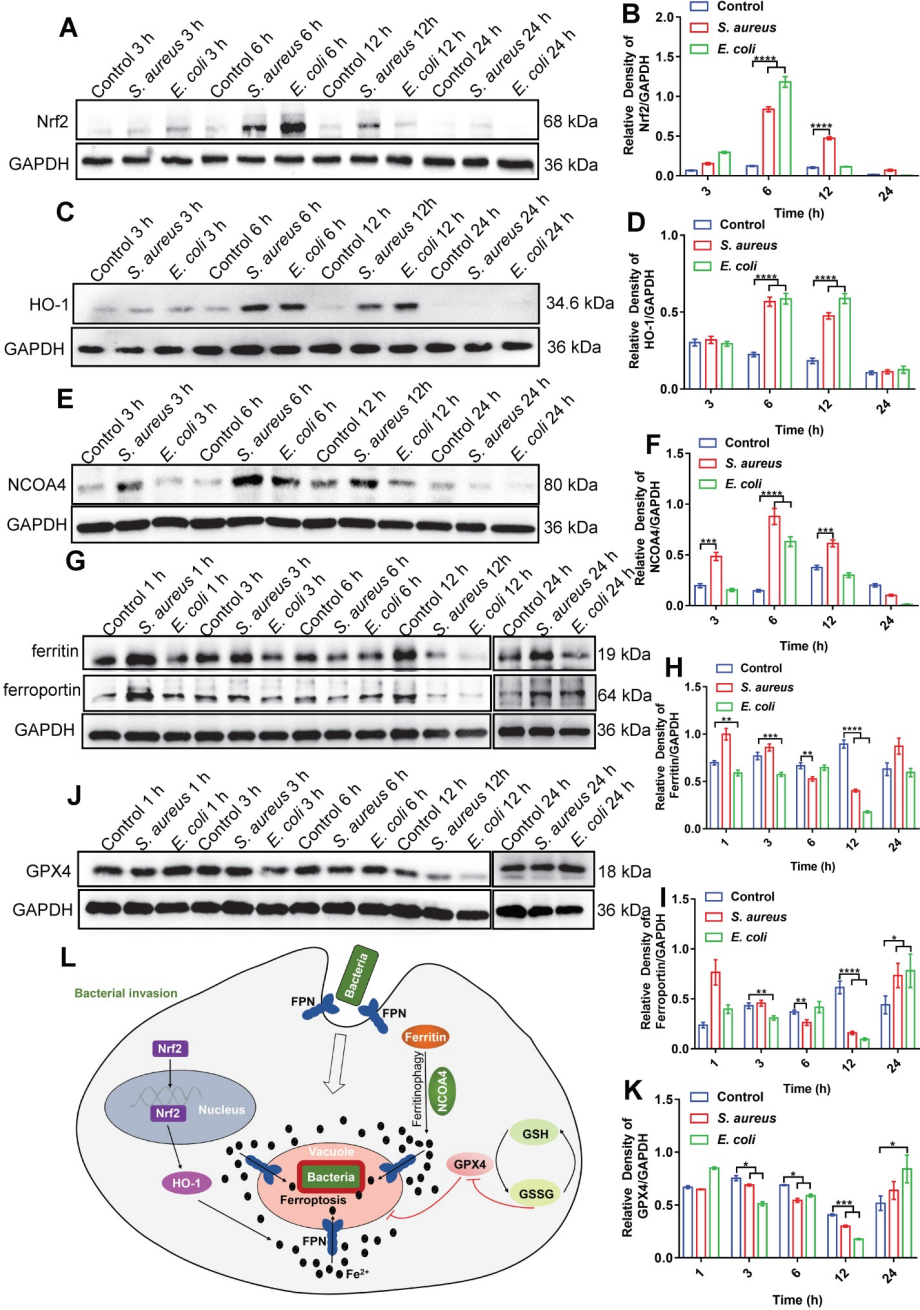
Bacteria-infected RAW264.7 cells increase intracellular ferrous iron levels via three pathways: NCOA-4-ferritin, Nrf2-HO-1, and FPN inhibition. **(A-K)** Immunoblotting of Nrf2, HO-1, NCOA-4, FPN, ferritin, and GPX4 protein expression. **(L)** Mechanism of macrophage regulation of intracellular ferroptosis-like death in bacteria.

As ferrous iron is a key factor in intracellular bacterial death but not in ferroptotic death of the host cell, the signaling pathways related to ferroptosis may be involved in iron regulation. The transferrin receptor (TfR) is present on the cell membrane and mediates the transportation of iron from the extracellular to intracellular compartment. Ferric iron binds to transferrin (Tf) on the cell membrane to form Tf-Fe^3+^, and combines with the membrane protein Tf receptor 1 (TfR1), which enters the nuclear endosome under endocytosis. Fe^3+^ is then reduced to Fe^2+^ by six-transmembrane epithelial antigen of prostate 3 (STEAP3), which is then stored in the unstable iron pool and ferritin 27-29. Thus, we first investigated changes in the TfR in macrophages after bacterial infection. Here, *E. coli* bacteria were used in a RAW264.7 cell infection model via immunofluorescence imaging. Results showed no significant change in the fluorescence intensity of TfRs in the whole cells after bacterial infection (Figure S23). Thus, the increase in intracellular ferrous iron level after bacterial infection may not rely on TfR-mediated transportation of iron from the extracellular to intracellular compartment, but rather through the regulation of intracellular iron metabolism-related proteins. We further investigated the nuclear factor erythroid 2-related factor 2 (Nrf2)/heme oxygenase-1 (HO-1) axis, which regulates the iron pathway in ferroptosis 30. Nrf2 is a nuclear transcription-associated protein that can enter the nucleus to up-regulate HO-1, which then degrades heme into ferrous iron, CO, and bilirubin 27, 31. Western blot analysis of Nrf2 (in the nucleus) and HO-1 showed that the expression levels of both proteins increased in the RAW264.7 cells from 3-12 h after bacterial infection, then gradually recovered at 24 h (Figure 6A-D). In addition, we investigated the ferritin-nuclear receptor coactivator-4 (NCOA-4) axis. As an important iron storage protein, ferritin plays a central role in the biological management of iron. NCOA-4 binds to ferritin and delivers it to nascent autophagosomes, which then merge with lysosomes for ferritin degradation and iron release 32. Here, western blotting showed that NCOA-4 protein expression increased in the RAW264.7 cells at 3-12 h after bacterial infection, then decreased at 24 h (Figure 6E-F). In contrast, western blotting and immunofluorescence imaging showed that ferritin protein expression decreased in the RAW264.7 cells at 3-12 h after bacterial infection, then gradually increased at 24 h (Figure 6G-H, Figure S24), indicating that the ferritin-NCOA-4 axis may contribute to iron accumulation in macrophages during the early stages of bacterial invasion. Thus, both the Nrf-2/HO-1 and ferritin/NCOA-4 axes are activated in the early stage of bacterial infection in macrophages to increase ferrous iron to a high level.

In addition to generating ferrous iron, retaining ferrous iron inside the cell is also important. As such, we analyzed changes in FPN in the RAW264.7 cells during bacterial infection by western blotting. As expected, FPN abundance decreased in the RAW264.7 cells at 3-12 h after bacterial infection, and then increased at 24 h (Figure 6G and I), indicating that ferrous iron efflux was inhibited in the early stage of bacterial infection. With the accumulation of ferrous iron, lipid peroxidation will trigger subsequent cell death (ferroptosis). Here, however, death was only triggered in the intracellular bacteria, with the host cells managing to avoid ferroptotic death. We speculate that the antioxidant pathway may be activated to scavenge lipid peroxidation damage. To confirm this, we analyzed GSH peroxidase 4 (GPX4), an important enzyme that catabolizes lipid peroxide using GSH as a substrate 33. Results showed that GPX4 protein expression gradually decreased in the RAW264.7 cells at 12 h after bacterial infection, but then increased to normal levels at 24 h, which may reduce lipid peroxidation at the late stage of bacterial infection and protect macrophages from ferroptotic death (Figure 6J-K). These results indicate that ferroptotic stress is well controlled in macrophages upon bacterial infection, i.e., by increasing ferrous iron levels at the early stage of infection via pro-ferroptotic pathways (e.g., Nrf2/HO-1 and ferritin/NCOA-4 axes), retaining intracellular ferrous iron by reducing FPN-mediated efflux, and protecting cells from ferroptotic damage by instigating anti-ferroptotic pathways (e.g., GPX4 expression) (Figure 6L, Video S1).

### *In vivo* bacterial infection induces ferroptotic stress and can be treated with ferroptosis inducers

**Figure 7:**
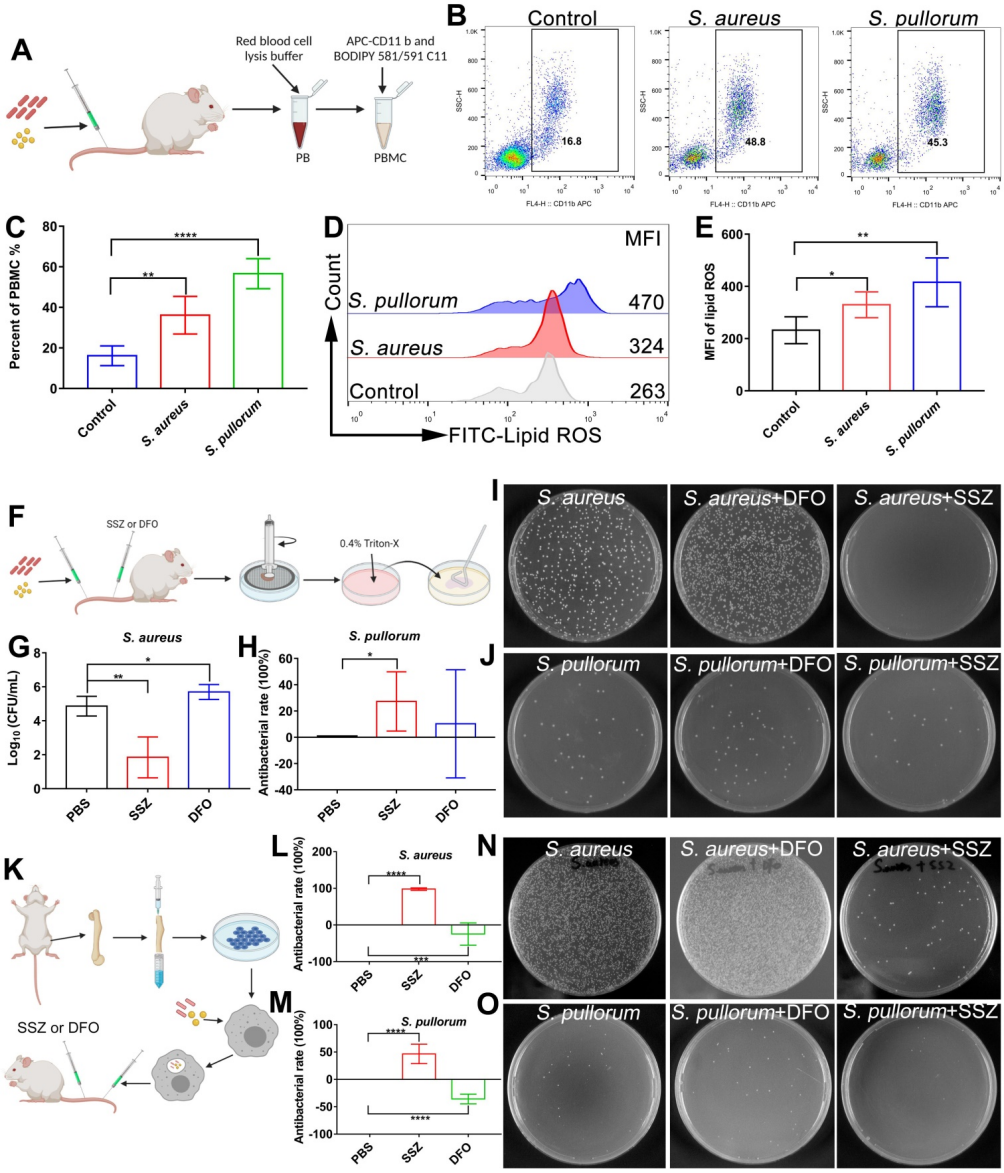
** Regulating ferroptosis stress for *in vivo* treatment of bacterial infection. (A)** Schematic of peripheral blood assay in mice infected (via tail vein) with *S. aureus* or* S. pullorum* at logarithmic growth stage. Peripheral blood was taken from mice 24 h after infection and red blood cell lysis buffer was used to lyse erythrocytes. Peripheral blood macrophages and lipid ROS were labelled with APC CD 11b and BODIPY 581/591 C11 fluorescent probes, respectively. Lipid ROS changes in peripheral blood macrophages were detected by flow cytometry.** (B-C)** Macrophage ratio in peripheral blood after infection.** (D-E)** Lipid ROS levels in peripheral blood macrophages after infection. **(F)** Schematic of SSZ and DFO treatment in bacterial-infected mice. **(G-H)** Bacterial inhibition efficiency of *S. aureus* and *S. pullorum* in kidneys treated with SSZ or DFO at 72 h.** (I-J)** LB solid agar plate to observe number of bacteria in kidneys after 72 h of SSZ or DFO treatment. **(K)** Experimental design of intracellular bacterial infection model and treatment with SSZ or DFO. **(L-M)** Bacterial inhibition efficiency of *S. aureus* and *S. pullorum* in kidneys treated by SSZ or DFO at 72 h. **(N-O)** LB solid agar plate to observe number of bacteria in kidneys after 72 h of SSZ or DFO treatment. n = 5 mice per group, **p < 0.01, ****p < 0.0001.

As ferroptosis stress contributes to macrophage defense against invasive bacteria, we explored whether *in vivo* bacterial infection can be treated by ferroptosis regulators. Firstly, lipid ROS levels in peripheral blood macrophages were measured in mice after intravenous infection by *S. aureus* or* S. pullorum* in the tail vein. Peripheral blood was taken from mice 24 h after infection and peripheral blood macrophages were collected for labelling with APC CD 11b antibodies and BODIPY 581/591 C11 probes, respectively (Figure 7A). Flow cytometry showed that lipid ROS levels were significantly increased in peripheral blood macrophages after bacterial infection, accompanied by an increase in the proportion of macrophages in peripheral blood (Figure 7B-E). These results indicate that ferroptotic stress indeed occurs in macrophages upon bacterial infection under *in vivo* conditions.

Regulation of ferroptosis via inducers and inhibitors was validated using an *in vivo* bacterial infection model. To determine the optimal treatment dose, after bacterial infection, the mice were administered tail vein injections of 100 µL of SSZ (10, 50, and 100 µM) or DFO (10, 50, and 100 µM), respectively. By analyzing bacterial viability in the hearts, livers, spleens, lungs, and kidneys of mice, the highest inhibition efficiency was achieved after treatment with 100 µM SSZ or DFO (Figure S25A-D). Therefore, 100 µM SSZ and DFO was chosen for the following investigation. Briefly, mice were infected with *S. aureus* and *S. pullorum* (at the logarithmic growth stage) via the tail vein, respectively. After 12 h of infection, 100 μL of SSZ (ferroptosis inducer, 100 µM) or 100 μL of DFO (ferroptosis inhibitor, 100 µM) was administered intravenously twice a day, with a total of five treatments per mouse (Figure 7F). By analyzing bacterial viability in the kidneys of mice, the SSZ group exhibited a 99% reduction in *S. aureus* and 27% reduction in *S. pullorum* compared to mice treated with PBS alone (Figure 7G-J). SSZ treatment also resulted in smoother fur than that in the PBS-treated control group (Figure S25E-F). In contrast, DFO treatment enhanced *S. aureus* viability (Figure 7G and I) and resulted in scruffier fur compared to that in the control group (Figure S25E). Bacterial inhibition efficiency was also examined in different organs. By analyzing bacterial viability in the heart (without *S. aureus*), liver, spleen, and lungs of the mice, the SSZ-treated group exhibited a reduction in *S. aureus* and *S. pullorum* compared to those mice treated with PBS alone (Figure S25A-D). In contrast, DFO treatment enhanced the viability of *S. aureus* and *S. pullorum* (Figure S25A-D).

Furthermore, the heart, liver, spleen, lungs, and kidneys of bacteria-infected mice were taken for H&E staining to analyze pathological changes. As shown in Figure S26, the liver structure of *S. aureus*-infected mice was destroyed, with hepatocyte necrosis (short arrow), central venous congestion (arrow), and massive inflammatory cell infiltration (long arrow). These symptoms disappeared after SSZ treatment. The liver structure of *S. pullorum*-infected mice was destroyed, with hepatocyte necrosis (short arrow) and central venous congestion (arrow). SSZ treatment reduced central venous congestion. Mature and immature megakaryocytes were increased in the spleen of *S. aureus*-infected mice but decreased after SSZ treatment and increased after DFO treatment. The same changes were observed after *S. pullorum* infection. There were no significant pathological changes in the lungs of *S. aureus*-infected mice, and no significant pathological changes in the lungs after SSZ treatment. However, the lungs of DFO-treated mice showed alveolar cavity hemorrhage (arrow). Kidney sections from *S. aureus*-infected mice exhibited renal cell necrosis (long arrow), which was reduced by SSZ treatment. In contrast, the area of necrosis was not reduced after DFO treatment (long arrow) and inflammatory cell infiltration foci appeared (short arrow). In summary, the liver, kidney, and spleen of bacteria-infected mice showed varying degrees of pathological changes. The lesions were reduced after SSZ treatment but aggravated after DFO treatment.

Next, we constructed an *in vivo* bacterial infection model with macrophages. As shown in Figure 7k, BMMs were infected with *S. aureus* or *S. pullorum*, followed by gentamycin treatment to kill extracellular bacteria while retaining viable bacteria inside the cell. The BMMs (10^5^ cells/mouse) were intravenously injected into Balb/C mice. After 12 h of infection, 100 μL of SSZ (100 µM) or 100 μL of DFO (100 µM) was administered intravenously twice a day, with a total of five treatments per mouse (Figure 7K). Compared to mice treated with PBS alone, the SSZ group showed a 90% reduction in *S. aureus* and 45% reduction in *S. pullorum* viability in the kidneys (Figure 7L-O). SSZ treatment also resulted in faster restoration of body weight and smoother fur compared to that in the PBS-treated control group (Figure S27A-B). In contrast, DFO treatment enhanced *S. aureus* and *S. pullorum* viability (Figure 7L-O) and resulted in greater weight loss and scruffier fur in *S. aureus*-infected mice compared to the control group (Figure S27A-B). These results indicate that regulating the ferroptosis response with inducers may help treat bacterial infection *in vivo*.

To evaluate *in vivo* biocompatibility, we administered SSZ (100 µM) and DFO (100 µM) in healthy Balb/c mice and examined their influence on body weight, complete blood count, and major organs. Results showed that SSZ and DFO administration did not affect body weight (Figure S28A), complete blood count (Figure S28B-I), or major organs, including the liver, lung, spleen, and kidneys (Figure S28J), thus demonstrating high biocompatibility.

## Discussion

In the current study, we identified a general antibacterial strategy in macrophages, which transmits the cytosol ferroptosis messenger (ferrous iron) into intracellular bacterial compartments via FPN to induce ferroptosis-like bacterial death. Ferroptosis is a recently discovered type of programmed cell death with the hallmark of ferrous iron-dependent lipid peroxidation 34, 35. It occurs in many mammalian and plant cells and is associated with diseases such as cancer, neurodegeneration, kidney injury, and cardiovascular injury 36. Ferroptosis is often found in eukaryotic systems but rarely in prokaryotic cells, such as bacteria 37. Moreover, bacterial infection is reported to cause ferroptosis in host cells 38. Although the pathological consequences of ferroptosis are relatively well known, the physiological role of ferroptosis remains unclear, particularly in bacterial infections 39, 40. As primary regulators of iron homeostasis, macrophages are thought to be insusceptible to ferroptosis 41. In addition, macrophages boost ROS production to suppress enteric pathogens. Recent studies have found FPN located with *Salmonella*-containing vacuoles (SCV) in macrophages, in which the hepcidin-FPN axis transports iron from the cytosol to SCV and generates ROS via Fenton reaction with hydrogen peroxide produced by NADPH oxidase (NOX) 14. Consistently, we found that FPN and intracellular bacteria were co-localized and that FPN was internalized along with bacterial vesicles from the membrane. However, we discovered that ferrous iron directly killed bacteria with the hallmarks of ferrous iron dependence, lipid peroxidation, and membrane malfunction, thereby demonstrating ferroptosis-like death in bacteria (Figure 5A-C). These results indicate that bacterial death caused by ferrous iron transported inward by FPN may be independent of NOX, which generates hydrogen peroxide as a substrate for Fenton reaction. Importantly, ferroptotic stress in macrophages provided abundant ferrous iron in the cytosol at the early stage of bacterial invasion (Figure 1A-E), thus ensuring a source of iron for FPN to transport into the bacterial compartments. Hence, ferrous iron acts like a second messenger and delivers ferroptotic stress to intracellular bacteria.

The coordination of cytosol ferroptosis stress and ferroptosis-like death of intracellular bacteria was also confirmed using ferroptosis inducers and inhibitors. At the cellular level, adding a certain concentration (without damaging host cells) of ferrous iron or ferroptosis inducers (SSZ, APAP, and RSL3) increased lipid ROS levels and helped in macrophage defense against intracellular bacteria. In contrast, ferroptosis inhibitors, which can chelate ferrous iron (EDTA, DFO) or reduce lipid ROS (Fer-1), demonstrated adverse effects. Thus, regulating ferroptosis activity in macrophages may be an effective strategy against intracellular bacteria at the early stage of infection. Furthermore, administration of the ferroptosis inducer SSZ in infected mice at 72 h significantly suppressed bacterial viability *in vivo* (Figure S25A-D) by maintaining lipid ROS levels in peripheral blood macrophages (Figure S29). These preliminary results indicate that ferroptosis stress was not aggravated by using ferroptosis inducers. In fact, ferroptosis inducers may help avoid organ damage due to bacterial infection (Figure S26), indicating that administration of ferroptosis inducers could be beneficial for antibacterial therapy. However, bacterial infection and ferroptotic stress are dynamic processes involving many signaling pathways, especially in the immune system. Thus, in-depth study and comprehensive evaluation and tracking of ferroptotic stress under *in vivo* conditions are required.

Through FPN-mediated inward iron transportation, ferroptotic damage was successfully transmitted to compartmentalized bacteria. However, ferroptotic stress in the macrophages was terminated in the late stage (after 12 h) of bacterial invasion. The hallmarks of ferroptotic stress, including iron concentration, lipid ROS level, and mitochondrial potential, recovered at 24 h post-infection (Figure 1F-I). Consistently, the Nrf2/HO-1 and ferritin/NCOA-4 axes (controlling iron release) and GPX4 expression level all recovered at 24 h post-infection. However, it is still unclear how macrophages sense the environment to terminate ferroptosis stress, as not all bacteria are killed in the macrophages. In addition, although macrophages do not experience ferroptosis, bacteria showed different susceptibilities to ferroptosis-like death. Our results revealed that *E. coli* was eliminated in macrophages after 24 h of infection, followed by* S. aureus*. In contrast, *S. pullorum* was difficult to kill under intracellular conditions, consistent with previous studies suggesting that *Salmonella* specie*s* are persistent pathogens in host cells 42-44. Therefore, future study is required to elucidate how cells temporally regulate ferroptosis and why some bacteria tolerate ferroptotic damage.

## Conclusions

Overall, our findings suggest that ferroptotic stress is beneficial for macrophages against intracellular bacteria, as the stress can be transferred from the cytosol to vacuole containing intracellular bacteria via FPN transportation. This mechanism ensures effective suppression of intracellular bacteria but is harmless to the macrophages. Therefore, targeting ferroptotic stress may be a potential strategy to treat infections with intracellular pathogens.

## Supplementary Material

Supplementary methods and figures.

Supplementary video.

## Abbreviations

*S. aureus*: *Staphylococcus aureus*
*E. coli*: *Escherichia coli*
*S. pullorum*: *Salmonella pullorum*
MMP: mitochondrial membrane potential
CFUs: colony forming units
SSZ: sulfasalazine
RSL3: Ras-selective lethal small molecule 3
APAP: acetaminophen
DFO: deferoxamine
EDTA: ethylenediaminetetraacetic acid
Fer-1: ferrostatin-1
BMMs: bone marrow protomacrophages
FPN: Ferroportin
TEM: transmission electron microscopy
SEM: scanning electron microscopy
Nrf2: nuclear factor erythroid 2-related factor 2
HO-1: heme oxygenase-1
NCOA4: ferritin-nuclear receptor coactivator-4
GPX4: GSH peroxidase 4
PBS: phosphate-buffered saline
SCV: *Salmonella*-containing vacuoles
NOX: NADPH oxidase
FBS: fetal bovine serum
CCK8: Cell Counting Kit-8
DDSA: dodecyl succinic anhydride
NMA: N-methylolacrylamide
BDMA: benzyldimethylamine
BSA: albumin bovine V
PVDF: polyvinylidene fluoride
TBST: Tris-buffered saline containing 0.05% Tween 20
HRP: horseradish peroxidase
MFI: mean fluorescence intensity
Hb: hemoglobin
MCH: mean corpuscular hemoglobin
MCV: mean corpuscular volume
RBC: red blood cell
HCT: hematocrit
WBC: white blood cell
PLT: platelet count
NEUT: neutrophilic granulocyte
