# Supplementary material for: Ferroptotic stress promotes macrophages against intracellular bacteria
Source: Theranostics. 2022 Feb 21;12(5):2266–89. doi: 10.7150/thno.66663 (PMC8899587; doi:10.7150/thno.66663)
Supplement: Supplementary file 1 — Supplementary methods and figures. [file thnov12p2266s1.pdf]

Supplementary Information

Ferroptotic stress promotes macrophages against intracellular bacteria

Ruonan Ma[1,2]†, Ling Fang [1,2]†, Lei Chen[2], Xiaonan Wang[2], Jing Jiang[2], Lizeng Gao[1,2,3]*

[1]Institute of Translational Medicine, Department of Pharmacology, School of Medicine, Yangzhou University, China

[2]CAS Engineering Laboratory for Nanozyme, Institute of Biophysics, Chinese Academy of Sciences, China.

[3]Joint Laboratory of Nanozymes in Zhengzhou University, Academy of Medical Sciences, Zhengzhou University, China

*Correspondence to: Email: gaolizeng@ibp.ac.cn (L.G.)

†These authors contributed equally to this work.

Supplementary methods

Extracellular bacterial killing with gentamicin

Single colonies of *S. aureus* (ATCC 29213), *E. coli* (CMCC (B)44102), and *Salmonella pullorum* (S06004, a kind gift from Prof. Xinan Jiao's laboratory in Yangzhou University [1]) (*S. pullorum*) on LB solid agar plates were randomly selected and inoculated in 5 mL of LB liquid culture medium and cultured at 37°C for 12 h under 180 rpm rotation. The following day, 50 μL of the bacterial inoculation was diluted with 4 950 μL of fresh LB medium and cultured at 37°C for 3 h at 180 rpm until OD600 reached 0.8. The bacterial precipitate was obtained by centrifugation at 3 600 rpm for 5 min at 4°C and diluted to 2×10^8 CFUs/mL with 2.5 mL of RPMI 1640 culture medium. Then, 100 μL of bacterial inoculation was mixed with 900 μL of RPMI 1640 containing 1% FBS as the control. Another 100 μL of bacterial inoculation was mixed with 900 μL of RPMI 1640 containing gentamicin (200 μg/mL) and 1% FBS as the experimental group. After incubation at 37°C for 1 h and 3 h, bacterial viability was checked by plating bacteria at a proper dilution and calculating the bacterial number in CFUs/mL.

The effect of gentamicin on intracellular bacterial activity was then examined. RAW264.7 cells were inoculated in 6-well plates containing 1 mL of complete culture solution (RPMI 1640 + 20% FBS + 1% penicillin/streptomycin). At the logarithmic growth phase, the RAW264.7 cells were infected with *S. aureus*, *E. coli*, and *S. pullorum* at MOI = 20:1 for 1 h, respectively, with the extracellular bacteria killed with gentamicin (200 μg/mL) for 3 h. After washing three times with PBS, the cells were cultured at 37°C under 5% CO_2 in 1 mL of RPMI 1640 containing 1% FBS for 0.5, 1, and 2 h. A sample of the cells was lysed using 1 mL of buffer containing 0.4% Triton X-100. The number of viable bacteria was counted by plating serial dilutions of bacteria on LB solid medium.

LC3 protein measurement

RAW264.7 cells were inoculated into a 6-well plate containing 1 mL of complete culture medium (RPMI 1640 + 20% FBS + 1% penicillin/streptomycin). Cells were then cultured at 37°C under 5% CO_2 to 80%–90% confluence, thus achieving a cell count of 1×10^6. The culture was then replaced with 1 mL of RPMI 1640 containing 1%, 2.5%, and 20% FBS and incubated for 24 h at 37°C. After washing with ice-cold PBS, the cells were lysed in RIPA buffer (Beyotime Biotechnology) containing protease inhibitor. The lysate was centrifuged at 12 000 g for 10 min at 4°C and the protein concentrations were determined and calibrated using a BCA Protein Assay Kit. Protein extracts were resolved on SDS-polyacrylamide gels, transferred to PVDF membranes, and blocked for 1 h with 0.05% TBST and 5% skimmed milk powder. The PVDF membranes were washed three times with TBST (10 min each time). The LC3 primary antibodies (Abcam) were diluted 1:1 000 in TBST (containing 5% BSA) and incubated overnight at 4°C. After washing with TBST, the membranes were incubated with HRP-conjugated goat anti-rabbit (Abcam) secondary antibodies (1:1000) in TBST for 1 h at room temperature. Protein bands were visualized using a fully automated chemiluminescence image analysis system (Tanon-4600SF).

Internalization of bacteria by macrophage and intracellular assays

RAW264.7 cells were inoculated in 6-well plates containing 1 mL of complete culture solution (RPMI 1640 + 20% FBS + 1% penicillin/streptomycin). At the logarithmic growth phase, the

RAW264.7 cells were infected with *S. aureus*, and *E. coli* at MOI = 20:1 for 1 h, respectively, with the extracellular bacteria killed with gentamicin (200 μg/mL) for 3 h. After washing three times with PBS, the cells were cultured at 37°C under 5% CO_2 in 1 mL of RPMI 1640 containing 1% FBS for 1, 3, 6, 12, and 24 h. Intracellular ferrous iron was also detected with a FeRhoNox™-1 fluorescent probe (GORYO Chemical). Then, 200 μL of 5 μM FeRhoNox™-1 working solution was added to the cells, followed by 45 min of incubation at 37°C. Intracellular ferrous fluorescence intensity was measured using flow cytometry (BD FACSCalibur, USA). Intracellular lipid peroxidation levels were detected with a BODIPY 581/591 C11 probe (Invitrogen), with 200 μL of 2 μM BODIPY 581/591 C11 probe working solution added to the cells with 30 min of incubation at 37°C. Fluorescence intensity of lipid ROS was detected by flow cytometry (BD FACSCalibur). Cell activity following bacterial infection was assayed with a Cell Counting Kit-8 (CCK-8) (DOJINDO).

***In vitro* antibacterial assays**

Single colonies of *S. aureus*, *E. coli*, and *S. pullorum* on LB solid agar plates were randomly selected and inoculated in 5 mL of LB liquid medium and cultured at 37°C for 12 h under 180 rpm rotation. The next day, 50 μL of the bacterial inoculation was diluted with 4 950 μL of fresh LB medium and cultured at 37°C for 3 h at 180 rpm until OD600 reached 0.8. Then, 100 μL of bacterial inoculation was mixed with H_2O (900 μL) as the control. Another 100 μL of bacterial inoculation was mixed with hepcidin (Abcam) (100 μL) (2, 4, and 6 μg/mL) and H_2O (800 μL) as the experimental group. After incubation at 37°C for 30 min, bacterial viability was checked by plating bacteria at a proper dilution and calculating the bacterial number in CFUs/mL. Inhibition assays with 0.4% Triton X-100 (Beyotime Biotechnology) were conducted using the same system and procedure. Inhibition assays with Fe^{2+} in combination with EDTA or Fer-1 also used under the same system and procedure, although in addition to 100 μL of Fe^{2+} (12.5 μM), an additional 100 μL of EDTA or Fer-1 and 700 μL of H_2O were added to maintain the system at 1 mL.

Bacterial characterization with scanning (SEM) and transmission electron microscopy (TEM)

Single colonies of *S. aureus* and *E. coli* on LB solid agar plates were randomly selected and inoculated in 5 mL of LB liquid medium and cultured at 37°C for 12 h under 180 rpm rotation. The next day, 50 μL of the bacterial inoculation was diluted with 4 950 μL of fresh LB medium and cultured at 37°C for 3 h at 180 rpm until OD600 reached 0.8. The bacterial precipitate (1 mL) was obtained by centrifugation at 3 600 rpm for 5 min at 4°C and diluted with H_2O (900 μL). Then, 100 μL of Fe^{2+} (50 μM) was mixed with *S. aureus* (900 μL) and *E. coli* (900 μL) at 37°C for 30 min, respectively. Morphology was examined by SEM. First, the bacterial suspension was resuspended in glutaraldehyde (2.5%, Sigma-Aldrich) for 24 h at 4°C under dark conditions. Bacterial cells were then washed and treated with ethanol gradient dehydration (30%, 50%, 70%, 90%, and 100% twice), followed by drying with a critical point dryer. Finally, the bacterial cells were sputter-coated with platinum and analyzed via SEM (Hitachi-S4800) at a working voltage of 15.0 kV and working current of 10 μA under 40 K magnification [2].

The internal structures of *S. aureus*, *E. coli*, and *S. pullorum* incubated with Fe^{2+} (50 μM) were observed by TEM. Cells were fixed with 2.5% (vol/vol) glutaraldehyde with phosphate buffer (PB)

(0.1 M, pH 7.4) and washed four times in PB. The cells were then postfixed with 1% (wt/vol) osmium tetroxide in PB for 2 h at 4°C and dehydrated through a graded ethanol series (30%, 50%, 70%, 80%, 90%, 100%, and 100%, 7 min each) in pure acetone (2 × 10 min). Samples were infiltrated in graded mixtures (3:1, 1:1, and 1:3) of acetone and SPI-PON812 resin (16.2 g of SPI-PON812, 10 g of dodecyl succinic anhydride (DDSA), and 8.9 g of N-methylolacrylamide (NMA). Finally, the cells were embedded in pure resin with 1.5% benzyldimethylamine (BDMA) and polymerized for 12 h at 45°C and 48 h at 60°C. Ultrathin sections (70 nm thick) were produced using a microtome (Leica EM UC6), double-stained using uranyl acetate and lead citrate, and examined using TEM (FEI Tecnai Spirit 120 kV).

RNA-seq for transcriptome analysis

Single colonies of *S. aureus* and *E. coli* on LB solid agar plates were randomly selected and inoculated in 5 mL of LB liquid medium and cultured at 37°C for 12 h under 180 rpm rotation. The next day, 50 μL of the bacterial inoculation was diluted with 4 950 μL of fresh LB medium and cultured at 37°C for 3 h at 180 rpm until OD600 reached 0.8. The bacterial precipitate (1 mL) was obtained by centrifugation at 3 600 rpm for 5 min at 4°C and diluted with H_2O (900 μL). Then, 100 μL of Fe^{2+} (10 μM) was mixed with *S. aureus* (900 μL) and *E. coli* (900 μL) at 37°C for 30 min, respectively. Bacteria were processed for transcriptome analysis. Total RNA extraction, RNA sequencing and bioinformatic data collection were performed by Applied Protein Technology (Shanghai, China).

Lipid ROS measurement

Lipid ROS levels in *S. pullorum* stimulated by ferrous iron were detected using a BODIPY 581/591 C11 fluorescent probe. Single colonies of *S. pullorum* on LB solid agar plates were randomly selected and inoculated in 5 mL of LB liquid medium and cultured at 37°C for 12 h under 180 rpm rotation. The next day, 50 μL of the bacterial inoculation was diluted with 4 950 μL of fresh LB medium and cultured at 37°C for 3 h at 180 rpm until OD600 reached 0.8. The bacterial precipitate (1 mL) was obtained by centrifugation at 3 600 rpm for 5 min at 4°C and diluted with H_2O (900 μL). Then, 100 μL of Fe^{2+} (25, 50, 100, and 200 μM) was mixed with *S. pullorum* (900 μL) at 37°C for 30 min, respectively. The bacterial precipitate was obtained by centrifugation at 3 600 rpm for 5 min at 4°C, after which 200 μL of BODIPY 581/591 C11 probe (2 μM) working solution was added to the bacteria for 30 min of incubation at 37°C. The lipid ROS level was measured by a multi-scan spectrum with excitation at 488 nm and emission at 525 nm.

Bacterial membrane potential measurement

Changes in bacterial membrane potential following ferrous iron treatment were detected using a $DIOC_2(3)$ probe. The *S. pullorum* precipitates (1 mL) at the logarithmic growth stage were obtained by centrifugation at 3 600 rpm for 5 min at 4°C and diluted with H_2O (900 μL). Then, 100 μL of Fe^{2+} (25, 50, 100, and 200 μM) was mixed with *S. pullorum* (900 μL) at 37°C for 30 min, respectively. The bacterial precipitate was obtained by centrifugation at 3 600 rpm for 5 min at 4°C, after which 100 μL of 30 μM $DIOC_2(3)$ (US EVERBRIGHT) probe working solution was added to the bacteria for 30 min of incubation at 37°C. Bacterial membrane potential was measured using a multi-scan spectrum with Ex/Em = 480/525 nm (green fluorescence) and Ex/Em

= 530/590 nm (red fluorescence). Red/green fluorescence ratios were calculated using population mean fluorescence intensities for bacteria.

Lactate dehydrogenase (LDH) measurement

Changes in bacterial membrane integrity following ferrous iron treatment were detected using an LDH assay kit (Solarbio). The *S. aureus* and *E. coli* precipitates (1 mL) at the logarithmic growth stage were obtained by centrifugation at 3 600 rpm for 5 min and diluted with H_2O (450 µL). Then, 50 µL of Fe^{2+} (25, 50, and 200 µM) was mixed with *S. aureus* (450 µL) and *E. coli* (450 µL) at 37°C for 30 min, respectively. The supernatant was collected by centrifugation at 3 600 rpm for 5 min at 4°C. The LDH activity in the supernatant was then measured using an LDH assay kit (Solarbio).

Bacterial live/dead staining assays

The *S. aureus* and *E. coli* precipitates (1 mL) at the logarithmic growth stage were obtained by centrifugation at 3 600 rpm for 5 min at 4°C and diluted with H_2O (900 µL). Then, 100 µL of Fe^{2+} (25, 50, 100, and 200 µM) was mixed with *S. aureus* (900 µL) and *E. coli* (900 µL) at 37°C for 30 min, respectively. The bacterial precipitate was obtained by centrifugation at 3 600 rpm for 5 min at 4°C, after which 1 mL of 5 µM SYTO 9 (Invitrogen) working solution was added to the bacteria for 30 min of incubation at 37°C. After washing twice with PBS, 1 mL of 10 µM propidium iodide (PI) (Beyotime Biotechnology) working solution was added to the bacteria for 30 min of incubation at 37°C. After washing twice with PBS, DAPI (Beyotime Biotechnology) was used to stain the nuclei. After washing, live and dead bacteria after ferrous treatment were observed using a Zeiss confocal microscope (Zeiss LSM980).

Isolation and culture of bone marrow-derived macrophages (BMMs)

Six-week-old male Balb/C mice (obtained from the Laboratory of Immunodeficient Animals, Institute of Biophysics, Chinese Academy of Sciences) were sacrificed by cervical dislocation, immersed in 75% alcohol for 3 min, and then soaked in PBS containing 2% penicillin/streptomycin (Sangon Biotech) for 3 min. The femur and tibia were taken aseptically and immersed in 10 mL of RPMI 1640 medium containing 2% penicillin/streptomycin. Bone marrow was flushed into a sterile 15-mL centrifuge tube with a 1-mL syringe drawn from the 1640 medium and then centrifuged at 1 500 rpm for 10 min at 4°C. The supernatant was discarded and 5 mL of fresh 1640 medium was added to resuspend the cells, followed by centrifugation at 1 500 rpm for 10 min at 4°C. The supernatant was discarded, and the cells were resuspended in 10 mL of complete culture medium (RPMI 1640 + 20% FBS + 1% penicillin/streptomycin + 20 ng/mL recombinant mouse granulocyte macrophage colony stimulating factor (rmGM-CSF)), inoculated into cell culture dishes (100 mm), and incubated in an incubator at 37°C with 5% CO_2 for 3 d, with the culture medium changed. The cells were continuously cultured until day 5, with all semi-adherent cells, i.e., enriched mouse BMMs, then collected. The BMMs were labelled with a FITC-CD 11b fluorescent probe (Invitrogen) for 30 min at 4°C and the purity of the isolated cells was assayed by flow cytometry (BD FACSCalibur).

Detection of intracellular bactericidal effects of ferroptosis inducers and inhibitors

RAW264.7 cells were inoculated in 6-well plates containing 1 mL of complete culture solution

(RPMI 1640 + 20% FBS + 1% penicillin/streptomycin). At the logarithmic growth phase, the RAW264.7 cells were infected with *S. pullorum*, and *Salmonella enteritidis* (SC070) (*S. enteritidis*) at MOI = 20:1 for 1 h, respectively, and extracellular bacteria were killed with gentamicin (200 µg/mL) for 3 h. After washing three times with PBS, the cells were cultured at 37°C under 5% CO_2 in 900 µL of RPMI 1640 containing 1% FBS, with 100 µL of ferroptosis inducers (Fe^{2+} (12.5, 25, and 50 µM), (RSL3 (2, 5, and 10 µM), SSZ (2, 5, and 10 µM), and APAP (2, 5, and 10 µM)) or inhibitors (Fer-1 (2, 5, and 10 µM), EDTA (10, 20, and 50 µM), and DFO (12.5, 25, and 50 µM)) added to the cell culture medium for 6 h (RSL3 incubated for 3 h). A sample of the cells was lysed using 1 mL of buffer containing 0.4% Triton X-100 for 30 min. The number of viable bacteria was counted by plating serial dilutions of bacteria on LB solid medium. Cell activity after bacterial infection and treatment with ferroptosis inducers and inhibitors was assayed with a Cell Counting Kit-8 (CCK-8) (DOJINDO).

RAW264.7 cells were infected with *S. aureus* and *E. coli* at MOI = 20:1 for 1 h, respectively, and extracellular bacteria were killed with gentamicin (200 µg/mL) for 3 h. After washing three times with PBS, the cells were cultured at 37°C under 5% CO_2 in 900 µL of RPMI 1640 containing 1% FBS, with 100 µL of ferroptosis inducers (Fe^{2+} (50µM), RSL3 (5 µM), SSZ (10 µM), and APAP (10 µM)) or inhibitors (Fer-1 (10 µM), EDTA (50 µM), and DFO (50 µM)) added to the cell culture medium for 6 h. TEM was used to observe the bacteria in the cells. Host cell morphological images with different treatments were captured using a Zeiss LSM980confocal microscope.

The BMMs were inoculated in 6-well plates containing 1 mL of complete culture solution (RPMI 1640 + 20% FBS + 1% penicillin/streptomycin). At the logarithmic growth phase, RAW264.7 cells were infected with *S. aureus*, *E. coli*, and *S. pullorum* at MOI = 20:1 for 1 h, respectively, and extracellular bacteria were killed with gentamicin (200 µg/mL) for 3 h. After washing three times with PBS, the cells were cultured at 37°C under 5% CO_2 in 900 µL of RPMI 1640 containing 1% FBS, with 100 µL of ferroptosis inducers (RSL3 (2, 5, and 10 µM), SSZ (2, 5, and 10 µM), and APAP (2, 5, and 10 µM)) or inhibitors (Fer-1 (0.25, 0.5, and 1 µM) and DFO (12.5, 25, and 50 µM)) added to the cell culture medium at 6 h. Cell activity after bacterial infection and treatment with ferroptosis inducers and inhibitors was assayed with a Cell Counting Kit-8 (CCK-8) (DOJINDO) and Annexin V-FITC Apoptosis Detection Kit (Beyotime Biotechnology).

Detection of intracellular bactericidal effects of ferric iron

RAW264.7 cells were inoculated in 6-well plates containing 1 mL of complete culture solution (RPMI 1640 + 20% FBS + 1% penicillin/streptomycin). At the logarithmic growth phase, RAW264.7 cells were infected with *S. aureus*, *E. coli*, and *S. pullorum* at MOI = 20:1 for 1 h, respectively, and extracellular bacteria were killed with gentamicin (200 µg/mL) for 3 h. After washing three times with PBS, the cells were cultured at 37°C under 5% CO_2 in 900 µL of RPMI 1640 containing 1% FBS, with 100 µL of Fe^{3+} (12.5, 25, and 50 µM) then added to the cell culture medium for 6 h. A sample of the cells was lysed using 1 mL of buffer containing 0.4% Triton-X for 30 min. The number of viable bacteria was counted by plating serial dilutions of bacteria on LB solid medium. Intracellular lipid ROS levels were detected using the BODIPY581/591 C11 fluorescent probe after incubation of ferric iron with bacteria-infected macrophages.

Co-localization of FPN with intracellular bacteria

RAW264.7 cells at the logarithmic growth phase were collected and inoculated into 24-well cell culture plates with round coverslip (Biosharp) placed in advance, followed by the addition of 500 µL of complete culture medium. Cells were then cultured to 80% confluence at 37°C and 5% CO_2. Individual colonies of BL21 (*E. coli* strain with green fluorescence) on LB solid plates were randomly selected and inoculated in 5 mL of LB liquid medium containing kanamycin (50 µg/mL), then incubated for 12 h at 37°C with 180 rpm rotation. The following day, 50 µL of the bacterial inoculation was diluted with 4 950 µL of fresh LB medium containing 50 µg/mL kanamycin and cultured at 37°C for 3 h under 180 rpm until OD600 reached 0.8. After this, 0.5 mM isopropyl thiogalactoside (IPTG) (GenStar) was added to the bacterial solution to induce 3 h. RAW264.7 cells were infected with BL21 at MOI = 20:1 after IPTG induction for 1 h and extracellular bacteria were killed with gentamicin (200 µg/mL) for 3 h. After washing three times with PBS, the cells were cultured at 37°C under 5% CO_2 in 0.5 mL of RPMI 1640 containing 1% FBS for 1, 3, 6, 12, and 24 h. The cells were then rinsed three times with PBS, fixed with 4% paraformaldehyde for 30 min, saturated with 0.4% Triton X-100 for 5 min, and blocked with 5% albumin bovine V (BSA) for 1 h. Cells were then incubated at 4°C for 12 h with 200 µL of FPN primary antibodies (1:200; Thermo Fisher), followed by incubation with 200 µL of fluorescein Alexa Fluor 647-conjugated goat anti-rabbit IgG (1:200; BIORIGIN) at 37°C for 1.5 h, and staining with DAPI (Beyotime Biotechnology) for 5 min. After washing, the co-localization of FPN with BL21 was observed using a Zeiss confocal microscope (Zeiss LSM980).

Co-localization of ferrous iron with intracellular bacteria

RAW264.7 cells at the logarithmic growth phase were collected and inoculated into 24-well cell culture plates with round coverslip (Biosharp) placed in advance, followed by the addition of 500 µL of complete culture medium. Cells were then cultured to 80% confluence at 37°C and 5% CO_2. Individual colonies of BL21 (with green fluorescence) on LB solid plates were randomly selected and inoculated in 5 mL of LB liquid medium containing kanamycin (50 µg/mL) and incubated for 12 h at 37°C with 180 rpm rotation. The following day, 50 µL of the bacterial inoculation was diluted with 4 950 µL of fresh LB medium containing 50 µg/mL kanamycin and cultured at 37°C for 3 h under 180 rpm until OD600 reached 0.8. Then, 0.5 mM IPTG (GenStar) was added to the bacterial solution for induction for 3 h. The bacterial precipitate was obtained by centrifugation at 3 600 rpm for 5 min at 4°C and diluted to 1×10^8 CFUs/mL with 5 mL of RPMI 1640 culture medium. The RAW264.7 cells were infected with 50 µL of BL21 bacterial solution at MOI = 20:1 for 1 h, and extracellular bacteria were killed with gentamicin (200 µg/mL) for 3 h. After washing three times with PBS, the cells were cultured at 37°C under 5% CO_2 in 0.5 mL of RPMI 1640 containing 1% FBS for 6, 12, and 24 h. The cells were then washed three times with PBS. Intracellular ferrous iron was labelled with a FeRhoNox™-1 fluorescent probe (GORYO Chemical) at 37°C for 45 min. DAPI was used to stain the nuclei. After washing, the co-localization of ferrous iron with BL21 was observed using a Zeiss confocal microscope (Zeiss LSM980).

Ferritin and transferrin receptor fluorescence intensity measurement

RAW264.7 cells were inoculated in 24-well cell culture plates with round coverslip placed in advance. At the logarithmic growth phase, the RAW264.7 cells were infected with *E. coli* at MOI

= 20:1 for 1 h, and extracellular bacteria were killed with gentamicin (200 μg/mL) for 3 h. After washing three times with PBS, the cells were cultured at 37℃ under 5% CO_2 in 0.5 mL of RPMI 1640 containing 1% FBS for 1, 3, 6, 12, and 24 h. Cells were washed three times with PBS, fixed with 4% paraformaldehyde for 30 min, saturated with 0.4% Triton X-100 for 5 min, and blocked with 5% albumin bovine V (BSA) for 1 h. Cells were then incubated at 4℃ for 12 h with 200 μL of ferritin or transferrin receptor primary antibodies (1:200; Abcam), followed by incubation with 200 μL of fluorescein Alexa Fluor 488-conjugated goat anti-rabbit IgG (1:200; BIORIGIN) at 37℃ for 1.5 h, and staining with DAPI (Beyotime Biotechnology) for 5 min. After washing, the fluorescence intensity of ferritin and transferrin receptor was observed using a Zeiss confocal microscope (Zeiss LSM980).

Intracellular bactericidal assay of RAW264.7 macrophages after FPN knockdown

Ferroportin shRNA (MSH031547-LVRU6P-b/c, Matrix) and null shRNA (CSHC7R001-1-LVRU6P, Matrix) were transfected into 293T cells. The viral supernatant was collected 48 h after transfection and filtered through a 0.22-μM filter. The RAW264.7 macrophages were inoculated in 6-well plates containing 1 mL of complete culture solution (RPMI 1640 + 20% FBS + 1% penicillin/streptomycin) and infected with 1 mL of the filtered virus for 2 h, then replenished with 2 mL of fresh RPMI 1640 culture medium (RPMI 1640 + 1% FBS + 1% penicillin/streptomycin) and incubated for 24 h at 37℃. The medium was replaced with fresh RPMI 1640 culture medium (RPMI 1640 + 1% FBS + 1% penicillin/streptomycin), followed by an additional 48 h of culture at 37℃. After 48 h of virus infection, 2 mL of 2.5 μg/mL puromycin (GenStar) was added to the culture medium for screening. Fresh culture medium was replaced every 2 days and the cells were incubated continuously for more than a week at 37℃ under 5% CO_2. After washing with ice-cold PBS, the cells were collected by centrifugation at 2 000 rpm for 5 min at 4℃ and lysed in 100 μL of RIPA buffer (Beyotime Biotechnology) containing protease inhibitor. The lysate was centrifuged at 12 000 g for 10 min at 4℃ and the protein concentrations were determined and calibrated using a BCA Protein Assay Kit (Thermo Fisher). Protein extracts were resolved on sodium dodecyl sulfate (SDS)-polyacrylamide gels, transferred to polyvinylidene fluoride (PVDF) membranes, and blocked for 1 h with Tris-buffered saline (TBS) containing 0.05% Tween 20 (TBST) and 5% skimmed milk powder. The PVDF membranes were washed three times with TBST (10 min each time). Ferroportin antibodies (Thermo Fisher) were diluted 1:1 000 in TBST (containing 5% bovine serum albumin) and incubated overnight at 4℃. After washing with TBST, membranes were incubated with horseradish peroxidase (HRP)-conjugated goat anti-rabbit antibodies (Sigma) in a solution of TBST. Protein bands were visualized using a fully automated chemiluminescence image analysis system (Tanon-4600SF).

The RAW264.7 cells were inoculated into a 6-well plate containing 1 mL of complete culture medium (RPMI 1640 + 20% FBS + 1% penicillin/streptomycin). The RAW264.7 cells were infected with *S. aureus*, *E. coli*, and *S. pullorum* at MOI = 20:1 after FPN knockdown for 1 h, respectively, and extracellular bacteria were killed with gentamicin (200 μg/mL) for 3 h. After washing three times with PBS, the cells were cultured at 37℃ under 5% CO_2 in 1 mL of RPMI 1640 containing 1% FBS for 3 h. A sample of the cells was lysed using buffer containing 0.4% Triton X-100 for 30 min. The number of viable bacteria was counted by plating serial dilutions of

bacteria on LB solid medium.

Hepcidin bactericidal assay for intracellular bacteria

Hepcidin (100 μL; 2, 4, and 6 μg/mL) was added to RAW264.7 cells cultured in 6-well plates for 3 h of incubation at 37°C. The pre-treated RAW264.7 cells were then infected with *S. aureus*, *E. coli*, and *S. pullorum* at MOI = 20:1 for 1 h, respectively, and extracellular bacteria were killed with gentamicin (200 μg/mL) for 3 h. After washing three times with PBS, the cells were cultured at 37°C under 5% CO_2 in 1 mL of RPMI 1640 containing 1% FBS for 3 h. RAW264.7 cells cultured in 6-well plates were infected with *S. aureus*, *E. coli*, and *S. pullorum* at MOI = 20:1 for 1 h, respectively, and extracellular bacteria were killed with gentamicin (200 μg/mL) for 3 h. After washing three times with PBS, the cells were cultured at 37°C under 5% CO_2 in 1 mL of RPMI 1640 containing 1% FBS and hepcidin (2, 4, and 6 μg/mL) for 3 h. A sample of the cells was lysed using buffer containing 0.4% Triton X-100 for 30 min. The number of viable bacteria was counted by plating serial dilutions of bacteria on LB solid medium. Immunoblot assay was used to detect the effect of hepcidin on FPN. Intracellular ferrous iron was detected with a FeRhoNox^{TM}-1 fluorescent probe (GORYO Chemical), with 200 μL of 5 μM FeRhoNox^{TM}-1 working solution then added to the cells for 30 min of incubation at 37°C. The cells were detected by flow cytometry (BD FACSCalibur).

***In vivo* planktonic tail vein infection**

Six-week-old male Balb/C mice (obtained from the Laboratory of Immunodeficient Animals, Institute of Biophysics, Chinese Academy of Sciences) were used to establish *in vivo* infection models. The mice (five in each group) were infected in the tail vein with *S. aureus* (1×10^6 CFUs) or *S. pullorum* (1×10^6 CFUs) at the logarithmic growth stage. After 12 h of infection, blood was collected from the orbital vein and erythrocytes were lysed in erythrocyte lysate (1 mL) (TIANGEN) at room temperature. The mixture was centrifuged at 3 500 rpm for 10 min at 4°C to remove the erythrocyte lysate. The cells were then divided equally into two portions. One portion of cells was labelled with 200 μL of BODIPY 581/591 C11 (2 μM), followed by incubation for 30 min at 37°C. After washing with PBS, 100 μL of APC CD 11b antibody (Invitrogen) working solution (1:1 000) was added to the cells, followed by 30 min of incubation at 4°C. The cells were then washed three times with PBS, and changes in lipid ROS in peripheral blood macrophages were detected by flow cytometry.

The other cells were labelled with 200 μL of FeRhoNox^{TM}-1 probes (5 μM), followed by incubation for 45 min at 37°C. After washing with PBS, 100 μL of APC CD 11b antibody (Invitrogen) working solution (1:1 000) was added to the cells, followed by 30 min of incubation at 4°C. The cells were then washed three times with PBS, and changes in ferrous iron in peripheral blood macrophages were detected by flow cytometry.

After 12 h of infection, 100 μL of SSZ (10, 50, and 100 μM) or DFO (10, 50, and 100 μM) was administered intravenously twice a day, with a total of five treatments per mouse. Control mice with bacterial infection received 100 μL of PBS only via intravenous injection. Body weight and behavior, including feeding, drinking, and activity, were continuously observed and recorded for 3 days. All mice were sacrificed on day 3 after infection, and their hearts, livers, lungs, spleens, and

kidneys were collected in 5 mL of PBS. Tissue samples were ground under aseptic conditions. Viability of bacteria was assessed by calculating bacterial number (CFUs/mouse) in organs. Antibacterial efficiency was determined by calculating the ratio of bacterial number between the SSZ or DFO-treated groups and PBS-treated group.

After 12 h of infection, 100 μL of SSZ (100 μM) or DFO (100 μM) was administered intravenously twice a day, with a total of five treatments per mouse. Control mice with bacterial infection received 100 μL of PBS only via intravenous injection. All mice were sacrificed on day 3 after infection. Their main organs (i.e., liver, kidneys, spleen, lungs, and heart) were collected in 10 mL of 4% paraformaldehyde, then fixed in paraffin and stained with hematoxylin-eosin (H&E) to examine changes in organ histomorphology.

After 12 h of infection, 100 μL of SSZ (100 μM) or DFO (100 μM) was administered intravenously twice a day, with a total of five treatments per mouse. Control mice with bacterial infection received 100 μL of PBS only via intravenous injection. All mice were sacrificed on day 3 after infection, and blood was collected from the orbital vein and erythrocytes were lysed in erythrocyte lysate (1 mL) (TIANGEN) at room temperature. The mixture was centrifuged at 3 500 rpm for 10 min at 4°C to remove the erythrocyte lysate. The cells were then divided equally into two portions. One portion of cells was labelled with 200 μL of BODIPY 581/591 C11 (2 μM), followed by incubation for 30 min at 37°C. After washing with PBS, 100 μL of APC CD 11b antibody (Invitrogen) working solution (1:1 000) was added to the cells, followed by 30 min of incubation at 4°C. The cells were then washed three times with PBS, and changes in lipid ROS in peripheral blood macrophages were detected by flow cytometry. The other cells were labelled with 200 μL of FeRhoNox^{TM}-1 probes (5 μM), followed by incubation for 45 min at 37°C. After washing with PBS, 100 μL of APC CD 11b antibody (Invitrogen) working solution (1:1 000) was added to the cells, followed by 30 min of incubation at 4°C. The cells were then washed three times with PBS, and changes in ferrous iron in peripheral blood macrophages were detected by flow cytometry.

Evaluation of antibacterial efficacy of SSZ and DFO with intravenous intracellular bacterial infections

Six-week-old male Balb/C mice (obtained from the Laboratory of Immunodeficient Animals, Institute of Biophysics, Chinese Academy of Sciences) were used to establish *in vivo* infection models. Mouse BMMs were infected with *S. aureus* or *S. pullorum* following the *in vitro* intracellular bacterial model. Following infection, cells were collected from a single well of a 6-well plate, before being resuspended in 1 mL of PBS, after which 100 μL (10^5 cells) of the mixture was intravenously injected into each mouse. After 12 h of infection, 100 μL of SSZ (100 μM) or DFO (100 μM) was administered intravenously twice a day, with a total of five treatments per mouse. Control mice with bacterial infection received 100 μL of PBS only via intravenous injection. Body weight and behavior, including feeding, drinking, and activity, were continuously observed and recorded for 3 days.

Biosafety evaluation of SSZ or DFO

Six-week-old male Balb/C mice were used to establish *in vivo* infection models. Fifteen healthy

Balb/C mice were randomly divided into three groups (five in each group). Two groups were intravenously injected with 100 μL of SSZ (100 μM) and DFO (100 μM) twice a day, respectively. One group was intravenously injected with PBS as the control. On day 3, the injections were stopped, and the mice were housed until day 7. Body weight and behavior, including feeding, drinking, and activity, were continuously observed and recorded for 7 days. All mice were sacrificed on day 7 after infection and blood was collected from the orbital vein to assess the levels of hemoglobin (Hb), mean corpuscular hemoglobin (MCH), mean corpuscular volume (MCV), red blood cells (RBC), hematocrit (HCT), white blood cells (WBC), platelet count (PLT), neutrophilic granulocytes (NEUT). The main organs, i.e., liver, kidneys, spleen, lungs, and heart, were collected in 10 mL of 4% paraformaldehyde, then fixed in paraffin and stained with H&E to examine changes in organ histomorphology.

Statistical analysis and reproducibility

All experiments were performed as biological replicates. Sample size for each experimental group per condition is reported in the corresponding figure legends and Methods section. For bacterial and cell experiments, sample size was not predetermined, and all samples were included in the analysis. In the animal experiments, no statistical methods were used to predetermine sample size (n = number of mice per group), and all animals were used for analysis unless they died. GraphPad Prism v7.0 (GraphPad Software) was used for statistical analyses. For comparing two groups, we used two-tailed Student's *t*-test for column analyses. For comparing multiple groups, we used two-way analysis of variance (ANOVA). NS, not significant ($P > 0.05$); *$P < 0.05$; **$P < 0.01$; ***$P < 0.001$, and ****$P < 0.0001$.

Reference

1. Li QC, Li Y, Xia J, Wang X, Yin KQ, Hu YC, et al. Virulence of *Salmonella enterica* serovar Pullorum isolates compared using cell-based and chicken embryo infection models. Poultry Sci. 2019; 983: 1488-93.
2. Xu ZB, Qiu ZY, Liu Q, Huang YX, Li DD, Shen XG, et al. Converting organosulfur compounds to inorganic polysulfides against resistant bacterial infections. Nat Commun. 2018; 91: 3713.

Supplementary figures

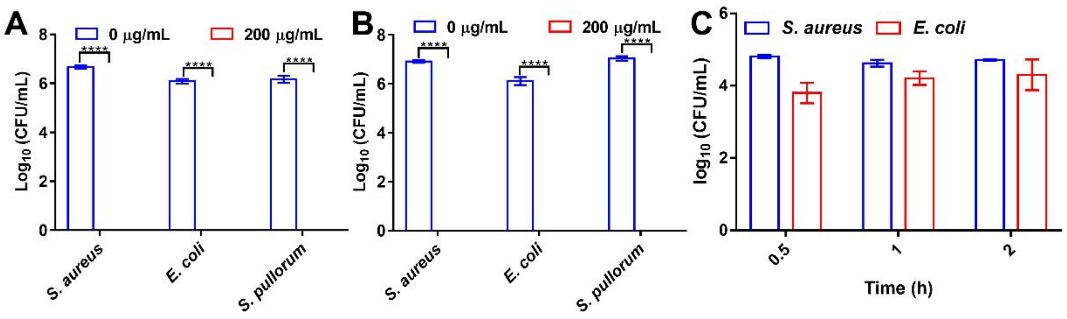

Figure S1. Bacterial inhibition efficiency of gentamicin. (A) Antibacterial activity of gentamicin after treatment for 1 h. **(B)** Antibacterial activity of gentamicin after treatment for 3 h. **(C)** Intracellular bacterial viability in RAW264.7 cells after gentamicin treatment. n = 3, ****p < *0.0001.*

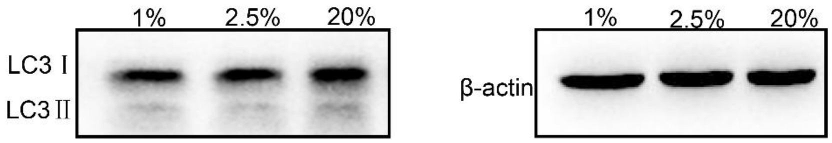

Figure S2. Immunoblotting of LC3 proteins expression in RAW264.7 cells cultured in RPMI 1640 containing different concentration of FBS.

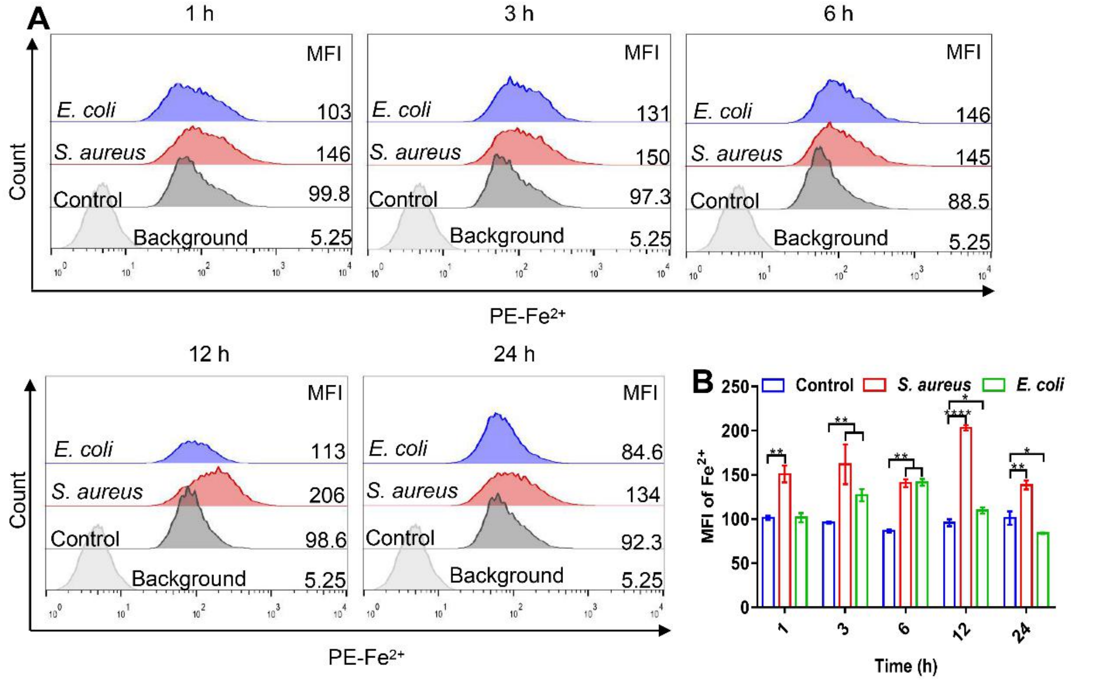

Figure S3. Ferrous iron level in macrophages infected by bacteria. FeRhoNox™-1 probe was used to detect ferrous iron with Flow cytometry. n = 3, *$p < 0.05$, **$p < 0.01$, ****$p < 0.0001$.

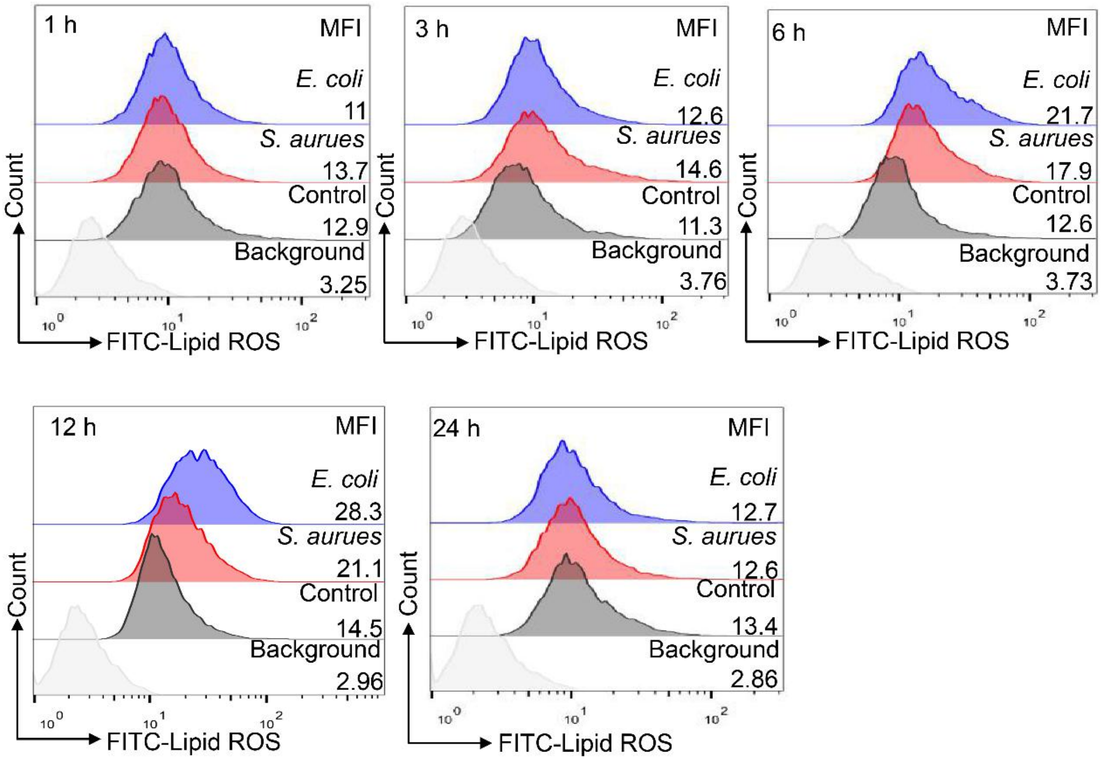

Figure S4. Lipid peroxide level in macrophages infected by bacteria. BODIPY 581/591-C11 probe was used to detect lipid peroxide with Flow cytometry. MFI is the abbreviation of mean

fluorescence intensity.

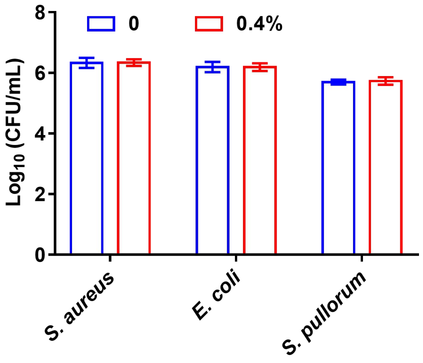

Figure S5. The influence of 0.4% Triton X-100 on bacterial viability.

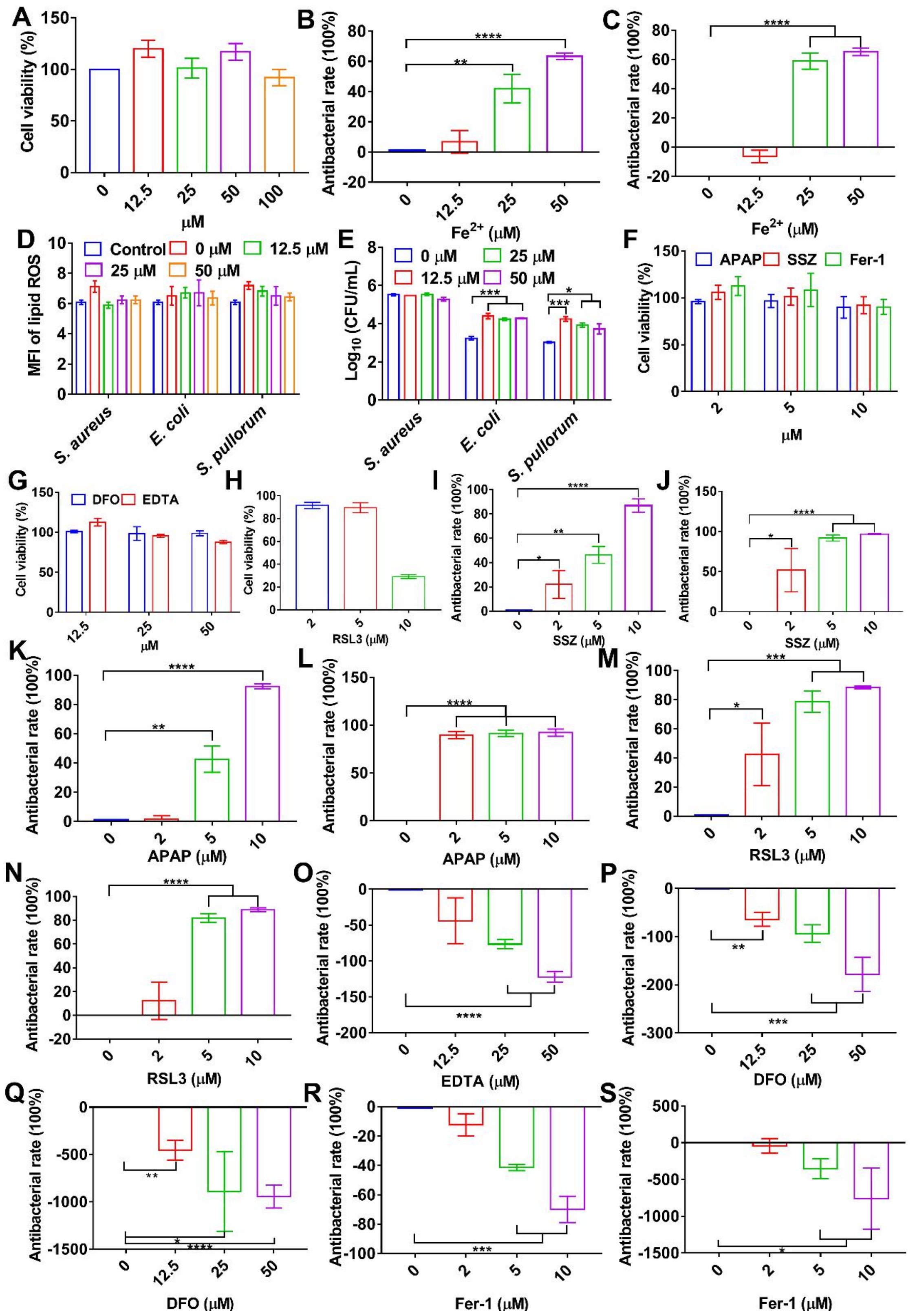

Figure S6. Cellular ferroptotic stress contributes to macrophage defense against intracellular bacteria. (A) CCK8 assay for RAW264.7 cells after 12 h of ferrous treatment. **(B)** Inhibitory effects of ferrous iron on intracellular *S. pullorum*. **(C)** Inhibitory effects of ferrous iron on intracellular *S. enteritidis*. **(D)** Changes of lipid peroxide in cells treated by ferric iron. BODIPY 581/591 C11 probe was used to measure the lipid peroxide by a multi-scan spectrum with

excitation at 488 nm and emission at 525 nm. **(E)** Bactericidal effect of ferric iron on intracellular bacteria. **(F)-(G)** CCK8 assay for RAW246.7 cells activity after APAP, SSZ, Fer-1, DFO, and EDTA treatment for 6 h. **(H)** CCK8 assay for RAW246.7 cells activity after RSL3 treatment for 3 h. **(I)** Bactericidal effect of SSZ on intracellular *S. pullorum*. **(J)** Bactericidal effect of SSZ on intracellular *S. enteritidis*. **(K)** Bactericidal effect of APAP on intracellular *S. pullorum*. **(L)** Bactericidal effect of APAP on intracellular *S. enteritidis*. **(M)** Bactericidal effect of RSL3 on intracellular *S. pullorum*. **(N)** Bactericidal effect of RSL3 on intracellular *S. enteritidis*. **(O)** Bactericidal effect of EDTA on intracellular *S. pullorum*. **(P)** Bactericidal effect of DFO on intracellular *S. pullorum*. **(Q)** Bactericidal effect of DFO on intracellular *S. enteritidis*. **(R)** Bactericidal effect of Fer-1 on intracellular *S. pullorum*. **(S)** Bactericidal effect of Fer-1 on intracellular *S. enteritidis*. n = 3, $^*p < 0.05$, $^{**}p < 0.01$, $^{***}p < 0.001$, $^{****}p < 0.0001$.

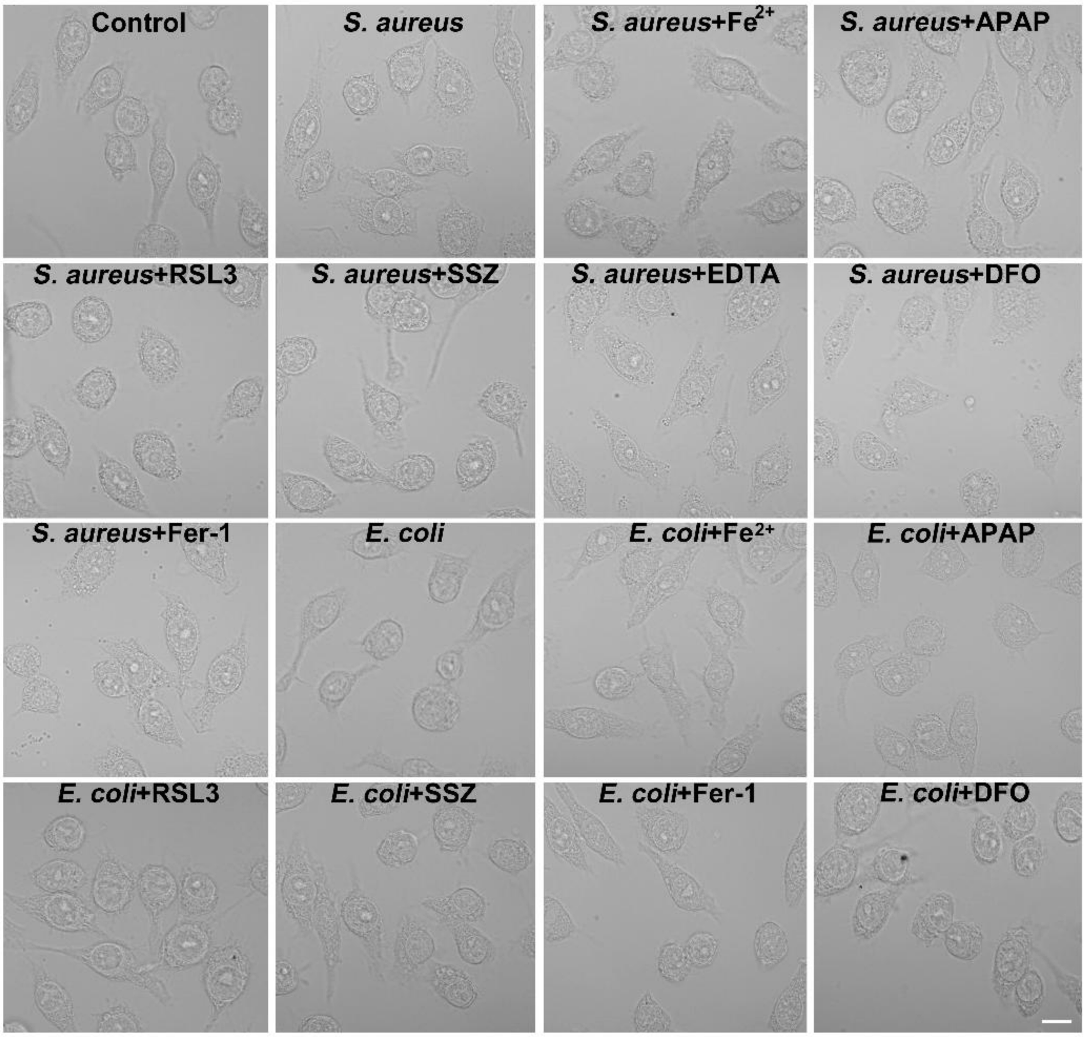

Figure S7. Images of RAW264.7 cell morphology captured by LSM980 laser confocal microscopy (Brightfield). Scale bar = 10 μm.

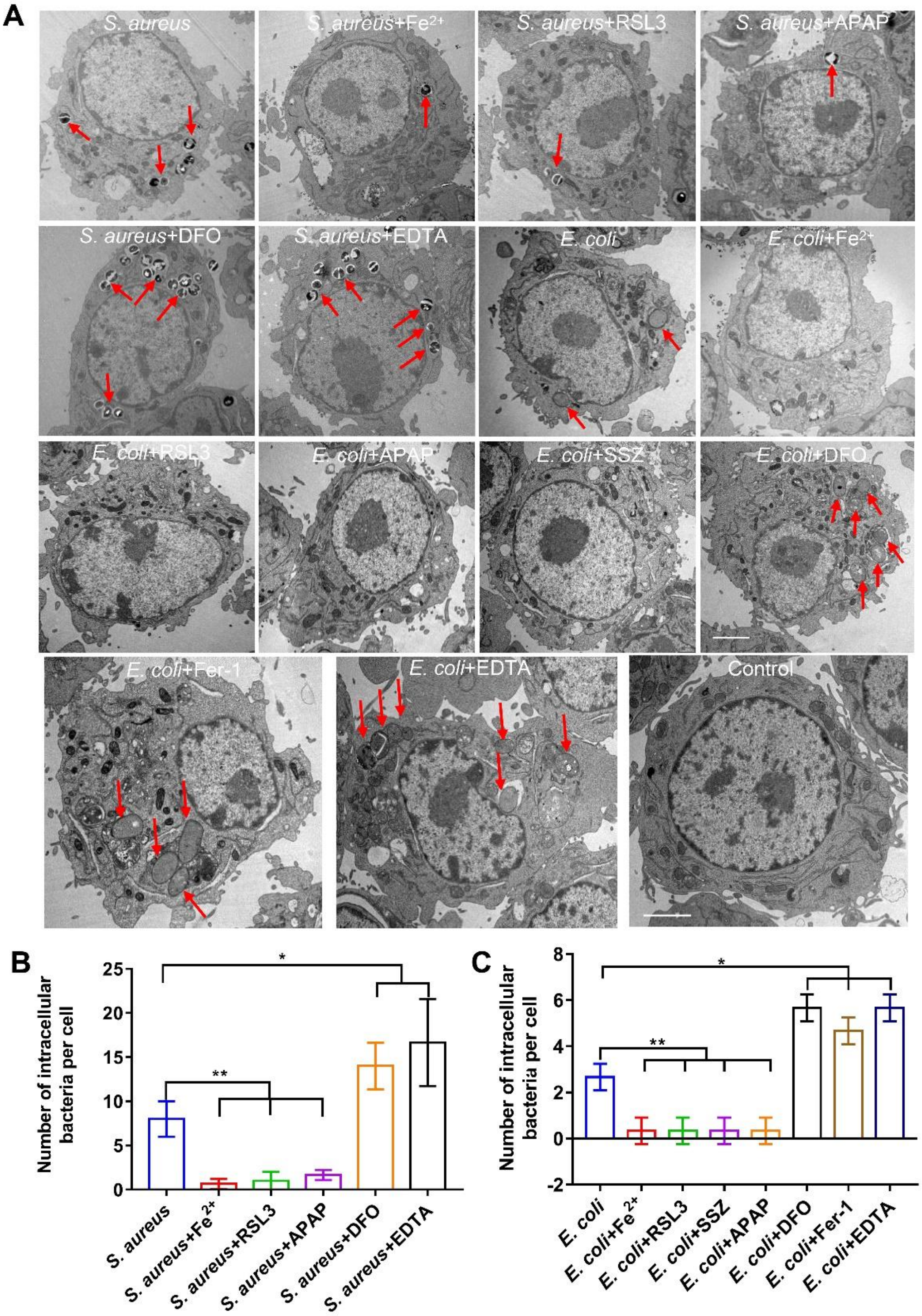

Figure S8. TEM characterization of intracellular *S. aureus* and *E. coli* treated with ferroptosis inducers or inhibitors. (A) Host cell and intracellular bacteria morphology. Scale bar = 2 μm. **(B)** The comparison of the number of intracellular *S. aureus* between groups in (**A**) based on TEM characterizations. **(C)** The comparison of the number of intracellular *E. coli* between groups in (**A**) based on TEM characterizations. The bacteria and cells were counted from TEM images and normalized to number of intracellular bacteria per cell (mean ± SD). n = 3, *$p < 0.05$, **$p < 0.01$.

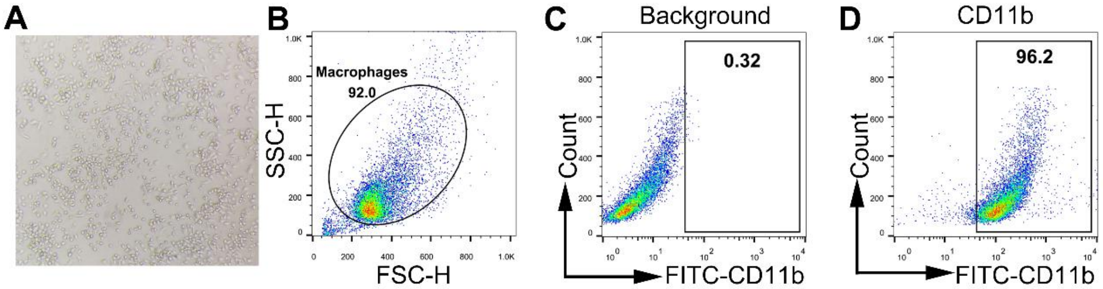

Figure S9. Characterization of the morphology and purity of bone marrow proto-macrophages (BMMs). (A) Characterization of BMMs. **(B)-(D)** BMMs purity determination. BMMs were labelled with a CD 11b fluorescent probe and the purity of the isolated cells was assayed by flow cytometry.

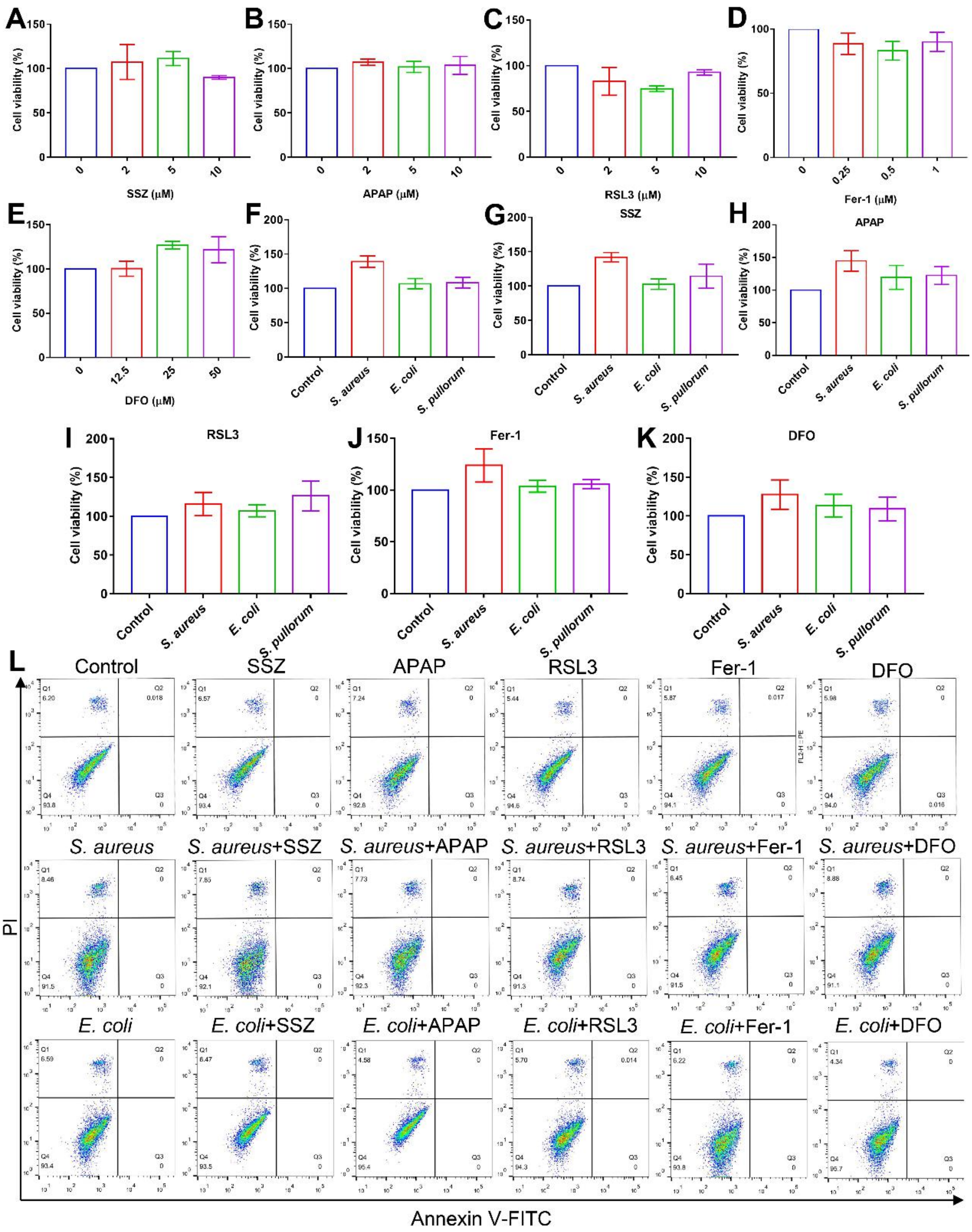

Figure S10. BMM cells viability under ferroptotic stress. (A)-(E) CCK8 assay for BMM cells viability after ferroptosis inducers or inhibitors treatment for 6 h. **(F)** CCK8 assay for BMM cells viability after bacterial infection for 6 h. **(G)-(K)** CCK8 assay for BMM cells viability after bacterial infection and treatment with SSZ (10 μM), APAP (10 μM), RSL3 (10 μM), Fer-1 (1 μM), and DFO (50 μM) for 6 h, respectively. **(L)** Apoptosis assay for BMM cells after bacterial infection and treatment with SSZ (10 μM), APAP (10 μM), RSL3 (10 μM), Fer-1 (1 μM), and DFO (50 μM) for 6 h respectively. n = 3.

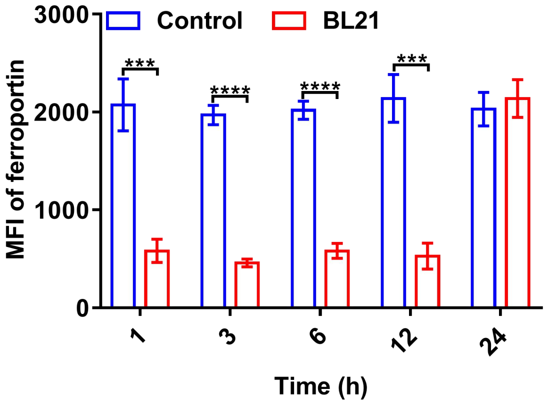

Figure S11. Mean fluorescence intensity of ferroportin measured with a confocal microscope (Zeiss LSM980). n = 3, ***$p < 0.001$, ****$p < 0.0001$.

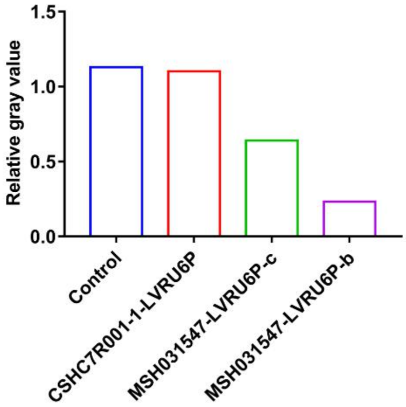

Figure S12. The statistics of relative grey value for knockdown efficiency of ferroportin.

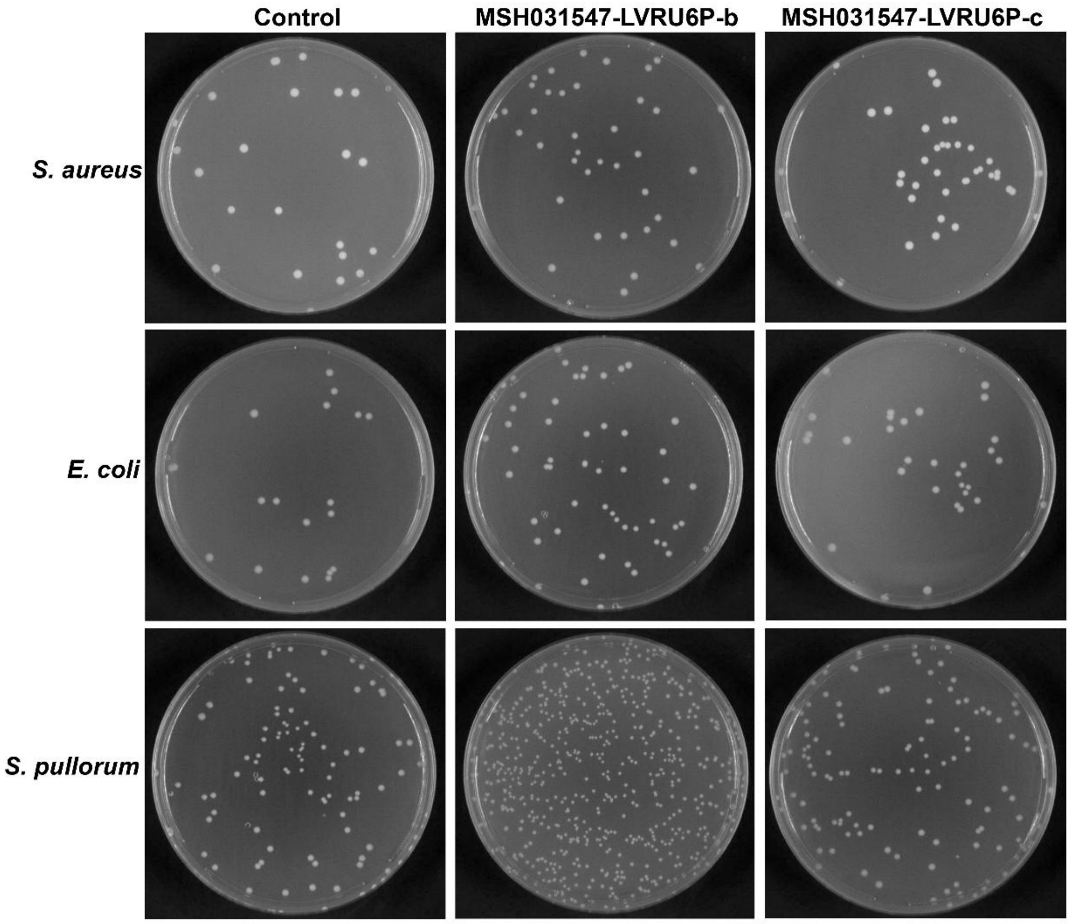

Figure S13. The bacteria in macrophages after ferroportin knockdown identified with LB solid agar plate.

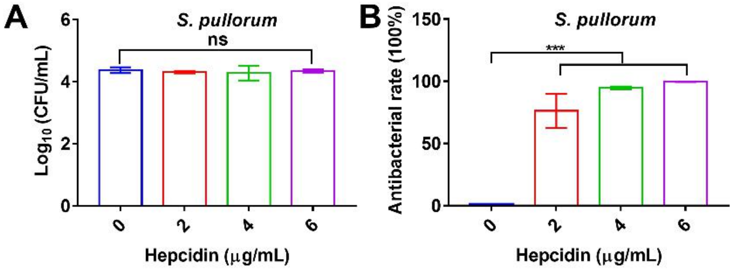

Figure S14. Antibacterial effect of hepcidin on intracellular bacteria. (A) Intracellular *S. pullorum* killing effect of hepcidin pre-treated cells. **(B)** Intracellular *S. pullorum* killing effect of hepcidin post-treatment. n = 3, ns = no significant, ***$p < 0.001$.

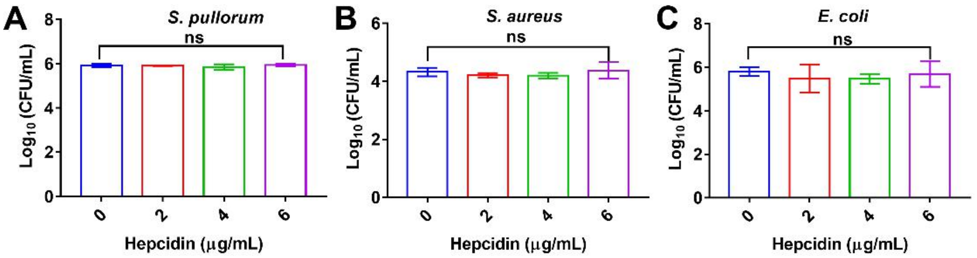

Figure S15. Antibacterial effect of hepcidin on planktonic bacteria. n = 3, ns = no significant.

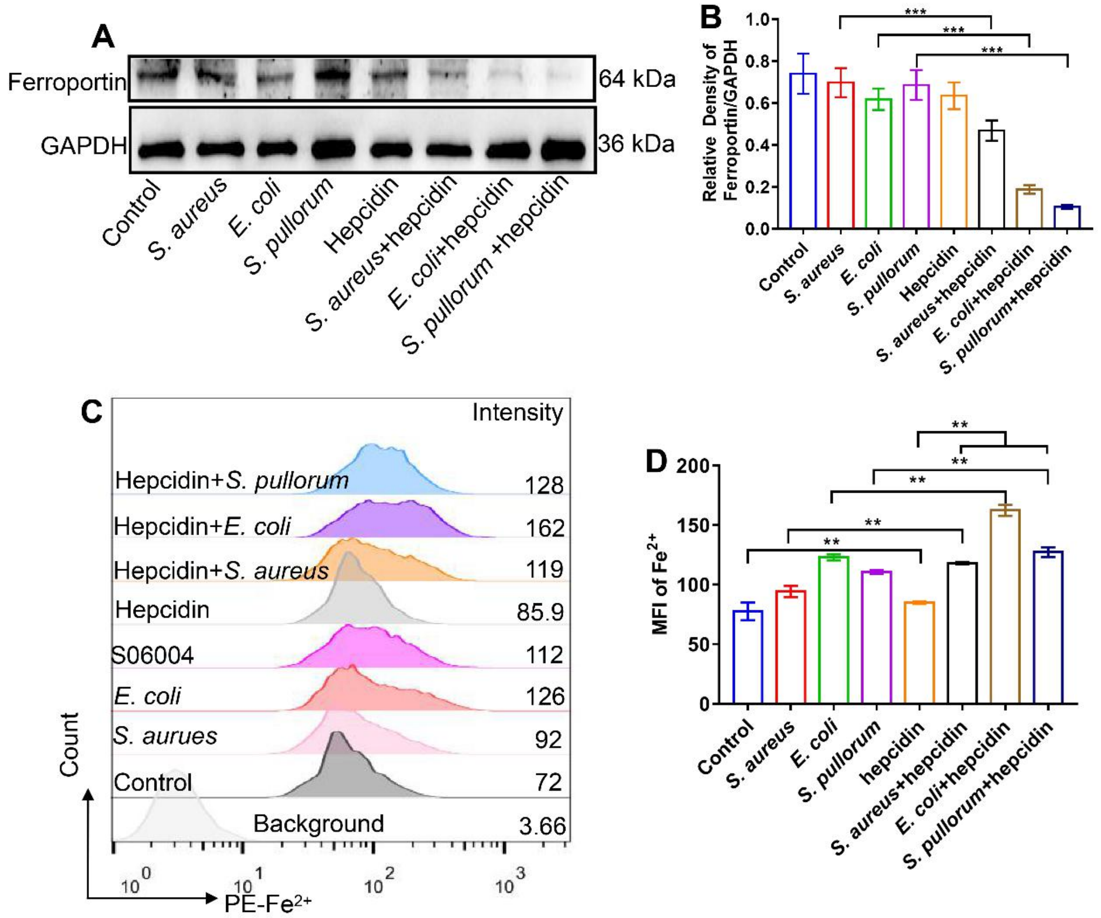

Figure S16. Hepcidin treatment of cells increases intracellular ferrous iron content by degrading ferroportin. (A)-(B) Hepcidin inhibits the expression of ferroportin. **(C)-(D)** Intracellular ferrous iron levels after hepcidin treatment of cells. Intracellular ferrous iron was labelled with FeRhoNox^{TM}-1 fluorescent probes. The cells were detected by flow cytometry (BD FACSCalibur). n = 3, ***p < 0.01*, ****p < 0.001*.

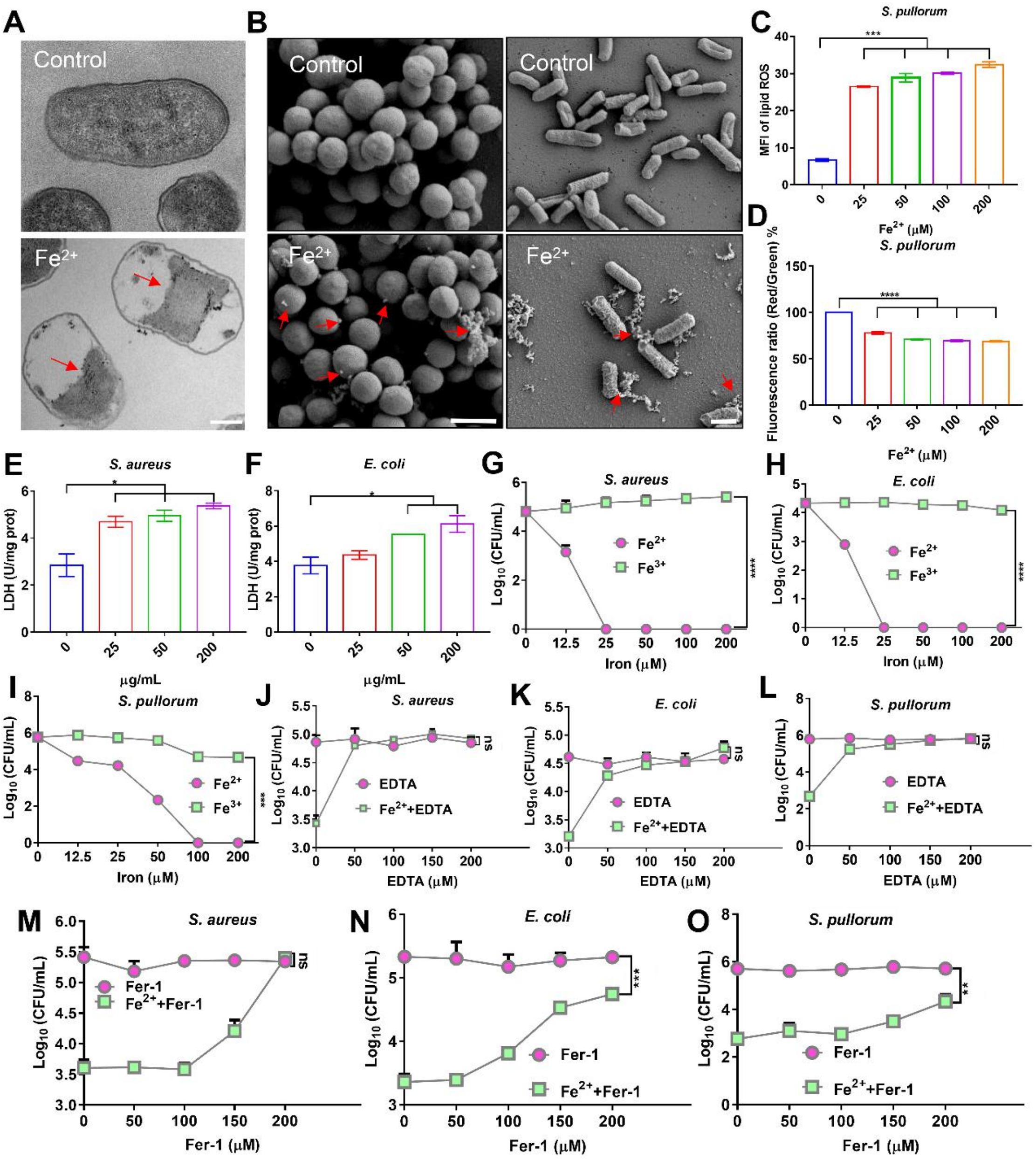

Figure S17. Ferrous iron directly induces bacterial ferroptosis-like death. (A) TEM characterization of *S. pullorum* treated by ferrous iron. Scale bar = 200 nm. **(B)** SEM image of *S. aureus* and *E. coli* morphology by ferrous iron treatment. Scale bar = 1 μm. **(C)** Changes in lipid peroxide in bacteria treated with ferrous iron. BODIPY 581/591 C11 probe was used to measure lipid peroxide by a multi-scan spectrum with excitation at 488 nm and emission at 525 nm. **(D)** Changes in bacterial membrane potential after treatment with ferrous iron. DIOC$_2$(3) probe was used to measure bacterial membrane potential by a multi-scan spectrum with Ex/Em = 480/525 nm (green fluorescence) and Ex/Em = 530/590 nm (red fluorescence). Red/green fluorescence ratios were calculated using population mean fluorescence intensities for bacteria. **(E)** LDH activity assay for *S. aureus* after treatment with ferrous iron. **(F)** LDH activity assay for *E. coli* after treatment with ferrous iron. **(G)-(I)** Antibacterial activity of ferrous iron and ferric iron. **(J)-(O)** EDTA and ferrostatin-1 inhibited bacterial death induced by ferrous iron. n = 3, ns = no significant, **p < 0.05, **p < 0.01, ***p < 0.001.*

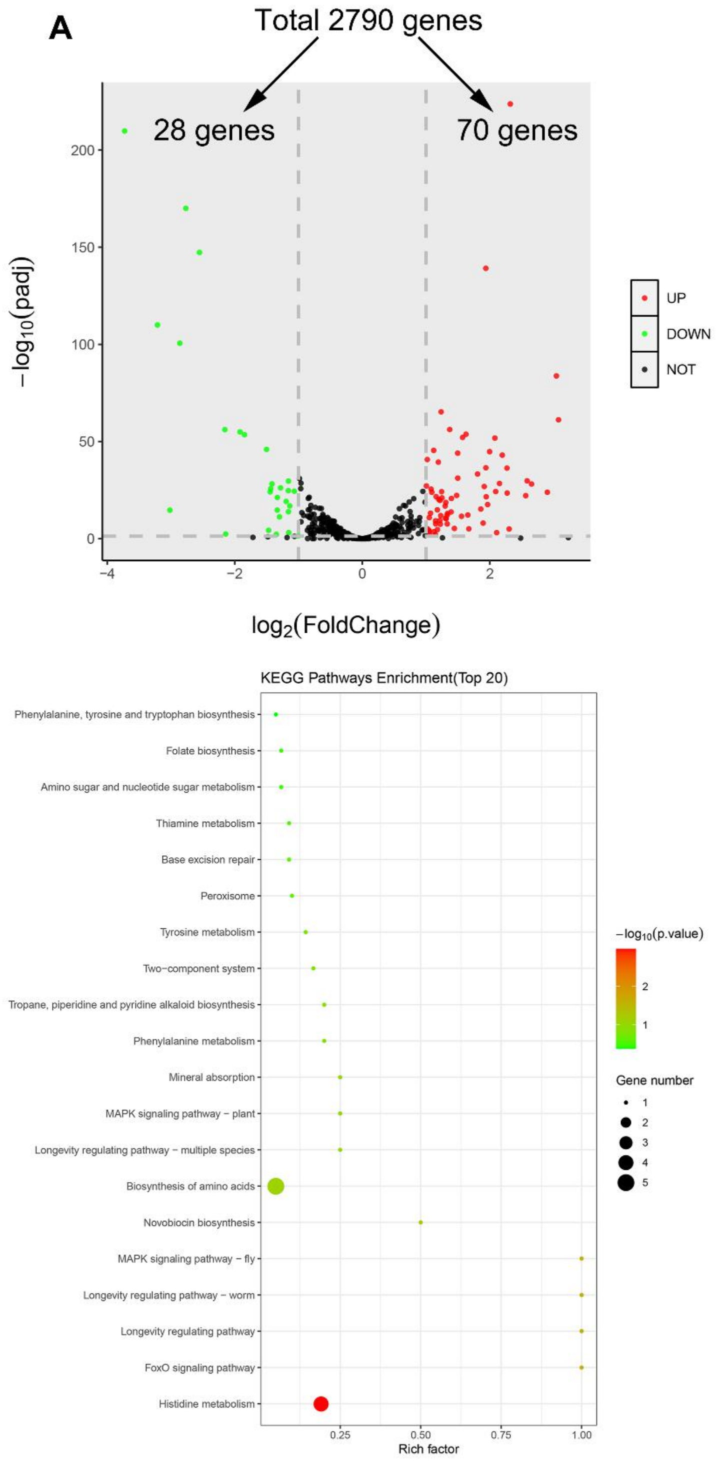

Figure S18. RNA-seq analysis of *S. aureus* treated by ferrous iron (10 μM). (A) Effect of ferrous iron on gene number at the transcriptional level. **(B)** The KEGG pathways affected by ferrous at transcriptional level.

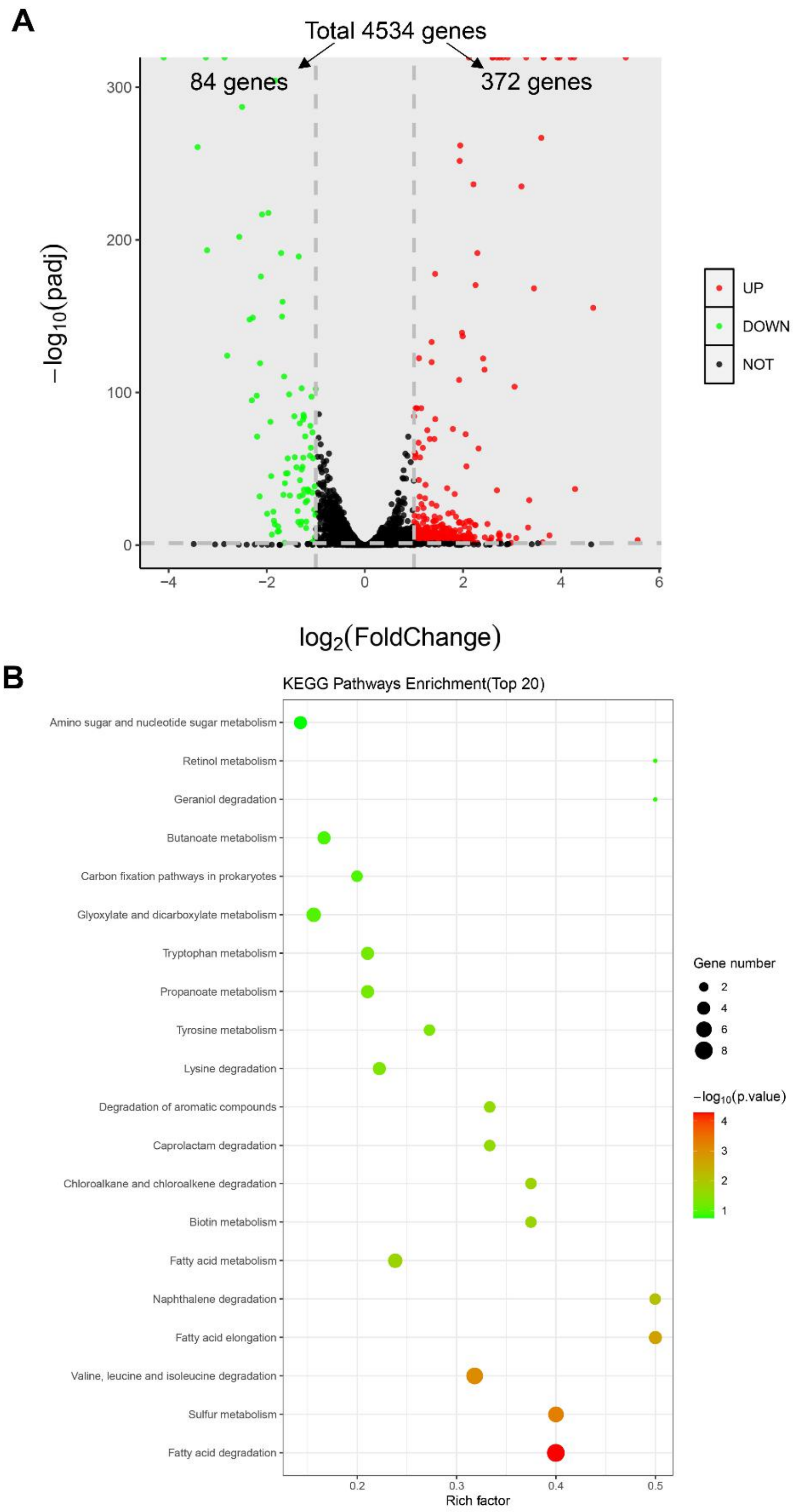

Figure S19. RNA-seq analysis of *E. coli* treated by ferrous iron (10 μM). (A) Effect of ferrous iron on gene number at the transcriptional level. **(B)** The KEGG pathways affected by ferrous at transcriptional level.

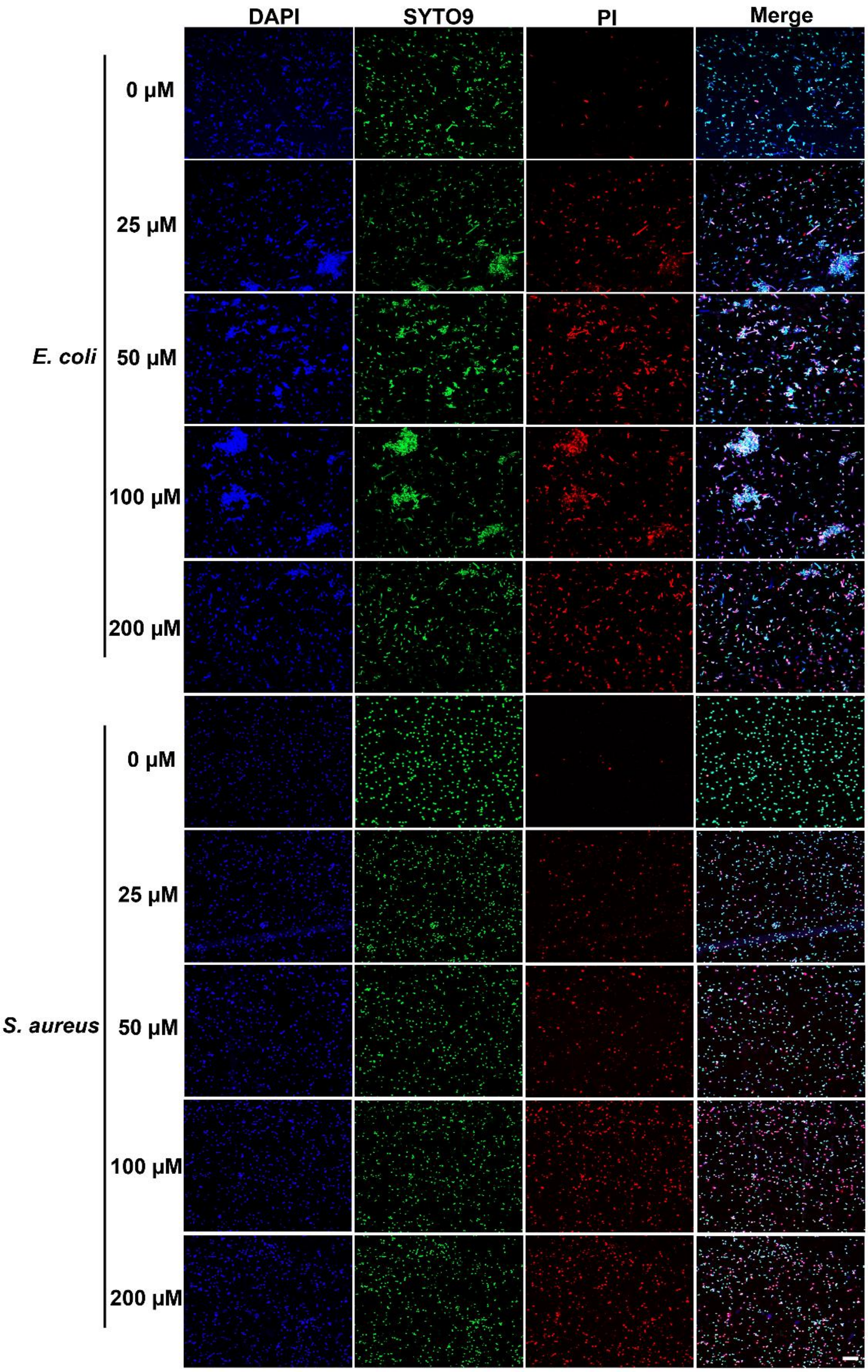

Figure S20. Confocal images of live/dead assay of *S. aureus* and *E. coli* after incubation with ferrous iron. DAPI stained cell nucleus. SYTO 9 stained live/dead *S. aureus* and *E. coli*. PI stained dead *S. aureus* and *E. coli*. Scale bar = 10 μm.

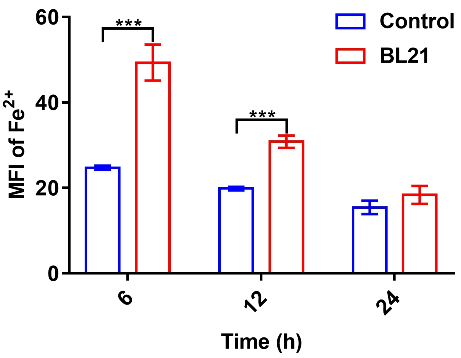

Figure S21. Mean fluorescence intensity of ferrous iron levels measured with a confocal microscope (Zeiss LSM980) according to images in Figure 5F. n = 3, ***$p < 0.001$.

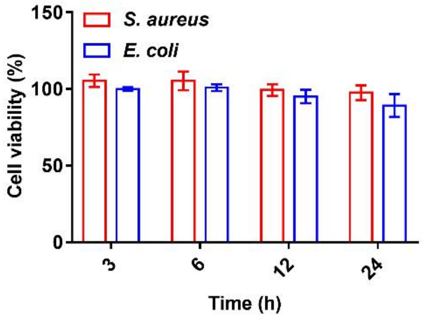

Figure S22. CCK8 assay for RAW246.7 cells activity following *S. aureus* and *E. coli* infection.

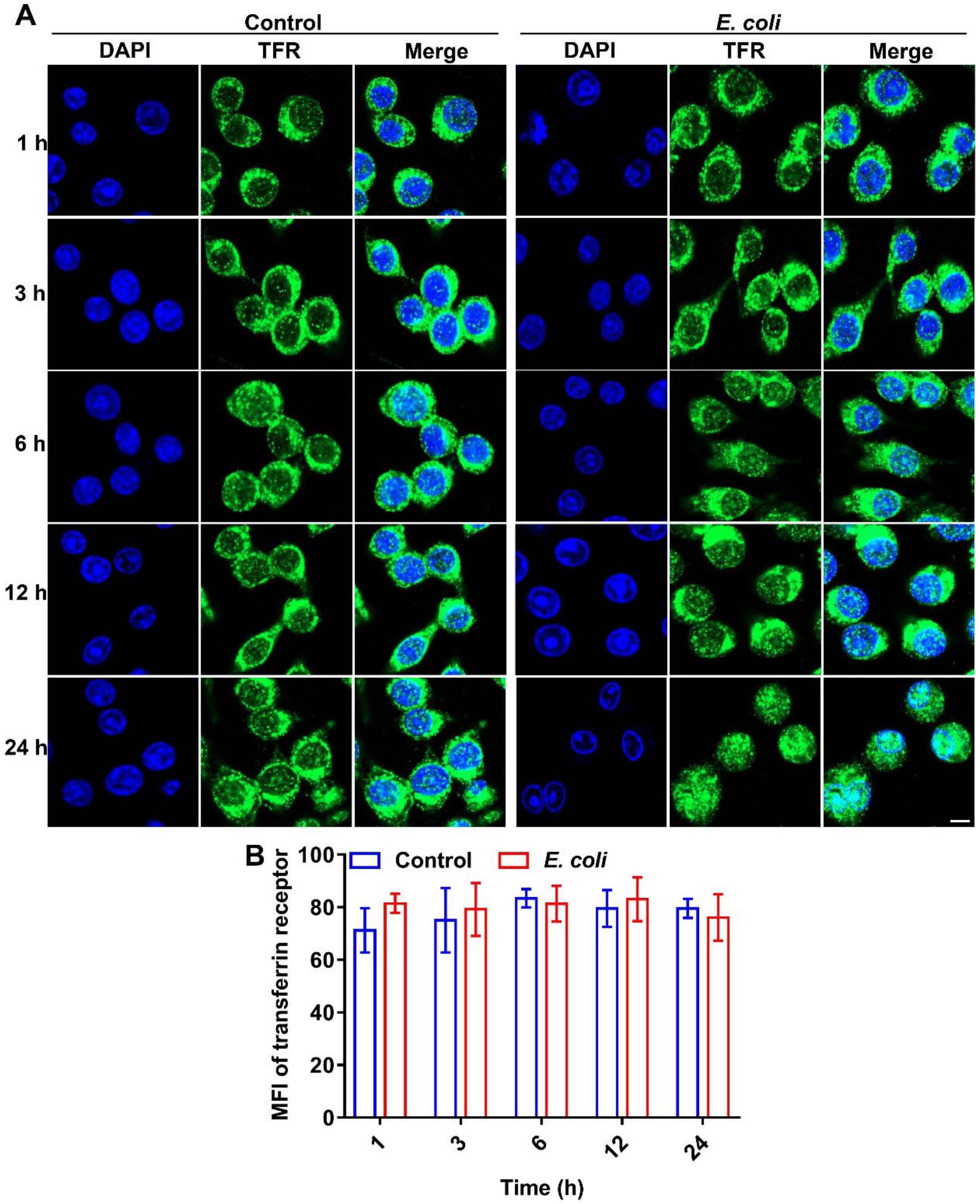

Figure S23. The change of transferrin receptor levels in macrophages infected by *E. coli*. (A) Confocal images of transferrin receptor. DAPI-stained cell nucleus. Transferrin receptor antibody-stained ferritin. Scale bar = 5 μm. **(B)** Mean fluorescence intensity of transferrin receptor measured with a Zeiss confocal microscope LSM980.

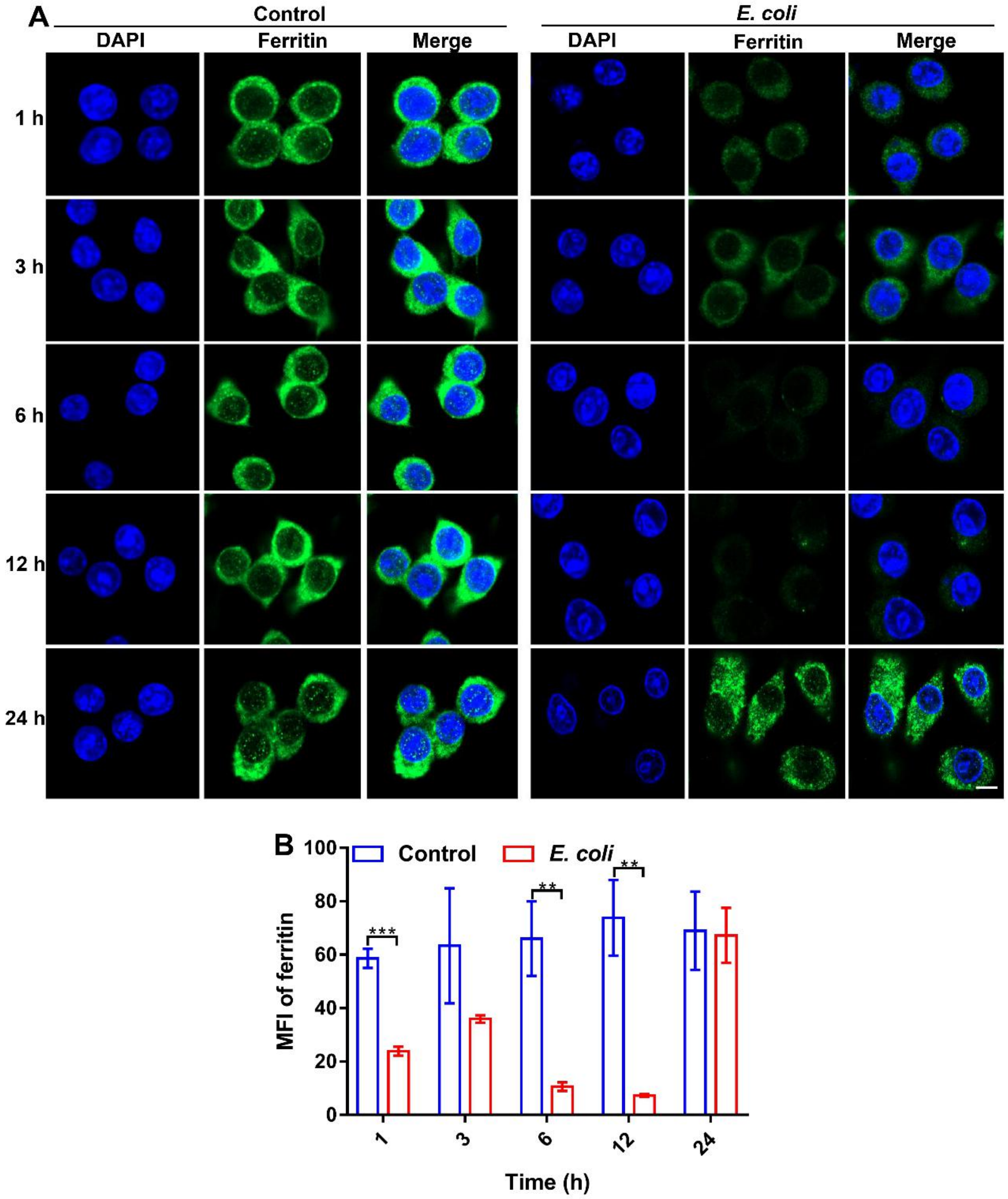

Figure S24. The change of ferritin levels in macrophages infected by *E. coli*. (A) Confocal images of ferritin. DAPI-stained cell nucleus. Ferritin antibody-stained ferritin. Scale bar = 5 µm. **(B)** Mean fluorescence intensity of ferritin measured with a Zeiss confocal microscope LSM980. n = 3, $^{**}p < 0.01$, $^{***}p < 0.001$.

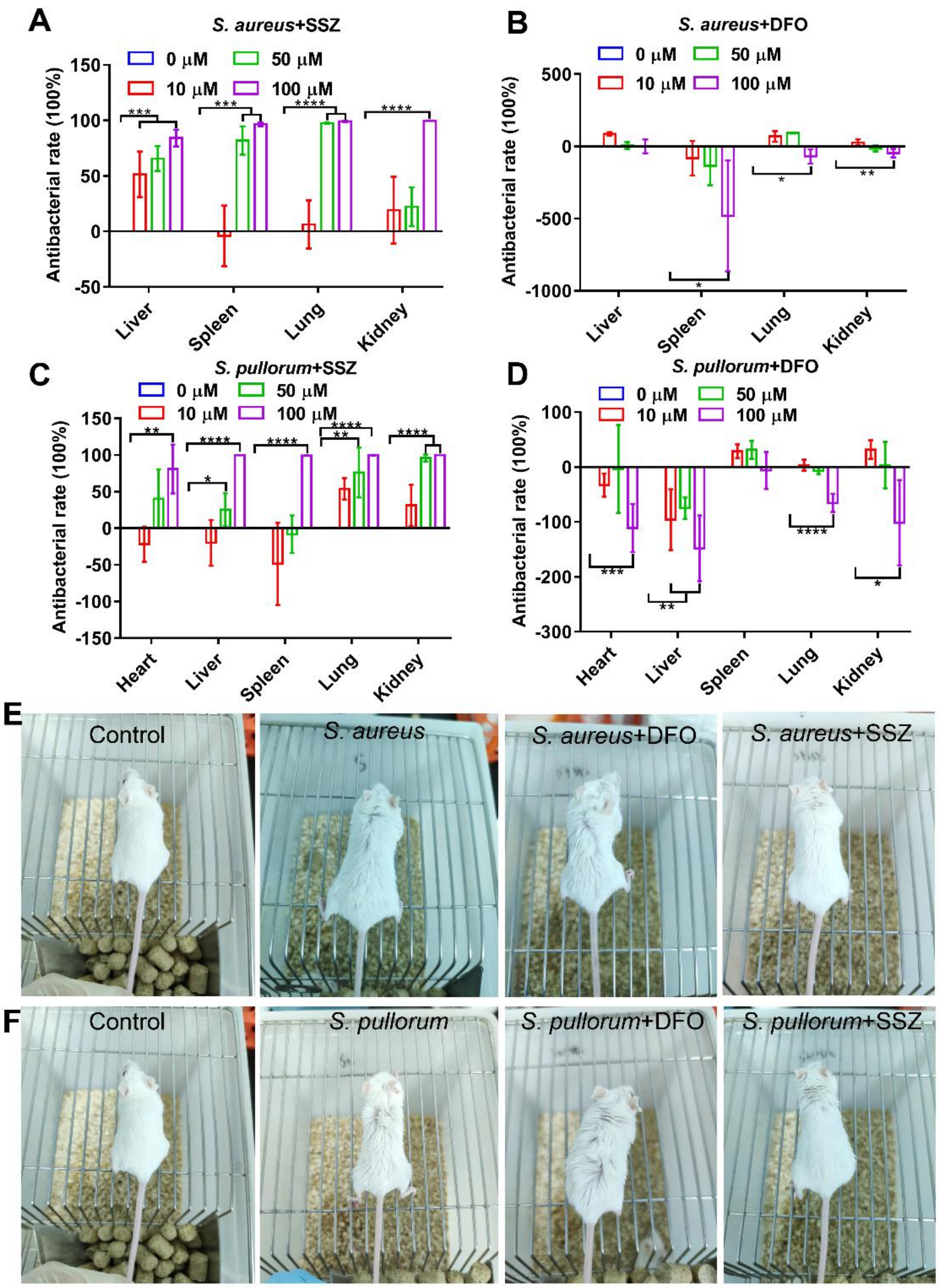

Figure S25. ***In vivo*** **treatment using SSZ or DFO in mice infected with planktonic bacteria.** **(A)-(B)** Bacterial inhibition efficiency of *S. aureus* in liver, spleen, lung and kidney treated with SSZ (10, 50, and 100 μM) or DFO (10, 50, and 100 μM) via tail vein at 72 h. **(C)-(D)** Bacterial inhibition efficiency of *S. pullorum* in heart, liver, spleen, lung and kidney treated with SSZ (10, 50, and 100 μM) or DFO (10, 50, and 100 μM) via tail vein at 72 h. **(E)** The appearance of Balb/C mice with *S. aureus* and treated by SSZ (100 μM) or DFO (100 μM) via tail vein. **(F)** The appearance of Balb/C mice with *S. pullorum* and treated by SSZ (100 μM) or DFO (100 μM) via tail vein. n = 5 mice per group. **p < 0.05*, ***p < 0.01*, ****p < 0.001*, *****p < 0.0001*.

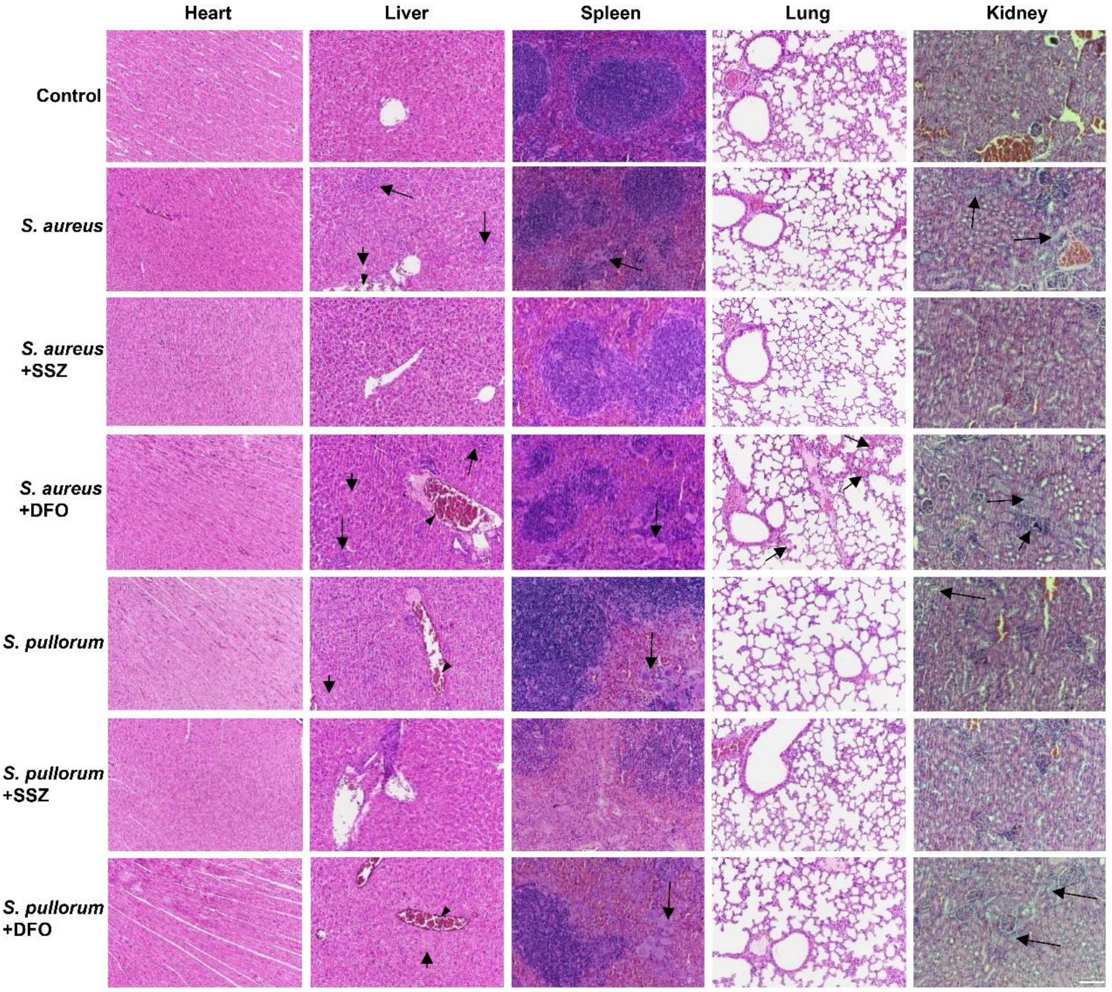

Figure S26. Histopathological analyses of organs after treatment with SSZ or DFO in mice infected with planktonic bacteria. (H&E, ×200), Scale bar = 5 μm.

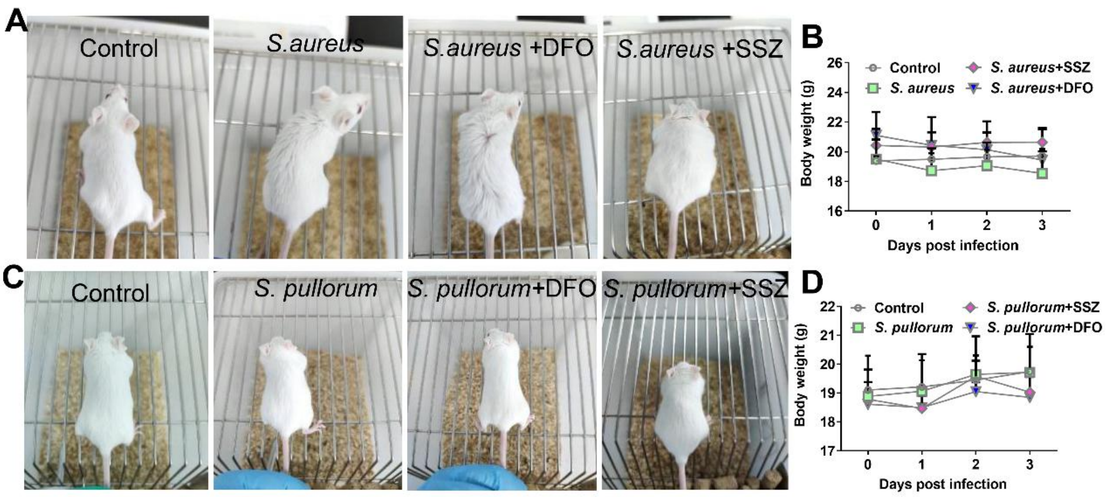

Figure S27. ***In vivo*** **intracellular bacterial infection treatment using SSZ or DFO. (A)** The

appearance of Balb/C mice with intracellular *S. aureus* and treated by SSZ or DFO via tail vein. **(B)** The change of body weight in Balb/C mice infected with intracellular *S. aureus*. **(C)** The appearance of Balb/C mice with intracellular *S. pullorum* and treated by SSZ or DFO via tail vein. **(C)** The change of body weight in Balb/C mice infected with intracellular *S. pullorum*. n = 5 mice per group.

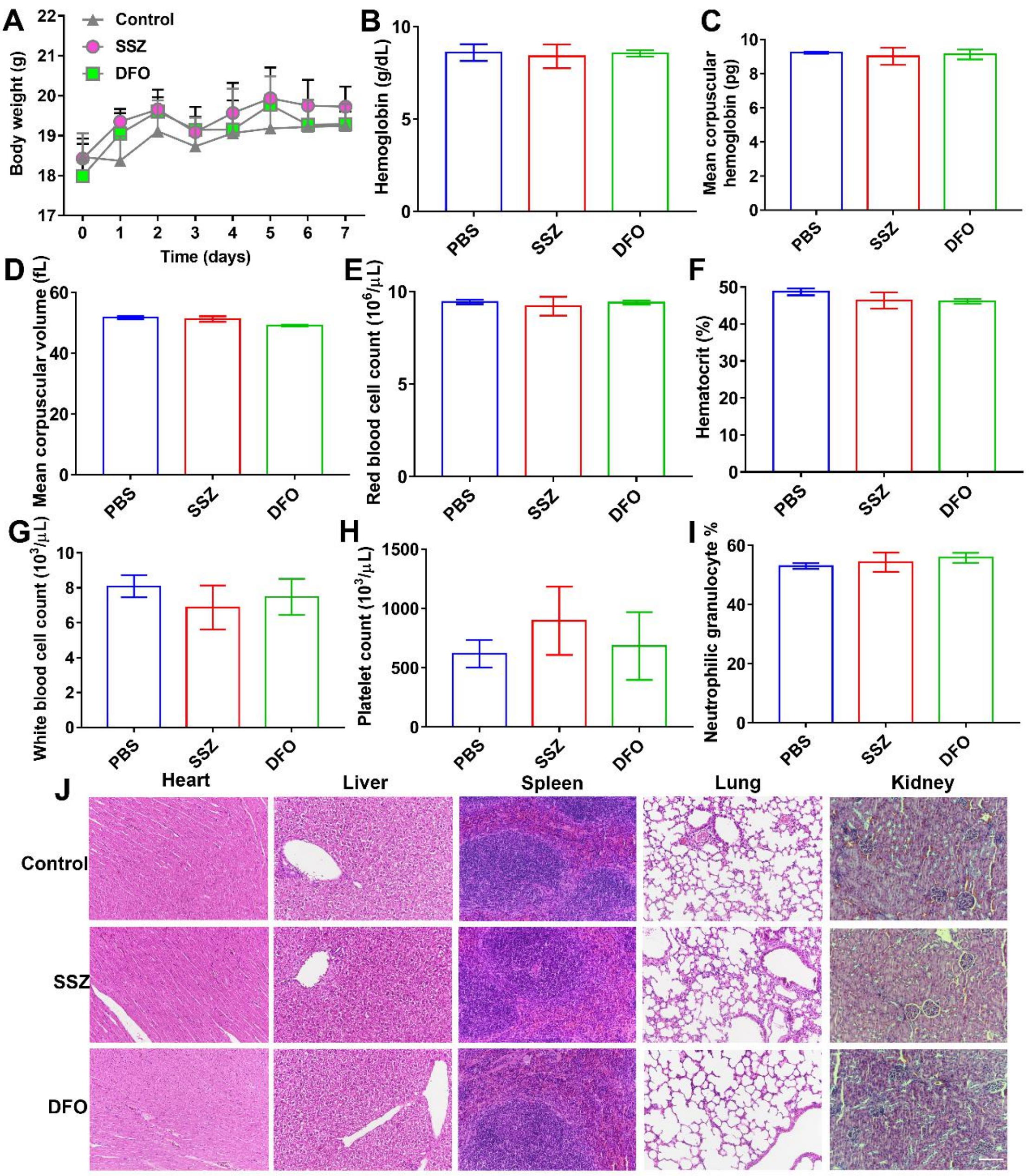

Figure S28. Evaluation of the biosafety of SSZ and DFO using *in vivo* models. (A) Body weight changes in Balb/C mice (n = 5) after tail vein injection of PBS, SSZ and DFO. **(B)** Hemoglobin levels in each group of mice during the treatment cycle. **(C)** Mean corpuscular hemoglobin levels in each group of mice during the treatment cycle. **(D)** Mean corpuscular volume levels in each group of mice during the treatment cycle. **(E)** Red blood cell levels in each

group of mice during the treatment cycle. **(F)** Hematocrit levels in each group of mice during the treatment cycle. **(G)** White blood cell levels in each group of mice during the treatment cycle. **(H)** Platelet count levels in each group of mice during the treatment cycle. **(I)** Neutrophilic granulocyte levels in each group of mice during the treatment cycle. **(J)** Histopathologic analysis. (H&E, ×200), Scale bar = 5 μm.

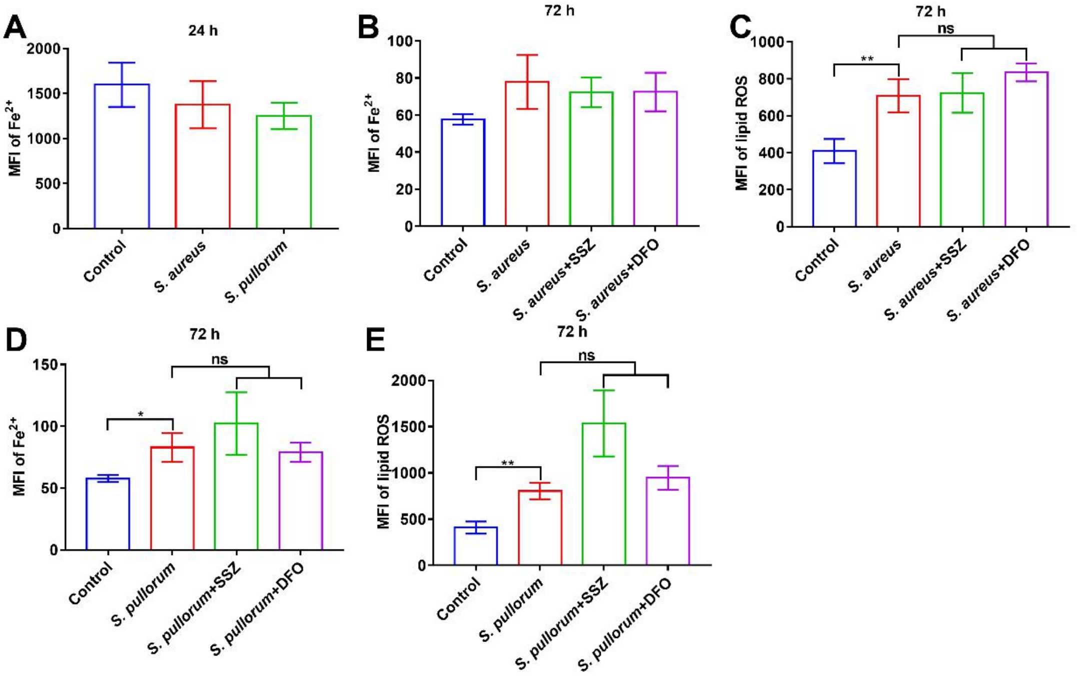

Figure S29. Lipid ROS and ferrous iron levels in peripheral blood macrophages after *S. aureus* or *S. pullorum* infection. **(A)** Ferrous iron levels in peripheral blood macrophages at 24 h after infection. Peripheral blood was taken from mice 24 h after infection and red blood cell lysis buffer was used to lyse erythrocytes. Peripheral blood macrophages and ferrous iron were labelled with FeRhoNox™-1 probes, respectively. Ferrous changes in peripheral blood macrophages were detected by flow cytometry. **(B)** Ferrous iron levels in peripheral blood macrophages at 72 h after *S. aureus* infection. **(C)** Lipid ROS levels in peripheral blood macrophages at 72 h after *S. aureus* infection. **(D)** Ferrous iron levels in peripheral blood macrophages at 72 h after *S. pullorum* infection. **(E)** Lipid ROS levels in peripheral blood macrophages at 72 h after *S. pullorum* infection. n = 5, ns = no significant, $*p < 0.05$, $**p < 0.01$.
